# A Virulent Strain of Deformed Wing Virus (DWV) of Honeybees (*Apis mellifera*) Prevails after *Varroa destructor*-Mediated, or *In Vitro*, Transmission

**DOI:** 10.1371/journal.ppat.1004230

**Published:** 2014-06-26

**Authors:** Eugene V. Ryabov, Graham R. Wood, Jessica M. Fannon, Jonathan D. Moore, James C. Bull, Dave Chandler, Andrew Mead, Nigel Burroughs, David J. Evans

**Affiliations:** 1 School of Life Sciences, University of Warwick, Coventry, United Kingdom; 2 Warwick Systems Biology Centre, University of Warwick, Coventry, United Kingdom; 3 Life Sciences & Warwick Crop Centre, University of Warwick, Wellesbourne, Warwickshire, United Kingdom; Stanford University, United States of America

## Abstract

The globally distributed ectoparasite *Varroa destructor* is a vector for viral pathogens of the Western honeybee (*Apis mellifera*), in particular the Iflavirus Deformed Wing Virus (DWV). In the absence of *Varroa* low levels DWV occur, generally causing asymptomatic infections. Conversely, *Varroa*-infested colonies show markedly elevated virus levels, increased overwintering colony losses, with impairment of pupal development and symptomatic workers. To determine whether changes in the virus population were due *Varroa* amplifying and introducing virulent virus strains and/or suppressing the host immune responses, we exposed *Varroa*-naïve larvae to oral and *Varroa*-transmitted DWV. We monitored virus levels and diversity in developing pupae and associated *Varroa*, the resulting RNAi response and transcriptome changes in the host. Exposed pupae were stratified by *Varroa* association (presence/absence) and virus levels (low/high) into three groups. *Varroa*-free pupae all exhibited low levels of a highly diverse DWV population, with those exposed *per os* (group NV) exhibiting changes in the population composition. *Varroa*-associated pupae exhibited either low levels of a diverse DWV population (group VL) or high levels of a near-clonal virulent variant of DWV (group VH). These groups and unexposed controls (C) could be also discriminated by principal component analysis of the transcriptome changes observed, which included several genes involved in development and the immune response. All *Varroa* tested contained a diverse replicating DWV population implying the virulent variant present in group VH, and predominating in RNA-seq analysis of temporally and geographically separate *Varroa*-infested colonies, was selected upon transmission from *Varroa*, a conclusion supported by direct injection of pupae *in vitro* with mixed virus populations. Identification of a virulent variant of DWV, the role of *Varroa* in its transmission and the resulting host transcriptome changes furthers our understanding of this important viral pathogen of honeybees.

## Author Summary

Honeybees are the most important managed pollinating insect, contributing billions of dollars to annual global agricultural production. Over the last century a parasitic mite, *Varroa*, has spread worldwide, with significant impacts on honeybee colony health as a consequence of its transmission of a cocktail of viruses while feeding on honeybee ‘blood’. The most important virus for colony health is deformed wing virus (DWV), high levels of which cause developmental deformities and premature ageing resulting in high overwintering colony losses. In experiments on individual *Varroa*-exposed pupae we demonstrate that a single type of virulent DWV is amplified 1,000–10,000 times in the recipient pupae, despite the mite containing a high diversity of replicating DWV strains. We could recapitulate this by direct injection of pupae with mixed virus populations, showing the virulent strain is advantaged by the route of transmission. In parallel, we detected changes in the immune response and developmental gene expression of the honeybee and propose that these contribute to the characteristic pathogenesis of DWV. Identification of a virulent strain of DWV has implications for therapeutic or prophylactic interventions to improve honeybee colony health, as well as contributing to our understanding of the biology of this important honeybee viral pathogen.

## Introduction

Host-pathogen interactions can be broadly divided into asymptomatic or symptomatic infections [1]. In the former, the absence of symptomatic disease is typically due to restricted pathogen replication, which reduces the opportunities for horizontal transmission within its host population. Conversely, prolonged survival of the infected host increases the likelihood of vertical transmission of the pathogen [2]. In contrast, symptomatic infections are typically characterized by high levels of pathogen replication, with consequent enhanced virulence, thereby maximizing horizontal transmission [1]–[4]. The ‘lifestyle choice’ of asymptomatic or symptomatic infection is determined by multiple factors including the duration of host-pathogen co-evolution, host physiology and anti-pathogen responses, routes of transmission and environmental factors. Evolutionary changes in pathogen virulence may be triggered by changes in pathogen-host assemblages [5]. In the case of multi-host pathogens with interspecies transmission, a pathogen's virulence may increase following introduction of a second host, when the constraint on pathogen virulence in a given host is removed [6].

The European honeybee (*Apis mellifera*) is the predominant managed pollinating insect and delivers economically important pollination services for agriculture which are estimated to add ∼$40bn globally to crop value/annum [7]. Factors that influence colony health and viability are therefore important for colony survival and pollination performance. In addition to the bacterial foulbroods, the most important diseases of *A. mellifera* are caused by a range of viruses many of which are vectored by the ectoparasitic mite *Varroa destructor* when feeding on honeybee haemolymph. *Varroa* is believed to have expanded its host range from *Apis cerana* to *A. mellifera* during the first half of the 20^th^ century and subsequently spread to all beekeeping regions of the world with the exception of Australia [8]–[11].

Deformed wing virus (DWV), a picorna-like single-stranded, positive-sense, RNA virus [12], [13], is present in the majority of honeybee colonies [10]. DWV is closely related to Varroa destructor virus type 1 (VDV-1) [14]. Their recombinants [15], [16] and Kakugo virus (KV) [17], which together exhibit at least 84% nucleotide identity, can be considered as strains of the same virus (henceforth we use the term DWV to refer to this related group of viruses). In the absence of *Varroa*, DWV generally causes asymptomatic infection and is present at low levels in honeybees. In contrast, in *Varroa*-infested colonies, mite-exposed pupae can exhibit very high DWV levels which may result in impaired development of the teneral adult honeybee and increased mortality in all honeybees in these colonies, including asymptomatic adults [10], [13]. The mechanisms underlying the transition of DWV from a relatively benign virus to a major honeybee pathogen in the presence of *Varroa* remain unclear. Two possibilities, not mutually exclusive, have been proposed: suppression of honeybee antivirus defences by *Varroa* mites which allows the virus to proliferate [18], [19], and a *Varroa*-driven selection of particular DWV genotypes, potentially due to replication in the mite [15], [20].

Previous studies using functional or gene expression analysis have produced contradictory conclusions on the impact of *Varroa* on the immune responses of honeybees. Initial reports indicated that *Varroa*-exposed honeybees were immuno-compromised [18], [19], although later transcriptome analysis found little or no effect on genes implicated in insect immunity [21], [22]. Additional studies have shown down-regulation of a honeybee NF-κB transcription factor [23]. Recent reports have implicated the *Drosophila* Toll, Imd and Jak-Stat signalling pathways in controlling RNA virus infection [24] and RNA interference (RNAi), which has long been considered the major antiviral mechanism in insects [25], has recently been associated with controlling the persistence of RNA virus infections in *Drosophila* [26]. It was therefore possible that high levels of DWV in *Varroa*-exposed honeybees could be the result of a suppression of these antivirus responses and so warranted further analysis.

We have previously demonstrated that *Varroa* infestation is associated with the accumulation in mite-exposed pupae of a particular subset of DWV-like viruses [15]. These recombinant forms (RF) are predominantly comprised of genomes with structural and non-structural coding regions that most closely align with VDV-1 and DWV respectively. The organisation of these recombinants suggests that, as with other picorna-like viruses, DWV likely has a modular genome, with a 5′ untranslated region (5′-UTR) driving translation of the structural or capsid (CP) and non-structural (NS) ‘modules’ [15]. We hypothesised that such recombinants were transmitted more efficiently between *Varroa* and honeybees, resulting in their amplification to the markedly elevated levels observed in *Varroa*-parasitized pupae (about 1000 times higher than in unexposed pupae). In recent complementary studies, changes in the composition of the DWV population over a large temporal and spatial scale following *Varroa* infestation were reported for honeybees colonies following accidental introduction of *Varroa* into the Hawaiian islands [20]. The introduction of *Varroa* was associated with a marked restriction in DWV diversity measured in the pooled honeybee samples collected from the *Varroa*-infested colonies, although the precise identity of the dominant virus was not determined [20].

In the present study we devised a novel experimental system to specifically test two hypotheses on the role of *Varroa* in the development of high-level DWV infection in the honeybee, namely that the mite (i) amplifies and transmits virulent genotypes of DWV, and (ii) suppresses antiviral responses, including immune signalling pathways and RNA interference. The experimental procedure included exposure of *Varroa*-naïve honeybees to mites and their associated DWV payload together with the *per os* in-hive horizontal transmission. The use of *Varroa*-naïve honeybees from a *Varroa*-free region allowed us to monitor changes in DWV diversity and loads, as well as potential antivirus responses in the honeybee responses, following exposure to the viral genotypes associated with *Varroa* infestation. Importantly, we analysed immune responses and viral load/diversity in individual mite-exposed and –unexposed pupae, rather than in pooled samples. This allowed us to stratify individual responses into four distinct experimental groups, characterised by *Varroa* exposure and viral load, that clearly correlated with characteristic changes in the transcriptome and virus population diversity. In addition, we recapitulated the exposure of *Varroa*-free honeybees to DWV by direct injection and analysed virus diversity in bees of a colony with long-established *Varroa* infestation.

Our results indicate that a virulent recombinant form of DWV, while transmissible orally, only replicates to high levels when directly inoculated into honeybee haemolymph – by *Varroa* or experimental injection. This results in massive reduction of DWV diversity in bees with high virus levels, both in the *Varroa*-exposed pupae and newly emerged bees with symptomatic deformed wing disease. Significantly, the same virulent recombinant form of DWV reached the highest levels in mite-exposed pupae and in adult bees exhibiting characteristic deformed wing symptoms. Although exposure to *Varroa* resulted in changes in expression of a number of immune-related genes, the roles of which should be further explored, we demonstrate that it is the route of virus acquisition that is responsible for the amplification of this virulent form of DWV in a *Varroa*-infested colony.

## Results

### Experimental infestation by *Varroa* mites results in bimodal DWV levels

**Figure 1:**
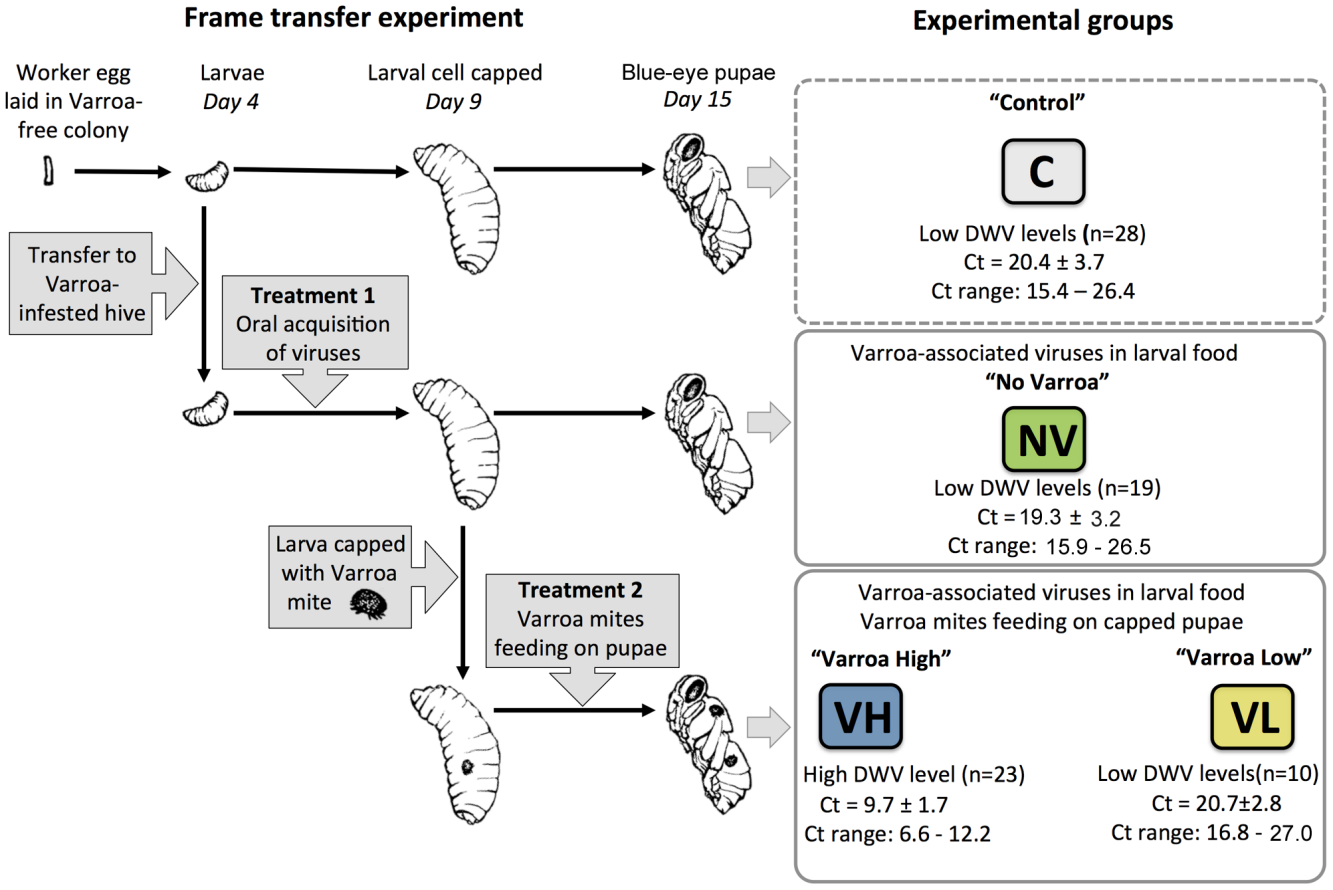
Design of the frame transfer experiment, summary of treatments, and experimental honeybee groups. Shown are treatments of the experimental groups of honeybees originated in a single colony, number (*n*) of individuals, and the results of DWV-like virus quantification in the individual pupae by real-time PCR (average Ct value ± standard deviation (SD) and the range of Ct values).

Worker honeybee larvae from a *Varroa*-free colony (sourced from a region with no historic contacts with or presence of *Varroa*) were moved in a frame transfer experiment to a *Varroa*-infested colony. The larvae were subsequently exposed through feeding to DWV strains circulating in the infested colony from day 4 until the cells were capped at day 9 (all times relative to egg laying; Figure 1, Treatment 1). *Varroa* mites enter brood cells immediately prior to capping. Therefore, pupae located within brood cells that contain *Varroa* mites are also subjected to the mite feeding on haemolymph during pupal development (Figure 1, Treatment 2) until sampling on day 15 (the purple-eye stage), six days after cell capping. Feeding of the mites (adult females) on pupae was confirmed by the presence of at least one protonymph in the capped cell [27].

We assessed the total levels of DWV viruses in 80 individual pupae by qRT-PCR using a primer pair for a conserved polymerase-coding region, designed to detect all known DWV strains, including DWV, VDV-1 and KV (Table S1). The real-time PCR Ct values showed a clear bimodal distribution, with distinct low- and high-levels of DWV (*p*<10^−16^; Figure 1, Figure S1). Low DWV levels were observed in all (*n* = 23) sampled pupae maintained in the *Varroa*-free colony (group C, “Control”), in all 19 sampled pupae transferred to the *Varroa*-infested colony that were not capped with a *Varroa* mite and therefore subjected only to Treatment 1 (oral DWV infection; group NV, “No *Varroa*”), and in 10 of 33 pupae upon which *Varroa* mites had fed, Treatment 2 (group VL, “*Varroa* Low”). In contrast, high levels of DWV-like viruses were detected in the remaining 23 of 33 *Varroa*-associated pupae, which experienced both Treatment 1 and Treatment 2 (group VH, “*Varroa* High”). The Ct ranges for the VH group lay entirely below the VL range, indicating significantly higher virus levels in VH (Figure S1A) whereas the Ct values in groups C, NV and VL were statistically indistinguishable (ANOVA, *p* = 0.5261). We have previously reported similar proportions of *Varroa*-associated pupae with low and high levels of DWV in an independent (temporally and geographically) study [15]. These results indicate that direct *Varroa* exposure does not inevitably lead to high, presumed pathogenic, DWV levels, as reported previously [15], [28], [29], at least when age-matched, synchronously exposed pupae are analysed individually. The difference in DWV levels between pupae in the VL and VH groups could not be explained by different mite loads - both contained an average of 1.375 adult female *Varroa* mites per cell (data not shown). These two distinct classes of *Varroa*-exposed pupae, and their associated mites, were included as separate groups in subsequent analyses to investigate host or parasite determinants that influenced the outcome of exposure.

We sampled eight honeybee pupae selected at random from each of the four groups (C, NV, VH and VL; Figure 1) for further analysis. With the exception of the siRNA responses (for which pooled samples of each of the four groups were used), subsequent analysis of transcriptional responses (microarray transcriptional profiling) and virus diversity (qRT-PCR, cloning and sequencing) were conducted individually on each of the eight pupae from the four response groups.

### Significant changes to the honeybee transcriptome are characteristic of experimental groups and *Varroa*/virus exposure

**Figure 2:**
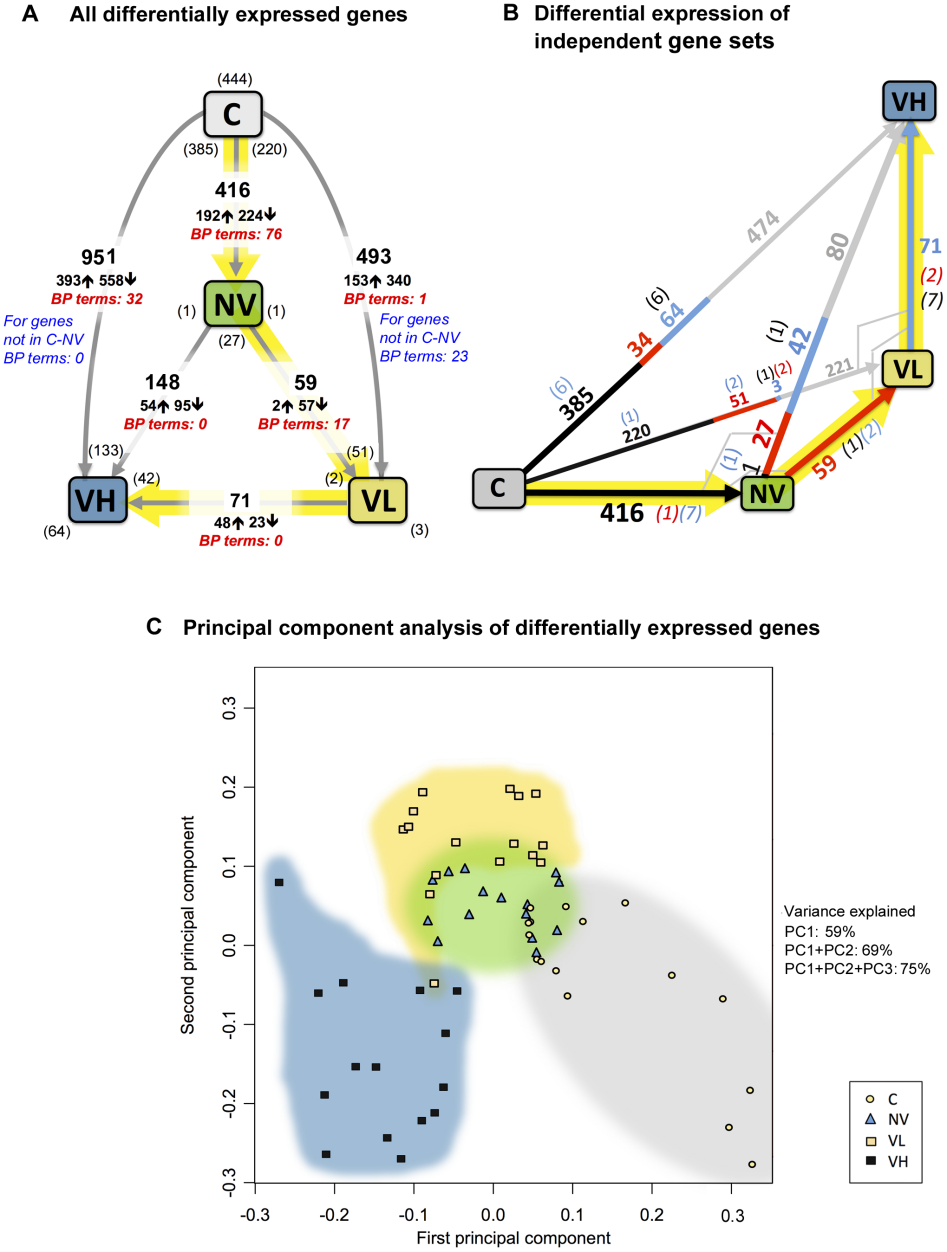
Summary of the gene expression changes in the experiment. (A) Total number of differentially expressed (DE) genes in the contrasts. The numbers of up-regulated and down-regulated genes in each contrast are marked, respectively, as ↑ and ↓. An up-regulated gene level is higher at the head of the arrow showing the contrast; commonality is shown in brackets. The numbers of overrepresented GO Biological Process terms associated with the DE genes are shown in red italic characters for each contrast. (B) A geometrical visualization of the three-stage experimental process: shown are, with numbers of differentially expressed genes, the “orthogonal” stages, contrasts C to NV (black), NV to VL (red), VL to VH (blue), and the commonalities in the composite stages shown in the colour of the “orthogonal” contrast. The DE gene numbers in the composite contrasts without commonalities to the “orthogonal” stages are shown in grey. Commonalities between orthogonal stages are shown in corresponding colour in brackets. (C) Result of principal component analysis applied to a set 60 DE genes (pooled from all contrasts) with low adjusted *p*-values. Shown is a plot of the first two principal component scores for Cy3 and Cy5 replicates for all honeybee samples.

We used a two-colour dye-balanced loop design microarray [30], [31] to determine the genome-wide transcriptional profile using RNA extracted from the 32 samples defined above, (8 pupae from each experimental group). The oligonucleotide expression array contained probes to all protein-coding transcripts of *A. mellifera* [32], as well as probes to all known viral and fungal pathogens of honeybees, including distinct DWV and VDV-1 probes. After array normalization, differentially expressed (DE) genes were determined for each contrast between experimental groups (Figure 2A, listed in Table S2 with commonalities between contrasts shown in Table S3). Microarray results were validated by qRT-PCR using oligonucleotide primers to a set of honeybee DE genes and the constitutively expressed ribosomal protein 49 (Rp49) gene (GB10903; Table S1), showing strong positive correlations between the processed microarray signals and normalized Ct values (Pearson correlation coefficients between 0.504 and 0.873). Additionally, there was a strong positive correlation between the DWV microarray signal and qRT-PCR Ct values for DWV-like viruses using generic DWV primers (Table S1, Primers 59 and 60), Pearson correlation coefficient 0.797. Other than DWV-like viruses, no other honeybee pathogens were detected.

There were high levels of commonality and additivity for DE genes in the contrasts considered (Figure 2A, Figure S2 A, C). For example, the C to VH contrast (in which ∼10% of genes were DE) can be decomposed into two sub-contrasts by exposure regime, *i.e.* split C to VH at oral exposure (NV) or at mite feeding (VL). Similarly the C to VL contrast can be split at NV. These decompositions exhibit high orthogonality (Figure 2 A,B; Figure S2 B,C). This suggests that expression of essentially different sets of genes are influenced following oral exposure to DWV, *Varroa* feeding, and the markedly elevated levels of DWV in *Varroa*-exposed pupae. To explore this further we conducted principal component analysis (PCA). Distinct clustering by experimental group was observed when two independent sets of DE genes with the lowest *p*-values were analysed *i.e.* those from the DE genes pooled from all contrasts (Figure 2C), or DE genes in each of six contrasts (Figure S3). Consequently, PCA strongly suggests that the experimental groups exhibit characteristic gene expression signatures reflecting their fate after exposure in a *Varroa*-infested colony.

To obtain insight into the functional consequences of DE gene expression we carried out Gene Ontology (GO) analysis, focusing on the GO Biological Process (BP) [33]. A number of overrepresented GO BP terms related to cell division were associated with DE genes in the C to NV contrast, while those related to regulation of various cellular processes were associated with the DE genes in the NV to VL contrast (Figure 2A, Table S4). Notably, no overrepresented GO BP terms were associated with the genes DE following increase of DWV levels (VL to VH contrast). We then looked in detail at the expression patterns of likely immune-related genes as it had previously been reported that *Varroa* and/or viruses could influence honeybee immunity [18], [19], [23]. The list of 381 putative honeybee immune-related genes included those previously published [34], [35] together with honeybee homologs of the *Drosophila* genes associated with the GO term “Immune system process” (GO: 0002376). The C to VH and C to VL contrasts exhibited the highest number of DE immune-related genes (*n* = 42 and n = 26 respectively, 22 of the latter also being in the C to VH contrast), whereas oral exposure (C to NV) resulted in 18 DE immune-related genes (Table 1, Figure S4). Independent confirmation of DE of immune-related genes was obtained by qRT-PCR analysis of persephone protease (GB14044), Tollo (GB10640), and Vago (GB10896) with Pearson correlation coefficients of 0.598, 0.504 and 0.692 respectively.

**Table 1:** Differential expression of the honeybee immune-related genes in response to oral DWV and *Varroa* mite feeding.

BeeBase ID^a^	Drosophila homolog ID^b^	Gene^c^	Pathway, category^d^	Fold change in contrast					
				C to NV	C to VH	C to VL	NV to VH	NV to VL	VL to VH
GB10896	FBgn0051997	Vago	antivir	1.860	1.468	1.833	-	-	-
GB10640	FBgn0029114	Tollo	Toll	-	−0.452	-	-	-	-
GB17781	FBgn0036494	Toll	Toll	-	−0.215	-	-	-	-
GB15688	FBgn0003495	spatzle	Toll	-	−0.381	-	−0.249	-	−0.346
GB17879	FBgn0030310	PGRP-S3	Toll	-	−0.564	-	−0.381	-	−0.381
GB14044	FBgn0030051	spirit	Toll	-	−0.701	-	-	-	-
GB19582	FBgn0028984	NEC-like	Toll	0.482	0.642	0.475	-	-	-
GB13935	FBgn0261988	Gprk2	Toll	-	−0.322	−0.311	−0.323	−0.312	-
GB19452	FBgn0243514	GNBP3	Toll	0.800	1.599	0.931	-	-	-
GB19961	FBgn0040323	GNBP1	Toll	-	−0.400	−0.289	-	-	-
GB19066	FBgn0260632	dorsal	Toll	-	0.202	-	-	-	-
GB17654	not found	SP45	SP	-	−0.535	−0.669	-	-	-
GB16214	FBgn0036287	SP38	SP	-	−0.214	-	−0.284	−0.236	-
GB14309	FBgn0033359	SP33	SP	1.138	1.277	1.153	-	-	-
GB11511	FBgn0038595	SP32	SP	1.667	-	1.395	-	-	-
GB11743	FBgn0035290	AmSCR	Scav	0.806	0.511	-	-	-	-
GB10506	FBgn0058006	AmSCR	Scav	−0.359	−0.369	−0.350	-	-	-
GB15155	FBgn0004197	Serrate	Notch	-	−0.422	−0.538	-	−0.324	-
GB13135	FBgn0014020	Rho1	JNK	-	−0.343	−0.314	-	-	-
GB12838	FBgn0015286	Ras	JNK	−0.217	−0.302	-	-	-	-
GB19901	FBgn0243512	puckered	JNK	0.600	0.441	-	-	-	-
GB12212	FBgn0001297	kayak	JNK	0.371	0.371	-	-	-	-
GB12004	FBgn0001291	Jun	JNK	0.450	0.471	-	-	-	-
GB16422	FBgn0004864	hopscotch	J-ST	-	−0.204	-	-	-	-
GB19988	FBgn0025827	Lysozyme	Immune	−0.315	−0.498	−0.386	-	-	-
GB18918	FBgn0004606	zfh1	Immune	-	0.401	-	-	-	-
GB16457	FBgn0003963	u-shaped	Immune	-	-	−0.291	-	−0.371	-
GB15719	FBgn0086899	tlk	Immune	-	-	−0.254	-	−0.259	.
GB13670	FBgn0043550	Tsp68C	Immune	−0.708	−1.267	−0.881	-	-	-
GB12465	FBgn0003896	tailup	Immune	-	−0.455	−0.300	-	-	-
GB11411	FBgn0004837	Su(H)	Immune	-	−0.214	−0.257	-	-	-
GB12280	FBgn0039141	spastin	Immune	-	−0.318	-	-	-	−0.261
GB16613	FBgn0011823	Pendulin	Immune	−0.276	−0.312	−0.297	-	-	-
GB12373	FBgn0010247	Parp	Immune	−0.241	−0.420	-	-	-	-
GB10718	FBgn0085432	pangolin	Immune	-	−0.230	−0.223	-	-	-
GB17628	FBgn0262738	norpA	Immune	-	0.266	-	-	-	-
GB12005	FBgn0004657	mys	Immune	-	-	-	0.255	-	0.251
GB10124	FBgn0013576	mustard	Immune	-	−0.264	−0.281	−0.502	−0.519	-
GB13202	FBgn0040324	Ephrin	Immune	-	−0.336	−0.295	-	-	-
GB19881	FBgn0027066	Eb1	Immune	-	.	−0.208	-	−0.201	-
GB12454	FBgn0243514	eater	Immune	-	1.555	.	-	-	-
GB13459	FBgn0031464	Duox	Immune	0.791	0.619	0.498	-	-	-
GB19168	FBgn0011764	Dsp1	Immune	−0.222	−0.355	−0.216	-	-	-
GB14446	FBgn0259099	dcx-emap	Immune	-	−0.440	-	−0.679	-	-
GB17018	FBgn0087011	CG41520	Immune	0.499	0.461	0.503	-	-	-
GB14317	FBgn0021764	sidekick	IG	-	−0.215	-	-	-	-
GB19895	FBgn0003117	pannier	GATA	-	−0.760	−0.512	-	-	-
									

a Honeybee gene ID according to the *Apis mellifera* Official Gene Set 1 [30]. Honeybee immune-related genes included in the analysis were either those described in [33] or the honeybee homologues of the Drosophila melanogaster genes associated with Gene Ontology Biological Process term “Immune System Process” GO:0002376.

b Drosophila melanogaster homologue showing highest similarity in BLAST.

c Drosophila melanogaster gene name according to FlyBase.

d Pathway or category of gene if known (Toll - Toll signalling pathway; SP - serine protease; Scav - Scavenger receptor A; Notch - Notch signalling pathway; JNK - JNK signalling pathway; J-ST - JAK-STAT signalling pathway; IG - IG Superfamily Genes; GATA - GATA transcription factor), Immune - Immune system process gene.

### Next generation sequencing analysis of the RNAi response in DWV-infected pupae

**Figure 3:**
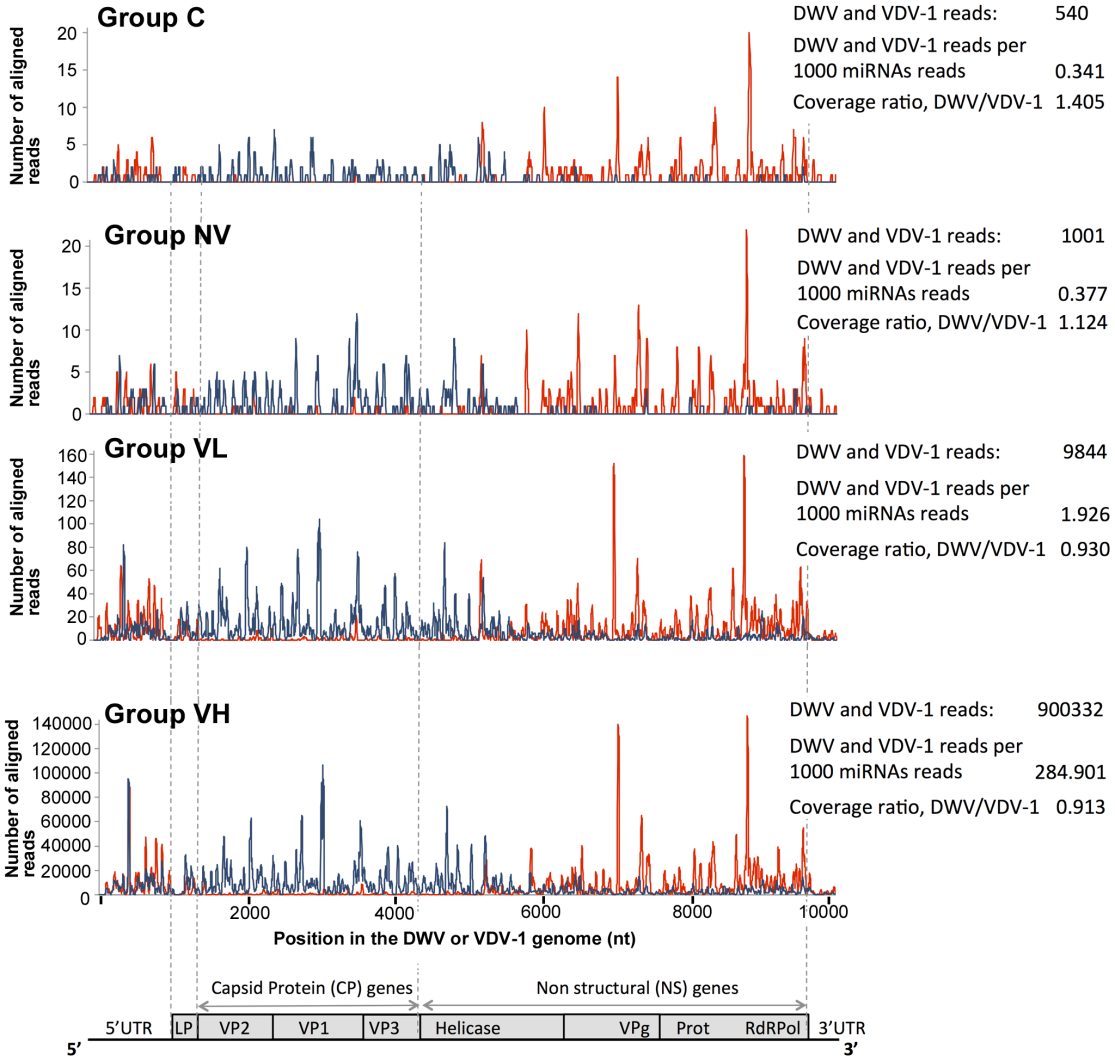
High-throughput sequencing of the honeybee small RNA libraries. The graphs show depth of coverage at the genomic loci of DWV (red) and VDV-1 (blue). A statistical summary of the reads is given to the right of each group. Only reads unambiguously aligning to DWV or VDV-1 were used (GenBank Accession numbers GU109335 and AY251269 respectively) with no mismatches being tolerated in the 18 nt. seed.

Although no significant changes in expression of genes associated with the RNAi response (*e.g.* Argonaute, Dicer) [25] were observed in the microarray analysis, there could be post-transcriptional effects on RNAi generation. We therefore analysed the DWV-related RNAi population and compared it with the levels and identity of virus in pupae from the four experimental groups (Figure 1). Small RNA fractions (15 to 40 nt) were isolated from total RNA samples, pooled according to the experimental groups and used as templates for Illumina high-throughput sequencing. One library was generated for the group C honeybees and two libraries for each of the other groups. These libraries, each containing 11 to 35 million reads, were aligned to the reference viral sequences (DWV and VDV-1, GenBank accession numbers GU109335 and AY251269 respectively; Figure 3), as well as to the honeybee miRNA sequences [36], using Bowtie [37].

**Figure 4:**
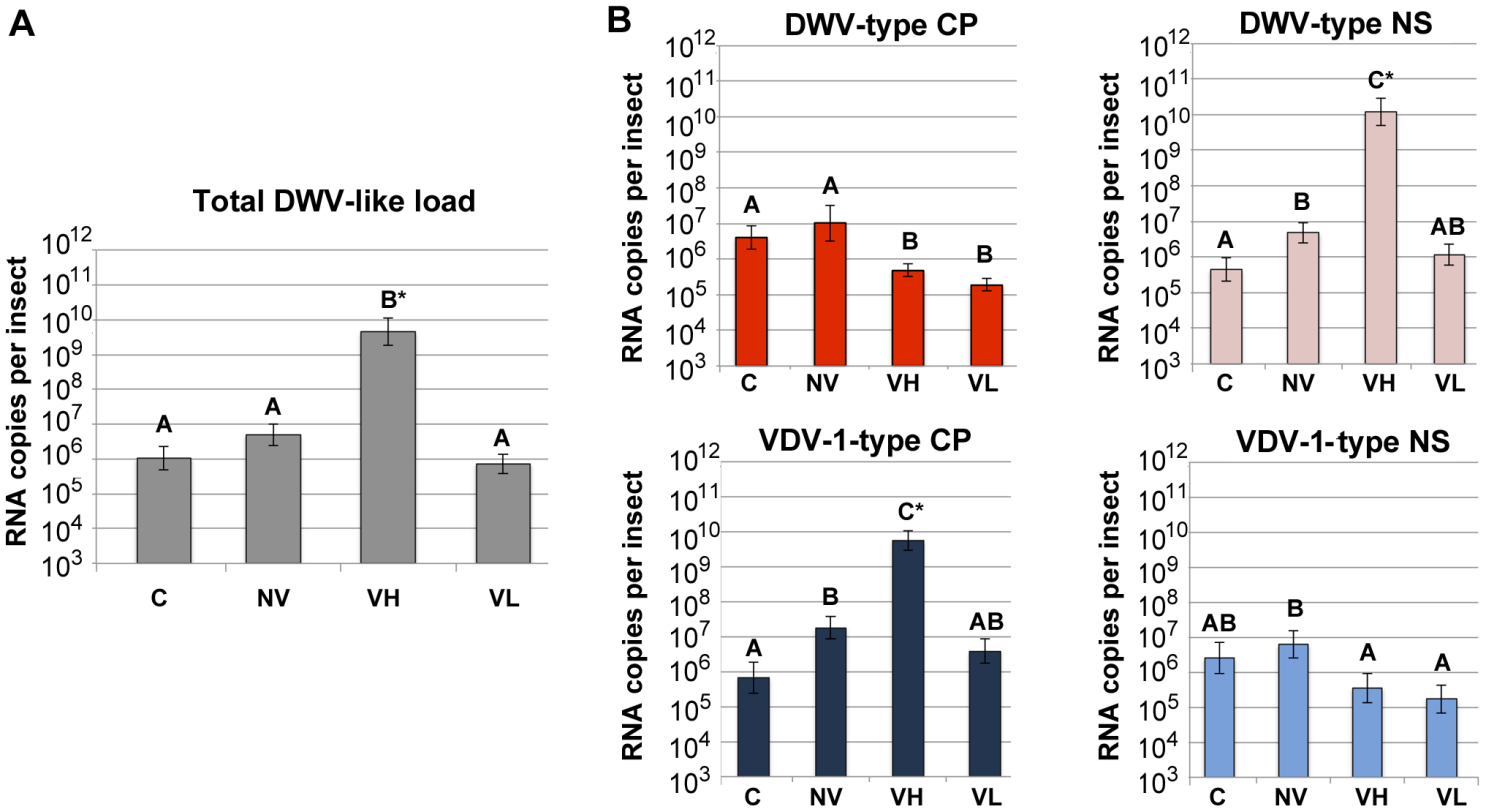
Total and strain-specific virus genome quantification in honeybee pupae. Quantification of the viral RNA by qRT-PCR in the honeybee pupae from the frame transfer experiment. Numbers of the viral RNA molecules per pupa (*n* = 8 for each experimental group) are shown. (A) Total DWV-like virus load quantified with the primers recognising the NS region of all DWV-like viruses (DWV, VDV-1, recombinants thereof and KV). (B) Quantification of the DWV CP, DWV NS, VDV-1 CP, and VDV-1 NS with the specific primers. Bars show mean value with standard error (SE). Letters above the bars represent statistically significant groupings according to pairwise *t*-test comparisons, *p*<0.05; asterisk marks *p*<0.0001.

All RNA libraries analysed contained similar proportions of host-encoded miRNA reads, 12 to 18% of the total (Table S5), indicating both successful isolation of small RNA libraries and broad equivalence of the pooled sample sets. DWV- and VDV-1-specific siRNAs of both polarities were present in all treatment groups. DWV- and VDV-1-specific siRNAs could originate from either DWV or VDV-1, or from the previously reported [15] recombinants between these parental viruses (Figure 3). Approximately 50% of all viral reads were 22 nt in length and 25% were 21 nt, with three to four times the number of sense orientation reads to antisense, irrespective of the read length (Table S5). To exclude variation due to the efficiency of library preparation, we normalised the siRNA number to the total number of honeybee miRNA reads in a library. The normalised loads of DWV/VDV-1-specific siRNA reads were similarly low in group C and the two NV group libraries (0.341, 0.377 and 0.397 siRNA per 1000 miRNAs respectively), ∼5 times higher in the two VL group libraries (1.926 and 2.066 siRNAs per 1000 miRNAs) which exhibited similar viral loads to groups C and NV (Figure 4A, see below), but markedly higher in the VH group samples (285 and 287 siRNA per 1000 miRNAs; Table S5). The profiles of the DWV-and VDV-1-specific siRNA coverage of the DWV and VDV-1 reference genomes (Figure 3) were most similar between groups VL and VH (Pearson correlation 0.955 to 0.963 for DWV, 0.945 to 0.962 for VDV-1, Table S6). The profiles for groups C and NV were more distinct from each other, and to VH or VL (Pearson correlation 0.593 to 0.786 for DWV, 0.399 to 0.726 for VDV-1; Table S6).

### Significant changes in DWV levels and diversity following oral DWV infection and *Varroa* mite feeding revealed by virus-specific qRT PCR

We and others have previously reported changes in virus diversity at the population level [20] and the predominance of particular virus recombinants (*i.e.* a reduction in diversity) in honeybee pupae exhibiting high viral loads [15]. To quantify both viral load and diversity in individual honeybee pupae and their associated *Varroa* mites we used generic NS qRT-PCR primers or primer pairs specific for DWV or VDV-1 CP or NS coding regions (Table S1). We quantified the total virus levels (Figure 4A) and the levels of the DWV-type and VDV-1-type CP and NS regions (Figure 4B) in each of the 32 pupae as well as in each of 15 *Varroa* mite samples co-isolated with the VH and VL group pupae.

As already indicated (Figure 1), the qRT-PCR Ct values used to separate the VH from the VL, NV and C experimental groups, indicated significant differences in viral loads in representative pupae (Figure 4A), with the VH group exhibiting at least 3 log_10_ higher levels of DWV-like viruses per pupa. When analysed using specific CP or NS primer pairs, the most pronounced difference was the increase in the number of genomes with the VDV-1 CP and the DWV NS sequences in the VH group pupae compared to the other treatment groups (Figure 4B). In comparison with the control group C, the VH group exhibited a 6,000-fold increase in the VDV-1 CP region and a 26,000-fold increase in the DWV NS coding region. When compared with the NV group, the VH group showed lower relative increases (312-fold for VDV-1 CP and 2500-fold for DWV NS, *P*<0.0001 in both cases) because significant amplification of viruses bearing VDV-1 CP- (by 27-fold [*P* = 0.0217]) and DWV NS-regions (10-fold [*P* = 0.0314]) also occurred in the NV group relative to the control group C (Figure 4B). The dramatic rise of the recombinant genome(s) containing VDV-1 CP and DWV NS in the VH group was also accompanied by a statistically significant 30-fold decrease (*P* = 0.0151) of the DWV-type CP and 26-fold decrease (*P* = 0.0477) of the VDV-1 NS compared with the NV group. The levels of VDV-1 CP and DWV NS coding regions showed strong positive correlation (*r* = 0.9691) suggesting that this particular recombinant was preferentially acquired or amplified in the VH group pupae. The *Varroa*-exposed VL group also potentially acquired DWV from the mite as well as during larval feeding. It was therefore interesting to note that, when compared to the NV group, there was a statistically significant 54-fold decrease of DWV CP- (*P* = 0.0057) and a 36-fold decrease of VDV-1 NS-regions (*P* = 0.0151) in the VL group. Since both the VL and VH group pupae were mite-exposed but contained distinct levels and populations of DWV-like viruses we also characterised the viruses, and evidence of their replication, in the associated mites to determine if there was a correlation between high levels of virus in the honeybee and replication in the mite, as previously reported [38].

DWV- or VDV-1 specific qRT-PCR analysis demonstrated only a weak correlation with virus levels in the corresponding honeybee pupae (Figure S5). The highest correlations were found for the total load of DWV-like viruses determined using universal NS primers across the VL and VH groups (*r* = 0.567). Notably, we found that correlation between the levels of VDV-1 CP- and DWV NS-regions (sequences present in the predominant virus population in *Varroa*-infested VH group pupae; Figure S5) in the *Varroa* mites and the bee pupae were lower, *r* = 0.403 and *r* = 0.465, respectively. We went on to investigate whether we could distinguish between the mite-associated viruses in the VL and VH groups on the basis of their ability to replicate (as determined by negative strand synthesis) in the ectoparasite. Negative strand RNA was generally low but detectable in 10/15 mites analysed, with no significant difference between the DWV or VDV-1 CP levels (Figure S6). Together, these observations suggest that the low levels of DWV-like viruses in the VL group pupae cannot be explained by corresponding low levels of the virus in the mite and, similarly, that higher levels of the recombinant virus genomes in the mite-exposed honeybees (VH group) could not be attributed to either the preferential replication or absolute levels of these viruses in the associated mites.

### Virus diversity is markedly reduced in pupae but not in the associated *Varroa* mites

**Figure 5:**
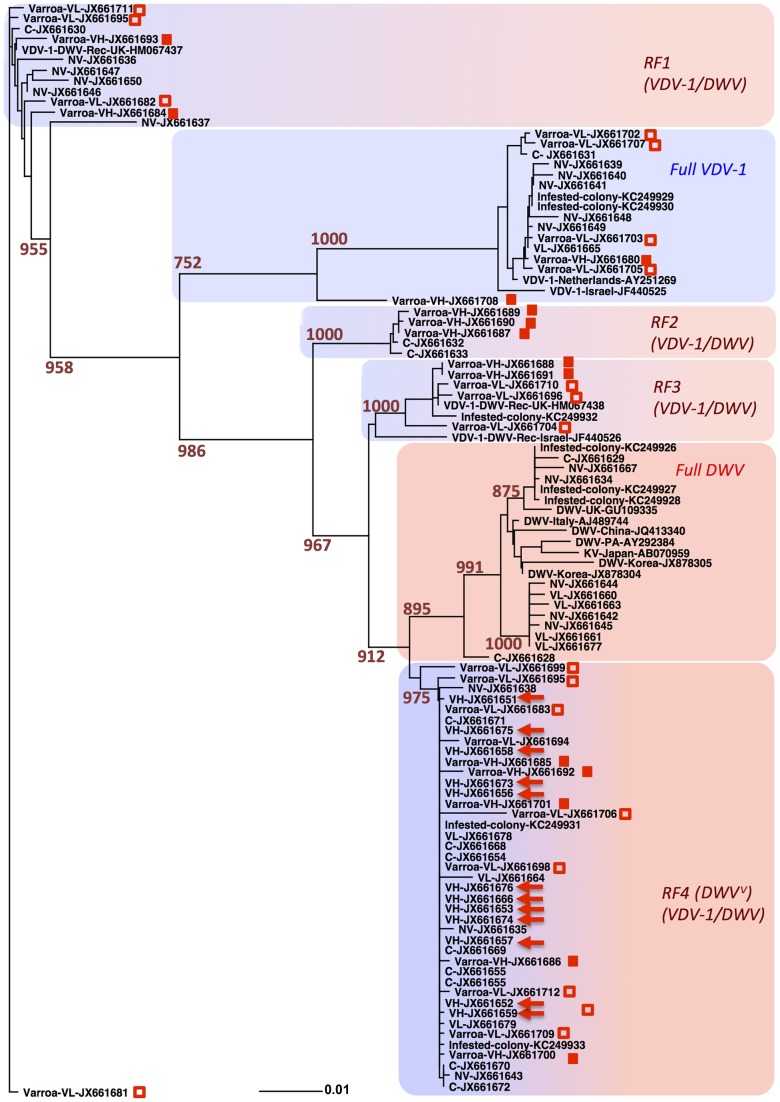
Phylogenetic analysis of the central region of DWV-like virus genome. PCR amplified cDNA was cloned and sequenced through the region corresponding to positions 4926 to 6255 of the DWV genome (GenBank Accession number AJ489744). The tip labels include GenBank accession numbers and are prefixed as follows: C, NV, VL, VH denote the corresponding honeybee pupae treatment group; Varroa-VH and Varroa-VL indicate sequences from *Varroa* mites associated with groups VH and VL respectively; “Infested-colony” denotes sequences derived from pupae from the *Varroa* source colony; DWV, VDV-1, VDV-1-DWV-Rec followed by a place name indicate reference DWV, VDV-1 and VDV-1-DWV recombinant sequences present in GenBank. Sequences derived from the group VH honeybee pupae are highlighted with arrows and sequences from *Varroa* mites associated with groups VH and VL are indicated with filled or empty squares respectively. Alignments were performed using CLUSTAL [77], and the neighbour-joining trees were produced and bootstrapped using the PHYLIP package [78]. Numbers at the nodes represent bootstrap values obtained from 1000 replications shown for the major branches supported by more than 750 replications. The length of branches is proportional to the number of changes. RF1 to RF4 indicate the distinct DWV/VDV-1 recombinant forms as defined by similarity to reference DWV and VDV-1 sequences (GenBank Accession numbers GU109335 and AY251269 respectively) in the CP and NS regions of the sequence., DWV^V^ indicates virulent form of DWV.

The dominance of recombinant viruses bearing VDV-1 CP and DWV NS coding regions in the VH group was strongly suggested by qRT-PCR (Figure 4B). Since recent studies have demonstrated that mite infestation is associated with a marked reduction in virus diversity at the regional scale [20], we extended our analysis to determine DWV-like virus diversity in individual pupae of the four exposure groups and, where appropriate, the co-isolated mites. In parallel, we also sampled random purple-eye stage pupae from the *Varroa*-infested colony to determine the pre-existing virus population at frame transfer. Nested PCR using generic (outer) and four specific (inner) primer pairs (Table S1) – for each possible combination of CP and NS region – was used to amplify a 1.3 kb fragment spanning a central region of the virus genome (corresponding to nucleotides 4926–6255 of the DWV genome; GenBank accession No. AJ489744) containing both CP and NS coding regions. We noted that no recombinants bearing a DWV CP region and VDV-1 NS region were detected in any of the experimental groups. For each of the eight pupae from the four exposure groups (C, NV, VL, VH), and pupae-associated individual mites from the VL and VH groups, PCR fragments were cloned and 8–18 individual clones sequenced. In total, 93 individual sequences were obtained of the 1330 nt. region and aligned with 12 DWV-like sequences (DWV, VDV-1, KV and recombinants thereof; see Materials and Methods) to generate a robust phylogenetic tree (Figure 5) due to the 22.71% sequence divergence in the region analysed.

The resulting dendrogram contained six distinct clusters, one each for non-recombinant DWV- or VDV-1-like sequences, together with four different VDV-1/DWV recombinant forms (designated RF1–RF4; Figure 5). Individual sequences obtained from pupae in exposure groups C, NV, VL and the *Varroa*-infested colony were present in all the major clusters indicating that these contain a significant diversity of viruses. In striking contrast, viral sequences from the VH experimental group exhibited almost no sequence divergence (0.15% at the nucleotide level), and consequently all clustered within a single clade (designated VDV-1/DWV RF4 in Figure 5). Therefore, the reduction in viral diversity (as previously determined by high resolution melting analysis) associated with the introduction of *Varroa* observed at the scale of tens of colonies exposed to the mite over several years [20] is reflected at the level of individual honeybee pupae following exposure to *Varroa* for 6 days.

One interpretation of the near-clonality of viral sequences in the VH group was that these were the only ones carried, and hence transmitted, by the mite. However, with the exception of the non-recombinant DWV cluster, which was not detected in the mite, the 32 viral sequences obtained from *Varroa* were widely distributed within the dendrogram (open symbols in Figure 5). These results imply that, with the possible exception of non-recombinant DWV, *Varroa* is capable of acquiring and maintaining a diversity of DWV-like viruses, but that – either during or following transmission to naïve pupae – only a subset of these (RF4 in Figure 5) are amplified to the very high levels observed in the VH group. Since the obvious difference between the horizontal transmission of DWV *per os* (larval feeding) and by *Varroa* is that the latter involves direct inoculation of virus to the haemolymph in pupae we investigated the recapitulation of this process by direct injection of pupae *in vitro*.

### Inoculation of honeybees by injection into haemolymph results in preferential amplification of specific VDV-1/DWV recombinants

**Figure 6:**
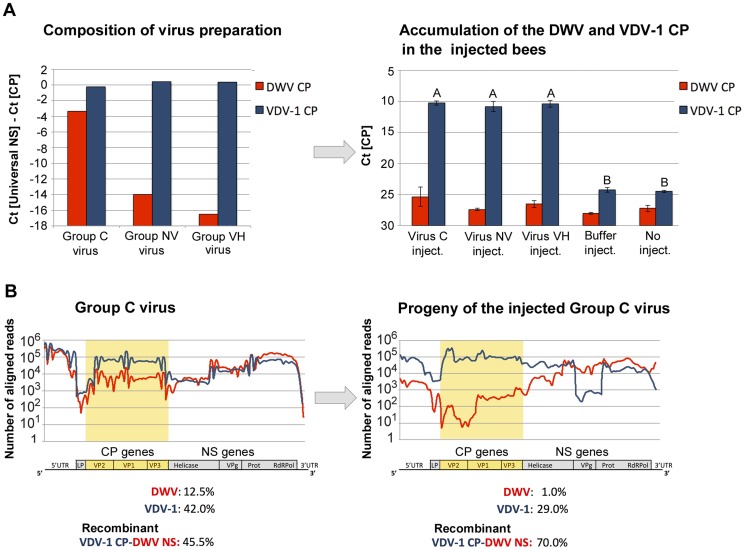
Changes in the strain composition of DWV complexes in honeybee pupae following direct injection of virus. Levels of the DWV- and VDV-1 CP-coding RNA determined by qRT-PCR (left panel) in the virus preparations used for injection, and (right panel) in pupae following incubation for 3 days. (A) Left panel: ΔCt values for the DWV-type and VDV-1-type CP were obtained by subtracting Ct values for the corresponding CP from Ct for the total DWV-like viruses quantified using “Universal” primers to the NS gene. Right panel: Ct values for the DWV-type and VDV-1-type CP. Six pupae were used for each virus-injected group, three pupae were used for each of the buffer-injected and non-injected control groups. Bars show mean value with standard error. Letters above the bars represent statistically significant groupings according to pairwise *t*-test comparisons for VDV-1 CP (*p*-value <0.01). (B) High-throughput sequencing of the virus preparations from the honeybees of group C (left), and the virus accumulated in the pupae injected with 20 ng of the virus preparation (right), 3 days post injection. The graphs show depth of coverage at genomic loci in DWV (red) and VDV-1 (blue) determined by high-throughput sequencing of viral RNA aligning to the DWV and VDV-1 sequences (GenBank Accession numbers GU109335 and AY251269 respectively). Only reads unambiguously aligning to DWV or VDV-1 sequences were used, with up to 3 mismatches tolerated in the 18 nt. seed region. The percentages of DWV, VDV-1 and the DWV-VDV-1 recombinants predicted by MosaicSolver [40] are shown below. The pileup graphs for DWV and VDV-1 are shown, respectively, in red and dark blue. The CP-coding region of the virus C preparation and the virus C-injected pupae, which shows a decrease of DWV coverage compared to the injected virus, is highlighted in yellow.

We directly injected white eye pupae (day 12–13 of development) maintained *in vitro* (as described in [39]) with virus particles purified from groups C, NV and VH pupae as described previously [15]. As before, we determined the proportion of the DWV- and VDV-1-type CP coding regions in the inocula and injected pupae (following incubation to the purple-eye stage for 3 days) by qRT-PCR using strain-specific primers to the CP and universal primers to the NS region. Virus preparations from groups NV and VH contained higher and broadly similar levels of VDV-1-like CP coding regions. The amount of DWV-like CP coding regions was much higher in the virus preparation from the group C pupae (where it accounted for ∼12% of the population) than from either the NV or VH group pupae (Figure 6A). Pupae inoculated with buffer alone exhibited no significant increased accumulation of DWV-like viruses when compared with untreated pupae (Figure 6A). In striking contrast, irrespective of the source of viral inocula, pupae directly injected with virus preparations exhibited high virus levels characterised by markedly amplified VDV-1-like CP coding regions when compared to DWV-like CP sequences (Figure 6A). Directly injected pupae were therefore similar, in both DWV-like virus levels and identity, to those previously observed in the VH experimental group (Figure 4A and Figure 1).

We additionally conducted next generation sequencing (Illumina paired-end reads) to comprehensively characterise the group C inocula and the viruses present in pupae injected with the group C virus. The composition of the inoculum, as determined by qRT-PCR and subsequent MosaicSolver [40] analysis of the NGS reads, were in close agreement and consisted of 12.5% DWV (in excellent agreement with the qRT-PCR-determined level, see above), 42% VDV-1 with the remainder being VDV-1 CP-encoding recombinants with a DWV-like NS region (Figure 6B). Three days after injection, the pupae inoculated with group C virus exhibited a marked reduction in the DWV content (from >12% to 1%) and a concomitant increase in recombinant forms of the virus (70% of the total) that were characterised by the presence of VDV-1 CP coding region and DWV-like NS regions (Figure 6B). These results further support our previous conclusion that DWV-like viruses bearing VDV-1 CP coding regions, and particularly recombinant forms with DWV-derived NS coding regions [15], have a selective advantage in *Varroa*-infested honeybee colonies, and additionally indicate that this advantage is manifest after transmission of the virus by direct inoculation and is not dependent upon *Varroa per se*.

### Independent verification of DWV diversity reduction by deep sequencing of the honeybees from a *Varroa*-infested colony

The sequence analysis of DWV in *Varroa*-exposed pupae (Figure 5) in the frame-transfer study supported the presence of a single, near-clonal, recombinant form of the virus in VH group honeybees. To formally exclude a role for PCR-biased amplification in this result and to extend our analysis to investigate virus diversity in independent samples (geographically and temporally), including asymptomatic and symptomatic newly emerged workers, we investigated virus diversity using next generation sequencing (NGS). We sampled individual adult nurse worker bees, both asymptomatic and exhibiting the obvious wing deformities and abdominal stunting characteristic of DWV disease, from a naturally *Varroa* infested colony. We additionally investigated virus diversity in purple-eye stage pupae to which we had injected (at the white-eyed stage 3 days previously) virus purified from pupae from the same colony a month earlier. Analysis was conducted on individual pupae using a high-throughput RNA-seq approach [41] with an mRNA protocol which allowed unbiased detection and quantification of all poly(A) containing RNA, this would include both host mRNA and the polyadenylated DWV-like genomic RNA [12].

**Figure 7:**
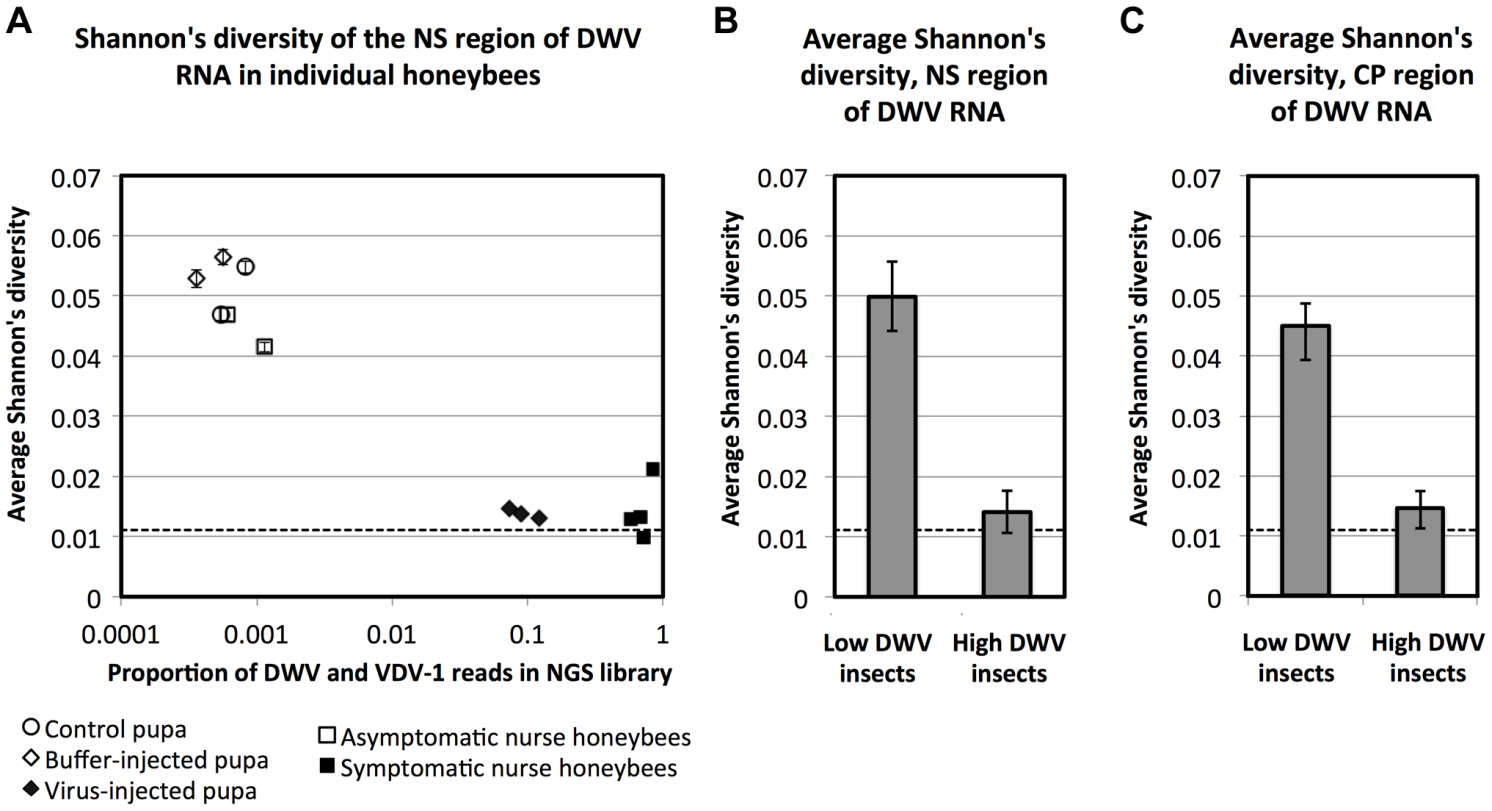
DWV diversity and the level of DWV accumulation. (A) Average Shannon's diversity Index (corrected for NGS sequencing error, as described in Text S1 and (Wood *et al.*, unpublished data) across the NS region, plotted against the proportion of DWV and VDV-1 reads. The error bar associated with each point is a 95% confidence interval for averages produced in this way. (B, C) Shannon's diversity index for all honeybees with low virus levels (groups “Control pupae”, “Buffer-injected pupae” and “Asymptomatic nurse honeybees”) and for the honeybees with high virus levels (groups “Virus-injected pupae” and “Symptomatic nurse honeybees”), (B) for the NS region and (C) for the CP region positions in the DWV reference genome, GenBank Accession number AJ489744, are 5008 to 9826 and 1751 to 4595 respectively. A 95% confidence interval for clonal input RNA library is shown as dashed line at 0.012. The sets of diversity values in (B) and (C) are significantly different, Least Significant Difference (LSD) test at 0.1%.

The NGS reads were aligned to reference DWV and VDV-1 sequences (GenBank Accession numbers GU109335 and AY251269 respectively), and the pileup profiles were analysed. The proportions of DWV and VDV-1 reads in the libraries (each containing about 10 million reads) showed a bimodal distribution and were either very high (from 7.41% to 83.87%, Figure 7A horizontal axis) for injected pupae and symptomatic nurse bees, or about a thousand fold lower (0.04% to 0.11%) for *Varroa*-naïve control pupae, for pupae inoculated with buffer alone and for asymptomatic nurse bees from the *Varroa*-infested colony (Figure 7A, Table S7). The remaining reads were of honeybee mRNAs. Distribution of the reads with similarity to DWV and VDV-1 suggested that all samples, irrespective of viral load, contained recombinant viruses with the CP derived form VDV-1 and NS region derived from DWV, as described above and in previous studies [15], [16]. To assess virus diversity, we calculated Shannon's diversity index [42] for the aligned NGS reads from each experimental pupa or adult bee. Despite the ubiquitous presence of recombinant DWV-like genomes (all consisting of a VDV-1 capsid and DWV non-structural coding regions) there was a striking reduction of virus diversity in the bees and pupae exhibiting high virus loads (Figure 7A,B,C). Average Shannon's diversity index for the NS and CP regions of the viral genomic RNA were significantly higher in the samples tested with low virus levels compared to those with high virus levels (0.1% level Fishers LSD test). At the same time, we observed no significant differences in Shannon's diversity index for NS and CP regions at the 5% level (Fisher LSD test) within the low virus group which consisted of *Varroa*-naïve control pupae, pupae injected with buffer alone, and asymptomatic nurse bees from a *Varroa*-infested colony (Figure S7 A, B). For the samples tested with high virus levels (pupae injected with virus *in vitro* and symptomatic nurse bees from a *Varroa*-infested colony), no differences were observed at the 5% level (Fisher LSD test) for the NS region. Indeed, combined low virus level and high virus level groups showed significant differences in Shannon's diversity index values for the CP and NS regions even at the 0.1% level (Figure 7B,C). For comparison we determined Shannon's diversity index for a sample prepared by *in vitro* transcription of two full-length DWV cDNA clones, GenBank accession numbers HM067437 and HM067438 [15], mixed, post transcription, at a known ratio and used as a template for NGS. We additionally used this control sample to determine the component of the observed diversity that was attributable to NGS sequencing errors which we quantified at about 0.5%, similar to that previously reported [43]. We calculated the threshold Shannon's diversity, a 95% confidence limit for clonal input RNA library (shown as dashed line in Figure 7A) using the approach described in Text S1 and (Wood *et al.*, unpublished data). Remarkably, while the diversity index of all samples with low DWV levels (control and buffer-injected pupae, and asymptomatic nurse honeybees) were well above this threshold, diversity values of samples with high DWV levels (virus-injected pupae and symptomatic nurse honeybees) were either very close or below this clonality threshold. Similar results were obtained when diversity was estimated using multiple sampling as described in Material and Methods (Figure S7). In this case the clonality threshold value (the range shown with the dotted lines in Figure S7) was also almost indistinguishable from the diversity present in symptomatic nurse bees from the *Varroa*-infested colony indicating that the diversity in these honeybee samples was close to the limit of detection using NGS analysis. This reinforces the near-clonal nature of the virus population in *Varroa*-exposed symptomatic nurse bees and is in good agreement with the sequence analysis of VH group pupae following PCR amplification of the central region of the virus genome (Figure 5). To further explore the near-clonal nature of the virus population in symptomatic nurse bees from a *Varroa*-infested colony we used a pair of flanking primers to the DWV-like genomic RNA to amplify and clone full-length viral cDNAs from these samples (GenBank accession number KJ437447). The central 1330 nt. region of this clone was identical to that previously characterised from VH group pupae (Figure 5) despite being sampled from a separate colony in a different apiary over two years later. The consensus viral sequences, which were assembled from the NGS libraries from symptomatic honeybees with high DWV levels, showed highest overall identity with the full-length clone KJ437447 (specifically 99.15% [SD = 0.31%] nucleotide and 99.78% [SD = 0.09%] amino acid identity) and the 1330 nt. sequences from VH group pupae, *e.g.* JX661656 (98.84% [SD = 0.57%] nucleotide and 100.00% amino acid identity; Table S7). In respect to the samples with low DWV levels, we found that Shannon's diversity index for the NV group sequences (*Varroa*-free orally infected pupae; Figure 5) was 0.04172. This value was very close to the Shannon's index values in the same genomic region for the pupae exhibiting low virus levels from the *Varroa*-infested hive, 0.03623, SD = 0.00026, for control (*i.e.* not injected) pupae (0.03929, SD = 0.00097) and for pupae injected with buffer alone (0.03929, SD = 0.00097); Figure S7 C).

## Discussion

The introduction and global spread of the parasitic mite *Varroa destructor* in honeybees (*Apis mellifera*) has had significant impact on the health and survival of infested colonies [8], [13]. Colony losses associated with *Varroa* are predominantly attributed to the RNA virus payload vectored by the parasite and transmitted when the mite feeds on honeybee haemolymph [10], [44]. Although *Varroa* were reported to vector at least 5 RNA viruses, the picorna-like Iflavirus deformed wing virus (DWV) is of particular interest and importance; deformed wing disease is associated with mite infestation [9] and high levels of DWV exacerbate overwintering colony losses [10]. Furthermore, of the viruses analysed, only DWV levels increased upon introduction of *Varroa* to Hawaii [20]. Here we show that virus levels exhibited a bimodal distribution in developing honeybee pupae – low in the absence but generally high following mite exposure – with high viral levels associated with emergence of a distinct recombinant form of the virus. This complements observations of the effects of the introduction of *Varroa* at the regional scale (*e.g.* Hawaii), which is associated with a dramatic reduction in DWV variation and the emergence of dominant strains over a scale of months or years [20]. Our study also strongly suggests that DWV is widespread and present in all UK honeybees, in *Varroa*-free and *Varroa*-infested colonies. By using different molecular approaches including RNA-seq, cloned cDNA sequencing and qRT-PCR, we demonstrated that, contrary to the recently reported figure of 36% DWV presence in the UK honeybees [45], DWV was present in all *n* = 250 tested honeybees, including those from a *Varroa*-free region (*n* = 47) (Figure S1 and data not shown), in line with previous studies from our group [15] and other [46]. Striking differences in reported DWV incidence might be due to differential sensitivity of the primers used in these studies, especially in the honeybees with low DWV levels, which have higher genetic diversity (Figures 5, 7).

To better understand the interactions of the honeybee host, *Varroa* and DWV that account for the observed emergence of presumed pathogenic, near-clonal strains of the virus we analysed events at the level of individual honeybee pupae. We reasoned that the large scale global, temporal and population changes observed reflect the cumulative outcome of interactions that occur at the level of individual pupae within the colony. We further reasoned that analysis at this level would allow two hypotheses accounting for the high levels of overt deformed wing disease beekeepers associate with heavy *Varroa* infestation to be tested. These hypotheses, themselves not mutually exclusive, are that; a) *Varroa* suppresses the antiviral honeybee defences so allowing unrestricted DWV replication and b) the presence of *Varroa* results in the selection and transmission of particular pathogenic variants of DWV, resulting in serial amplification within a mite infested colony. We tested these hypotheses by transferring honeybee larvae from a *Varroa*-free to an experimental *Varroa*-infested environment, stratified pupae of a standardised age in terms of mite-exposure and viral load, and investigated transcriptome and RNAi responses of the host and the virus population in individual mites and associated pupae.

We demonstrated that *Varroa*-naïve honeybee larvae (group C) transferred into a *Varroa*-infested colony (effectively mimicking the exposure to oral and mite-transmitted DWV during larval and pupal development) can, after incubation for 6 days, be stratified into three distinct pupal groups by *Varroa* exposure (presence or absence) and DWV level (high or low). High virus levels were not observed in the absence of *Varroa*. Group NV comprised pupae from capped cells free of *Varroa*. As *Varroa* enters the cell immediately prior to capping [27], we assume this group only acquired viruses *per os* during larval feeding. The VH and VL groups contained *Varroa* within the capped cell with evidence of *Varroa* feeding on the pupae – including nymphal forms present and signs of abdominal piercing. These groups of pupae harbour strikingly different DWV populations: the VL group having low viral levels and high diversity that are not significantly different from the C and NV groups, whereas the VH group carries 1,000–10,000 times the viral loads of a single phylogenetic type (Figure 5). We compared the transcriptome and virus-specific siRNA pool between the four exposure groups, the virus level and diversity in associated mites and determined the consequences of direct virus injection to experimentally test the two proposed hypotheses accounting for the observed dominance of particular virulent strains of DWV in the presence of *Varroa*. We additionally characterised virus sequence and diversity in injected pupae and independent naturally *Varroa*-infested colonies.

### Transcriptome changes in response to DWV and *Varroa* mite feeding

Previous analyses of transcriptome or immune response changes in *Varroa*-exposed honeybees have produced contradictory results, perhaps due to the analysis of pooled individuals and/or pupae of different ages. These confounding influences may have obscured the marked changes in gene expression that we observed in response to either mite or viral pathogens, as emphasised by the transcriptome differences observed in the *Varroa*-associated pupae in groups VL and VH, which respectively exhibited 493 (∼5% of transcriptome) and 951 (∼9%) significantly differentially expressed (DE) genes when compared with the control group C (Figure 2A). By stratifying *Varroa*-exposed pupae by viral load we can provisionally define transcriptome changes resulting from mite-associated activities such as wounding, feeding and exposure to salivary peptides (the 444 genes shared by VL and VH groups) and those triggered by the high viral load (>3 log_10_ higher in VH than VL; Figure 2A). We acknowledge that the C to VL contrast may include genes involved in suppressing high levels of mite-transmitted DWV accumulation, an interpretation that warrants further study. The NV, VL and VH pupal groups also acquired DWV during larval feeding in the *Varroa*-infested colony, which on account of the preferential amplification of particular recombinant forms of DWV (Figure 4B, discussed further below and [15]) contains a distinct virus population, the composition of which differs from historically *Varroa*-free control colonies. Transcriptome comparison between the C and NV groups showed significant changes in a large number of genes (416, ∼4% of transcriptome), many of which were also altered in the C to VL (220) and C to VH (385) contrasts (Figure 2A, B). These may reflect *per os* exposure, and the resulting responses to the particular virus population circulating in the *Varroa*-infested hive, which resulted in changes of DWV strain composition in NV compared to C (Figure 4B), together with changes resulting from environmental differences (such as circulating pheromone) between the originating and test colonies which would be common to all three exposure groups. The set of 59 genes DE in the contrast NV to VL (exposure to *Varroa* feeding at the pupal stage which did not result in elevated viral loads), was largely different from the DE genes in contrast C to NV with only one gene shared. At the same time, the NV to VL set showed high commonality with the DE genes in the contrasts C to VH, C to VL, and NV to VH (34, 51, and 27 genes respectively; Figure 2A, B). Observed DE gene commonality in the contrasts was consistent with an orthogonal expression pattern (Figure 2B) following treatments (Figure 1), with different sets of genes DE in response to *per os* infection (C to NV), exposure to mite feeding (NV to VL), and high DWV (VL to VH) (Figure 2B).

Gene Ontology (GO) analysis gave additional insights into the transcriptional responses in honeybees following experimental treatments (Figure 1). It has been demonstrated that genes associated with the same GO terms are likely to have the same transcription factors binding to their promoter regions, which may result in co-regulated expression of these gene sets [47]. Therefore, statistically significant overrepresentation of GO BP terms associated with the DE genes may suggest coordinated and distinct honeybee responses to *per os* exposure (C to NV contrast) and to *Varroa* and/or *Varroa*-injected virus (NV to VL contrast) (Figure 4A, Figure S1). Such coordinated transcriptional responses may include genes involved in suppression of virus replication in the NV and VL group pupae (Figure S1), which will require further analysis. In contrast to this situation, the transcriptional changes specifically associated with the increased virus levels in group VH (VL to VH contrast) had no significantly over- or under-represented GO BP terms (Figure 2A, Table S4). This suggests that honeybees did not respond in a coordinated manner to the increased virus load, and that presumably unrestricted DWV replication caused dysregulation of transcription and/or mRNA stability in the honeybee similar to that previously reported in picornavirus infection of mammalian cells [48]–[50].

### Changes in expression of immune-related genes

We analysed changes in expression of known and presumed immune-related genes (Table 1, Figure S4) defined previously [34], [35] and by gene ontology (GO) terms associated with *Drosophila* homologs [33]. In particular, a number of proposed components of the Toll signalling pathway were affected, although the lack of activation of the antimicrobial peptide genes suggested that no activation of the Toll and Imd pathways had occurred [34], [35], [51]. In contrast to both the *Varroa*-exposed groups (VL and VH) the NV group was the only group in which there were more up- than down-regulated immune-related genes when compared with the control (Table 1, Figure S4). The majority of the changes seen in the C to NV contrast were also seen in the groups that acquired DWV both orally and via *Varroa* (C to VH, C to VL contrasts), implying that *Varroa* exposure may exert a dominant immunosuppressive influence over any up-regulation observed following oral exposure alone. Significantly enhanced expression of the honeybee orthologue of Vago (GB10896; Table 1), a secreted protein upregulated in *Drosophila* and *Aedes* following detection of viral dsRNA by Dicer during virus infection [52], [53], was observed in all groups exposed to oral DWV in the *Varroa*-infested colony (NV, VL and VH) when compared with group C.

*Varroa* exposure (VL or VH groups) resulted in down-regulation of several putative components of the honeybee Toll signalling pathway [51], including two Toll receptor orthologs (GB10640, GB17781), CLIP-domain protease spirit (GB14044) and the Toll receptor ligand spatzle (GB15688). In addition, spatzle was down-regulated when the VH group was compared against the other experimental groups, suggesting down-regulation of this gene may be a response to the elevated virus levels in the group VH, rather than the presence of *Varroa per se*. Toll signaling pathways are implicated in antiviral resistance to the RNA virus Drosophila virus X [54], possibly controlling proliferation of haemocytes which, because of their involvement in phagocytosis, play a central role in insect immunity [54], [55]. We also observed down-regulation of a Tetraspanin 68C (Tsp68C) ortholog (GB16002, GB13670), a cell surface membrane scaffolding protein previously implicated in receptor modulation during hemopoiesis [56], an ortholog of *pannier* (GB19895), a GATA transcription factor required for differentiation of plasmatocytes (which resemble the mammalian macrophage lineage [57]), and a *serrate* ortholog (GB15155), a membrane ligand for the Notch receptor implicated in differentiation of haemocyte-related crystal cell precursors which function in pathogen defence via melanisation [58]. These transcriptome changes may help explain functional studies in which salivary secretions from *Varroa* mites damage moth caterpillar haemocytes [59] and suggest that *Varroa*-mediated depletion of haemocytes, a key component of the immune response of insects [60]–[62], may contribute to enhanced susceptibility to DWV and other viruses. Interestingly, we also observed suppression of the Friends-of-GATA transcription factor U-shaped (*ush*) ortholog (GB16457), in the C to VL and NV to VL contrasts. *Drosophila ush* is reported to antagonise crystal cell development [63], [64], implying that the low level of virus accumulation in the VL group may be due to elevated numbers of crystal cells resulting from *ush* down-regulation.

Although by definition descriptive, transcriptome analysis of pupae stratified according to *Varroa* and virus-exposure, also provides direct insights into possible pathogenic mechanisms. In the contrast C to VH we observed differential expression of orthologs of five *Drosophila* homeobox genes (summarised in Table S8) encoding transcription factors which are involved in insect development [65]. Most of these DE genes are reported to be expressed at early pupal stages and involved in abdomen (Abdominal B), appendage (apterous) or brain development (extradenticle). This may explain previously reported developmental abnormalities in the honeybee that are associated with high DWV levels at the pupal stage [13] and warrant further investigation to potentially determine the molecular mechanism underlying DWV pathogenesis.

### RNAi responses to DWV in *Varroa*-infested pupae

Notwithstanding the absence of significant changes in gene expression of key components, such as Dicer and Argonaute, of the RNAi response – the major antiviral mechanism in insects [25] – we explored the relationship between DWV-like virus levels and the corresponding siRNA populations. In particular, we sought to investigate if high levels of DWV in VH honeybees was associated with the limited accumulation of virus-derived siRNA, implying the virus may express an siRNA suppressor as, for example, demonstrated in Alphavirus infection of mosquitos [66]. Although DWV- and VDV-1 specific siRNAs were recently detected in adult honeybees [67], [68], these studies could not show if RNAi is involved in suppression of the virus, because viral genomic RNA levels were not quantified. Analysis of siRNAs in the honeybees of the frame transfer experiment showed that the predominant DWV- and VDV-1-specific siRNAs were 22 nt in length with genome sense strand-specific siRNAs present at a 3–4 fold excess over antisense. This was consistent both with the presence of replicating DWV-like viruses in all experimental groups and with the known activity of Dicer in other insects [25] and strongly suggests normal functioning of Dicer in honeybees [69]. As insects do not amplify siRNA populations [25], it was unsurprising that virus-specific siRNA levels were broadly proportional to the level of viral genomic RNA determined by qRT-PCR; the C and NV groups exhibited ∼10^3^ times less viral genomic RNA than the VH group and normalised siRNA levels differed by ∼770 times (Table S5). The exception to this was the siRNA response in the VL group which was ∼5 times higher proportionally than the level of VL virus genomic RNA (Table S5). The relationship between the levels and compositions of the viral genomic RNA and virus-derived siRNA may be altered by differences in targeting of the individual components of DWV-like virus population by the honeybee RNAi machinery, as observed during West Nile virus infection of mosquitos [70]. Although the presence of virus-specific siRNAs does not necessarily correlate with effective silencing – viruses may encode late-acting suppressors such as the Argonaute-inhibiting 1A protein of cricket paralysis virus [71] – the robust siRNA response in the VL group may contribute to suppression of DWV replication and the differences between this response and that observed in the VH group may be a fruitful area for further analysis.

### Genetic diversity of the DWV population is determined by route of transmission rather than preferential amplification of virus in *Varroa*

The introduction of the parasitic *Varroa* mite elevates the level of DWV-like viruses [20], amplifies particular strains that that are best defined as recombinant forms (RF) bearing the capsid determinants of VDV-1 and non-structural genome region from DWV [15], [16] and dramatically reduces the diversity of DWV-like viruses in a population [20]. Using complementary approaches including strain-specific qRT-PCR and sequencing together with next generation sequencing of the virus genome and host siRNA response to infection, we analysed individual pupae exposed to DWV during larval feeding and following mite exposure, and recapitulated horizontal transmission of virus by *Varroa* using direct injection.

The C, NV and VL exposure groups all carried low viral loads and exhibited high virus diversity (Figure 4B, Figure 5). However, the virus populations carried were distinct, with the NV and VL experimental groups containing a diverse range of recombinant forms of DWV-like viruses bearing the capsid coding region of VDV-1 and the non-structural coding regions of DWV. In contrast, the VH group exhibited very high levels of a specific near-clonal (0.15% divergence in the regions sequenced) recombinant form of DWV (labelled RF4 in Figure 5). Due to the subsequent identification of the same near-clonal virulent virus in temporally and spatially distinct samples (see below) we henceforth designate this virus DWV^V^ to discriminate it from other circulating recombinants forms. This suggests that the changes reported in virus levels and diversity at a regional scale [20] reflect events occurring within a few days (uncapped to the purple-eye stage) in individual mite-exposed pupae. Nearly identical, clustering tightly within the DWV^V^ clade, were also detected in pupae from the C, NV and VL groups (Figure 5). Since these groups have significantly lower viral loads it implies that the high viral loads seen in the VH group cannot be solely attributed to their infection with a particular recombinant form of the virus.

We reasoned that there were two possibilities that might account for the marked amplification of DWV^V^ in the VH group pupae. Firstly, the mite may have delivered a high dose of one specific recombinant form, perhaps reflecting its preferential replication in the ectoparasite. Secondly, we considered that DWV^V^ might have a growth advantage when inoculated into haemolymph by *Varroa* (potentially in addition to the preferential amplification in the mite). To distinguish between these possibilities we sequenced qRT-PCR amplified viral RNA from mites co-isolated from capped cells containing group VL and VH pupae. We also investigated the consequences of *Varroa*-independent mechanical virus transmission by direct injection of mixed virus preparations to *Varroa*-naïve pupae and subsequent monitoring of virus levels and diversity.

Individual *Varroa* mites contained a diversity of DWV-like sequences that were well distributed throughout the phylogenetic tree of virus sequences from pupae (square symbols, Figure 5). Using VDV-1- and/or DWV-specific primer pairs spanning the central 1.3 kb region of the virus genome mites were detected containing VDV-1 and all four distinct RFs identified in the four experimental groups in the frame transfer study. Only non-recombinant DWV was absent from the 32 mite-associated viruses sequenced. We also detected negative strand sequences of both DWV and VDV-1 CP regions in the majority of the 15 mites tested (Figure S6), implying that virus replication does occur in the mite. Although we did not detect DWV CP among the central 1.3 kb region sequences amplified from the mites (Figure 5), this could be a consequence of limited experimental sampling and the higher levels of VDV-1 CP in the population, a conclusion supported by analysis of the negative strands present (Figure S6).

Although further studies will be required to determine whether sampling stochasticity accounts for the apparent absence of non-recombinant DWV in *Varroa*, together these results suggest that – at least at the level of the entire mite – there is no selection, either by absolute presence or replication capability, for the DWV^V^ RF that accumulates to high levels after mite exposure in VH group pupae.

Since the diversity of virus present in *Varroa* indicates that the near-clonal virus population in the VH group is not due to the mite delivering either a restricted virus type or to elevated levels of DWV^V^ in the mite we went on to inoculate pupae with a mixed virus population prepared from group C pupae and characterised the resulting virus population after three days. Recipient purple-eye stage pupae contained high virus loads which, compared with the inocula, had markedly reduced levels of DWV-like virus and elevated levels of a VDV-1/DWV recombinant (Figure 6). Although, the resulting virus diversity was not as limited as seen in the naturally infected VH group, we attribye this to the restricted incubation time between inoculation and sampling (3 days *vs.* 6 days), in part imposed by experimental limitations of working with late-stage larvae and early-stage pupae which are vulnerable to handling damage. Despite these limitations, these results clearly demonstrate that direct inoculation of a mixed virus preparation, recapitulating virus inoculation by the mite, results in a marked reduction in virus diversity. We additionally demonstrated, by RNA-seq analysis of temporally and geographically independent symptomatic nurse bees and similarly independent pupae directly injected with virus preparations, that essentially the same near-clonal virus (DWV^V^) was present as previously identified in the VH group pupae. In parallel, control asymptomatic nurse bees or mock-injected pupae exhibited high diversity and low levels of virus (Figure 7, Figure S7, Table S7), as previously seen in the C and NV groups during the frame transfer study. The unselective RNA-seq methodology excludes the possibility that virus clonality at high virus loads was a consequence of PCR bias. The remarkable restriction in virus diversity in both injected pupae or symptomatic nurse bees exhibiting high viral loads was in good agreement with that seen in group VH pupae (Figure 5) determined following qRT-PCR amplification (0.15% diversity).

We propose that the strikingly elevated levels and associated restricted diversity of DWV^V^ (RF4-type; Figure 5) in both the *Varroa*-exposed VH group pupae and characteristically DWV symptomatic nurse bees is because this virus has a preferential advantage when delivered directly to haemolymph of developing pupae. There remains the possibility that DWV^V^ alone replicates to elevated levels in the salivary glands of *Varroa* and is the only DWV-like virus transmitted during feeding. However, we do not favour this hypothesis as we would expect it to result in DWV^V^ being the predominant virus detected when whole-mite RNA samples were analysed. Furthermore, we also present evidence (Figure 7A, Table S7) that DWV^V^ predominates when a mixed virus population is directly inoculated. Nevertheless, it remains an intriguing avenue for further study. Assuming this is not the explanation, the molecular mechanisms underpinning the advantage of the near-clonal DWV^V^, be it evasion of the host antiviral response, specific tissue tropism or some other aspect of the virus-host interaction, will require further studies. This will necessitate immunohistological analysis of orally infected or injected pupae, the development of a reverse genetic system to identify determinants of DWV tropism, and the analysis of the contribution of immune-related (and other) host genes using RNAi-based strategies [72], [73].

### Conclusions

Without proper management *Varroa* has a devastating effect on honeybee colony viability and consequent honey production and pollination services. We show here that the markedly elevated levels of DWV-like viruses in *Varroa*-exposed honeybee pupae are likely attributable to the direct inoculation of a specific virus, DWV^V^, by *Varroa* to haemolymph. Repeated cycles of *Varroa*-replication within an infested colony would preferentially amplify DWV^V^, potentially resulting in it becoming the predominant virus present, transferred both by *Varroa* and *per os*. Further studies will be required to determine whether such a virus, if sufficient were ingested, would also cause symptomatic infection. Oral susceptibility to a virulent form of DWV may also explain reported cases of deformed wing disease symptoms seen in *Varroa*-free colonies in Hawaii [20] and Scotland (Andrew Abrahams, pers. comm.), but may also reflect genetic variation and the presence of particularly susceptible pupae in the colony.

Our study demonstrates that a proportion of *Varroa*-exposed pupae (the VL group) do not exhibit elevated levels of the near-clonal DWV^V^ recombinant (Figure 5). Further *in vitro* studies will be required to determine whether these are naturally resistant – and therefore form the basis for genome wide association studies of the genetic determinants of virus resistance – or if they reflect the stochastic nature of the transmission event from the mite.

## Materials and Methods

### Honeybees, frame transfer experiment and sampling

This study was based around an experiment in which a brood frame containing newly hatched larvae from a *Varroa*-free colony was introduced into a *Varroa*-infested colony. The larvae were left to develop within the *Varroa*-infested colony, and pupae were collected 11 days later from capped brood cells at the purple eye stage and analysed using a range of molecular methods. The *Varroa*-free honeybee (*Apis mellifera*) colony with a naturally-mated one-year-old queen was imported from Colonsay, Scotland, an island with no historic reports of *Varroa* incidence and no imports of honeybees from *Varroa*-infested areas. This allowed us to exclude the presence of DWV strains associated with *Varroa* mite infestation. As a source of *Varroa* mites and the mite-associated DWV strains, we selected a Warwickshire honeybee colony, heavily infested with *Varroa* and having high DWV levels in honeybees and mites. The *Varroa*-free and *Varroa*-infested colonies were contained in separate mesh flight cages (dimensions: 6 meters long, 2.5 meters wide, 2 meters high) and maintained on an artificial diet of sugar syrup and pollen. The pollen was imported from *Varroa*-free Australia to exclude possible contamination with *Varroa*-associated viruses through foraged food and was pre-screened by PCR before use for DWV-like viruses. In order to minimise possible effects on honeybee gene expression due to the differences in nutrition, both the control *Varroa*-free and the *Varroa*-infested colonies were maintained in flight cages in the same apiary (at the University of Warwick, UK) and were fed on the artificial diet for two months before the start of the frame transfer experiments. Neither colony was treated with miticides.

The experimental infestation, summarised in Figure 1, was conducted on 4^th^–15^th^ August 2011. As stated previously, it involved the transfer of a brood frame, which contained newly hatched honeybee worker larvae (on day 4 of development), from the *Varroa*-free to the *Varroa*-infested colony. As a result, the transferred larvae were exposed to *Varroa*-selected DWV-like viruses in brood food delivered by the nurse honeybees of the *Varroa*-infested colony for five days before brood cell capping on day nine of development (Figure 1, Treatment 1, Oral DWV infection). Honeybee larvae were left to develop in the capped cells for six days and then were sampled as pupae on day 15, when they had reached the purple-eye stage of development [74]. A proportion of these brood cells were naturally infested with *Varroa* and hence contained pupae that were subjected to mite feeding (Figure 1, Treatment 2, Mite feeding). We sampled *Varroa*-infested pupae and the mites associated with individual pupae, with mite feeding confirmed by the presence of the mother mite and at least one protonymph [27]. Control pupae at the same developmental stage were sampled from the *Varroa*-free hive at the same time. A colony from a separate apiary in Warwickshire that exhibited *Varroa* mite infestation for over a year was sampled in August 2013 to assess the virus populations in colonies with established *Varroa* infestation.

The pupae and the *Varroa* mites associated with each infested pupa were individually snap frozen in liquid nitrogen immediately after being removed from brood cells and stored at −80°C prior to total RNA extraction. For total RNA extraction, whole individual honeybee pupae were ground to fine powder in liquid nitrogen, and half of the powder used for RNA extraction, carried out using 1 mL of Trizol Reagent (Invitrogen) according to the manufacturer's instructions. Total RNA extraction from *Varroa* mites was carried out using RNeasy spin columns (Qiagen RNeasy Plant Mini kit).

Virus purification from honeybee material and extraction of the viral genomic RNA from virus particles were carried out as described previously [15].

### Microarray transcriptional profiling and statistical analysis

For genome-wide analysis of the honeybee transcriptome total RNA preparations from eight individual honeybees from each of the four experimental groups (32 honeybees in total) were purified further using RNeasy Plant Mini kit spin columns (Qiagen). RNA concentration, purity and integrity were assessed using a 2100 Bioanalyzer and an RNA 6000 LabChip (Agilent Technologies). The probe preparation, hybridization and scanning were carried out according to the Agilent instructions, essentially as in [75]. Total RNA extracts from an individual honeybee were used to produce Cy3- and Cy5-labelled aRNA samples using a Low Input RNA fluorescent linear amplification kit (Agilent Technologies), according to the manufacturer's instructions. The Cy3-and Cy5-labelled samples were used in a two-colour dye-balanced loop design [30], [31] for a genome-wide analysis of the honeybee transcriptome with the custom expression oligonucleotide microarray. Four slides, each with eight two-channel arrays were employed, allowing two replicates per sample, one green and one red. Different treatment groups were allocated to the green and red channels in each array; the loop design ensured that each sample was indirectly compared with all other samples. The array, in 60K ×8 format, included 60 nt oligonucleotides specific to 10,157 transcripts of the *Apis mellifera* Official Gene Set 1, OGS1 [32]; the array also contained probes to all the honeybee RNA viruses known to date. Each probe was replicated five times to enable robust statistical analysis. Sequences of the probes of the honeybee whole genome expression microarray, a 60-mer oligonucleotidide array based on the *Apis mellifera* transcriptome (OGS1) and *Apis mellifera* fungal and viral pathogens (Agilent ID: 027104, SurePrint G3 Custom GE 8×60K), are available in the ArrayExpress database (www.ebi.ac.uk/arrayexpress) under accession number A-MEXP-2251. Following hybridisation, the microarrays were scanned using Agilent Technologies G2565CA Scanner and the fluorescence intensity data were processed using feature extraction software (Agilent Technologies). Cy3 and Cy5 fluorescence intensities for each spot were measured as values of green and red pixels respectively. The details of the array experiment design, sample description, and microarray data are available in the ArrayExpress database (www.ebi.ac.uk/arrayexpress) [76] under accession number E-MTAB-1285. One array failed (assigned to VL green and NV red) leaving 62 channels for final analysis.

For additional confirmation we conducted qRT-PCR analysis with primers specific to *Paenibacillus larvae ssp* and *Melissococcus plutonius* (Table S1), the causal agents of American foulbrood and European foulbrood, respectively, which showed that the samples were free of detectable levels of these bacterial pathogens.

The unprocessed intensity scanning values were both within-array and between-array normalized using the linear model based Limma R package [77]. Differentially expressed (DE) genes in all six possible contrasts were found using Limma (via function “lmscFit” incorporating intraspot correlation) and also the R GaGa package for gamma-gamma Bayesian hierarchical modeling [78]–[80]. A gene was considered as differentially expressed (DE) in a given contrast (using a t-statistic moderated across genes) when the average expression exceeded 6.0, the fold change exceeded 14%, the Limma analysis *p*-value adjusted for multiple genes was less than 0.05 and the posterior probability determined by GaGa was above 0.6. Microarray results were validated by qRT-PCR using a set of primers for certain honeybee genes and DWV (Table S1).

For Gene Ontology (GO) analysis a three-stage process was used. Genes in the latest *A. mellifera* genome annotation, Amel_4.0 (http://hymenopteragenome.org/beebase), corresponding to genes in *A. mellifera* OGS1 were found using protein blast. GO terms associated with Amel_4.0 genes were then obtained using Blast2GO [81] with the SwissProt database option. Finally, over- and under-represented GO terms in the sets of DE honeybee OGS1 genes in each contrast were obtained with BiNGO, using a hypergeometric test, a Benjamini and Hochberg FDR correction and a significance level of 0.05 [82].

For Principal Component Analysis (PCA), the significant DE genes in all six contrasts were pooled and ranked by their adjusted *p*-value. The 60 with the lowest adjusted *p*-value were selected, all of which appeared in the contrast C to VH; the other contrasts' contributions were 35 (C to VL), 21 (NV to VH), 19 (C to NV), 4 (NV to VL) and 11 (VL to VH) genes. Principal components of the expression profiles across the 62 microarray channels were found and (the first two) plotted using the princomp and biplot functions in R [83].

### Next generation sequencing of small RNA libraries

For high throughput sequencing of small RNA, we pooled equal amounts of the Trizol-extracted total RNA from individual honeybees and isolated the 15 to 40 nt RNA fraction, which was separated using denaturing polyacrylamide gel. The RNA pools were ligated to the oligonucleotide adapters, reverse-transcribed and amplified using the TruSeq Small RNA Sample Prep Kit (Illumina small RNA kit). The libraries were sequenced using the Illumina HighSeq 2000 platform, producing 15–25 million reads per libraries (GATC-Biotech, Germany). The small RNA NGS sequencing data are available in the ArrayExpress database (www.ebi.ac.uk/arrayexpress) [76] under accession number E-MTAB-1671. The reads were cropped to remove adapter sequences and aligned to reference viral and miRNA sequences using Bowtie [37]. Samtools mpileup was used to produce the siRNA and miRNA coverage profiles.

### Characterization of viral RNA

Real-time reverse transcription PCR was carried out essentially as in [15]. In brief, RNA extracts were treated with DNAse, then purified DNA-free total RNA preparations were used as a template to produce cDNA using random primer and Superscript III reverse transcriptase (Invitrogen). The cDNA samples produced were used for real-time PCR quantification of the DWV or host transcripts using SYBR green mix (Agilent Technologies). Oligonucleotide primers are summarized in Table S1.

For strand-specific quantification of viral RNA of DWV and VDV-1 types reverse transcription was carried out at 50°C using Superscript III reverse transcriptase (Invitrogen) and the tagged primers designed to anneal to the negative strands RNA of DWV or VDV-1, primers 389 and 391 respectively (Table S1). The qPCR step was carried out using corresponding DWV or VDV-1 specific primers in negative polarity (Table S1, Primers 1384 or 1382) and primer 388 identical to the sequence of the tag (Table S1, primer 388).

Amplification of the cDNA fragments corresponding to the central region of DWV genomic RNA was carried out by nested PCR using GoTaq PCR mix (Promega) and primers 155 and 156 (Table S1) using the cDNA extracted from the honeybees and the mites, pooled according to their treatment groups. The outside PCR primers were designed to amplify all known DWV-like sequences. For each first round reaction we carried four second round amplification reactions using VDV-1- or DWV-specific primers, 151–154 (Table S1), which allowed distinction of VDV-1-type and DWV-type CP and NS regions, thereby enabling amplification of all potential combinations, even those present at very low levels. The PCR fragments were cloned into pGemT-Easy (Promega) and sequenced using the Sanger dideoxy method. GenBank accession numbers for the reported sequences are JX661628–JX661712 and KC249926–KC249933. The full-length cDNA of DWV, GenBank accession number KJ437447, was amplified by RT-PCR using primers specific to the published termini of DWV and VDV-1 RNA and cloned into the pCR-TOPO-XL vector (Invitrogen) as described in [15]. The sequences were aligned using CLUSTAL X [84], and phylogenetic analysis of the sequences was carried out using the PHYLIP package [85].

For the next generation sequencing of RNA, a series of overlapping cDNA fragments were produced using viral RNA or total RNA preparations using the set of primers designed to the sequences of the genomic RNA conserved among DWV, VDV-1 and KV (Table S1). The fragments were pooled and libraries of paired-end reads (101 nt.), about 5 million per sample, were generated using an Illumina HiSeq 2000 (GATC-Biotech). The virus genomic RNA NGS sequencing data are available in the ArrayExpress database (www.ebi.ac.uk/arrayexpress) [76] under accession number E-MTAB-1675.

The next generation sequencing of the poly(A) RNA fraction (RNA-seq) of the total RNA preparations isolated from the honeybees was carried out using Illumina HiSeq 2000 (GATC-Biotech) protocol, with about 10 million 101 nucleotide-long reads generated for each sample. The RNA-seq sequencing data are available in the EBI Sequence Read Archive [86] under accession number PRJEB5249. This RNA-seq dataset was used to calculate Shannon's diversity index values of DWV populations using the following procedure. First we selected the reads aligning to the reference DWV and VDV-1 sequences (GenBank Accession numbers GU109335 and AY251269 respectively) from the original RNA-seq libraries using Bowtie. To take into account the effect of difference in coverage of low virus levels and high virus level RNA-seq libraries we used two approaches, (i) correction for NGS error for complete libraries (Text S1 and Wood *et al.*, unpublished data) and (ii) multiple sampling. For the latter we produced five samples of 3285 reads (the lowest number of the viral reads among the libraries), which were aligned using Bowtie to the reference DWV and VDV-1 sequences, and the NGS nucleotide pileups were then generated for each nucleotide position of the reference sequences using samtools. Shannon's diversity index of the aligned nucleotides was calculated for each position in the reference sequence. Then, the average Shannon's index values were calculated for the selected regions in the reference genomes for each sample. The averages values of and standard deviation of five samples were used in the statistical analysis.

## Supporting Information

Figure S1 **Bimodal distribution of DWV accumulation in the experimental honeybee pupae.** (A) Dotplot of Ct values by experimental group, determined by qRT-PCR, showing means and 95% confidence intervals for the means. The means for C, NV and VL are not significantly different. The difference between the mean of VH and the pooled C, NV and VL is significant with *p*-value <10^−16^. (B) A histogram shows bimodality of Ct values.
(PDF)

Figure S2 **Orthogonality of the differential gene expression pattern.** A geometrical visualization of the three-stage experimental process. The first stage is “frame transfer” which includes exposure to *Varroa*-selected viruses through feeding at larval stage (contrast C to NV), the second stage is exposure to the *Varroa* mite feeding on the pupae haemolyph (contrast NV to VL) and the third stage is development of high viral load (contrast VL to VH). (A) Numbers of significantly differentially expressed genes in each of the three stages are shown alongside the directional vectors, together with numbers of differentially expressed genes in the composite stages (contrasts C to VL, NV to VH, C to VH). (B) The three stages involve distinct sets of differentially expressed genes, depicted in the graphic as orthogonality of the associated vectors; the very small number of genes common to pairs of contrasts are shown. (C) The large number of differentially expressed genes common to the pairs of non-orthogonal contrasts are shown.
(PDF)

Figure S3 **Principal component analysis (PCA) produced with 30 genes selected from the top genes from each contrast ranked by adjusted** ***p*** **-value.** The genes were selected as follows: 7 top genes were selected from each of the 6 contrasts, and the 30 with the lowest adjusted *p*-values used in subsequent analysis. The scatterplot of the first two principal components for all honeybee samples (average for Cy3 and Cy5 replicates) is shown.
(PDF)

Figure S4 **Summary of numbers of differentially expressed immune-related genes.** The number of up- and down-regulated genes in each contrast are marked, respectively, as ↑ and ↓. An up-regulated gene level is higher at the head arrow showing the contrast; commonality is shown in brackets.
(PDF)

Figure S5 **Correlation between the virus levels in honeybee pupae and the corresponding mites.** Two-dimensional plots showing the results of the qRT-PCR quantification of viral RNA in the honeybee pupae (log_10_ transformed copy number of the viral RNA per honeybee) and the corresponding *Varroa* mites (log_10_ transformed viral RNA copy number normalised to *Varroa* β-actin copy number) from experiment groups VL and VH. Panels shows results of (A) total DWV-like virus quantified with the primers recognising the NS region of DWV, VDV-1 and KV, then specific quantification of (B) VDV-1 CP, (C) VDV-1 NS, (D) DWV CP and (E) DWV NS regions.
(PDF)

Figure S6 **Quantification of negative strands of DWV RNA in the** ***Varroa*** **mites of the groups VH and VL.** The graph shows average copy number per mite of DWV- and VDV-1-like CP-coding sequence as determined by negative-strand specific qRT-PCR using primers listed in Table S1. The dotted line indicates the detection threshold as determined by a water-only control plus two standard deviations.
(PDF)

Figure S7 **Genetic diversity of DWV in the honeybee groups.** Average Shannon's diversity index values. (A) the CP region positions 1751 to 4595, (B) the NS region, positions 5008 to 9826, and the central region, positions 5250 to 6250. Positions are given for the reference DWV genome, GenBank Accession number AJ489744. Average Shannon's index was calculated for five random 3285-read samples from the viral reads for each NGS library of individual bees. Bars indicate SD. Letters above the bars represent statistically significant groupings according to Fisher's Least Significant Difference (LSD) test as 5% and 0.1% levels, marked with * and *** respectively. The dashed lines indicate the average Shannon's diversity index values for the NGS sequencing error, ± standard deviation (SD). In panel (C) the dotted line at 0.0417 marks the Shannon's diversity index for Group NV of the frame transfer experiment.
(PDF)

Table S1 **Oligonucleotides used in this study.** Primer descriptions are given as follows: target (position in DWV or VDV-1 nucleotide sequence), polarity (F, forward; R, reverse). GenBank accession numbers used to express primer positions are AJ489744 (DWV), NC_006494 (VDV-1), and AB242568 (*Varroa destructor* β-actin mRNA).
(PDF)

Table S2 **Honeybee genes differentially expressed in the experiment.** Shown are, the honeybee OGS1 gene ID, adjusted *P*-value, average expression levels in the groups (C, NV, VL, VH), and the contrasts where the given gene is differentially expressed (NV-C (1), VL-C (2), VH-C (3), VL-NV (4), VH-NV (5), VH-VL (6)).
(PDF)

Table S3 **Summary of gene expression commonality between the contrasts.** Number of differentially expressed genes common to pairs of contrasts. In each cell (corresponding to the pair of contrasts) the three figures show the commonality for all differentially expressed genes in the pair, the up-regulated genes in the pair, and the down-regulated genes in the pair respectively.
(PDF)

Table S4 **Significantly overrepresented GO terms (Biological Process, BP, terms) in the contrasts (corrected** ***p*** **-value <0.05).**
(PDF)

Table S5 **Summary of the small RNA sequencing in the experimental groups.** The single read libraries were aligned using Bowtie [37] to *Apis mellifera* miRNA [36], and to the reference full-length DWV and VDV-1 sequences, GenBank Accession numbers GU109335 and AY251269 respectively.
(PDF)

Table S6 **Correlation of virus-specific siRNA coverage between experimental groups.** Pearson correlations, P<0.001 are shown. The small RNA libraries determined by high-throughput sequencing were aligned to the DWV or VDV-1 sequences (GenBank Accession numbers GU109335 and AY251269 respectively) using bowtie [37]. All reads VDV-1 or DWV pileup values numbers of the DWV- and VDV-1- specific small RNA reads, up to 3 mismatches were allowed for the 18 nt seed region.
(PDF)

Table S7 **Summary of the NGS libraries and consensus viral sequences from individual honeybees from** ***Varroa*** **-infested colony.** RNA-seq libraries were produced using poly(A) RNA extracts. The reads were aligned to the reference full-length DWV and VDV-1 sequences, GenBank Accession numbers GU109335 and AY251269 respectively, using the “ —very-sensitive-local” option which allowed the highest number of mismatches. The aligned reads were used to generate consensus nucleotide sequences. The assembled viral sequences showed highest identity with the DWV-VDV-1 recombinant clone identified in the sampled colony (GenBank Accession number KJ437447) and the group VH sequences (e.g. GenBank Accession number JX661656).
(PDF)

Table S8 **Differential expression of putative homeobox genes in the contrasts.**
(PDF)

Text S1 **Next generation sequencing: error correction and diversity testing outline.**
(PDF)
