# Supplementary figures and images for: A Virulent Strain of Deformed Wing Virus (DWV) of Honeybees (Apis mellifera) Prevails after Varroa destructor-Mediated, or In Vitro, Transmission
Source: PLoS Pathog. 2014 Jun 26;10(6):e1004230. doi: 10.1371/journal.ppat.1004230 (PMC4072795; doi:10.1371/journal.ppat.1004230)

Figure S1. Bimodal distribution of DWV accumulation in the experimental honeybee pupae.

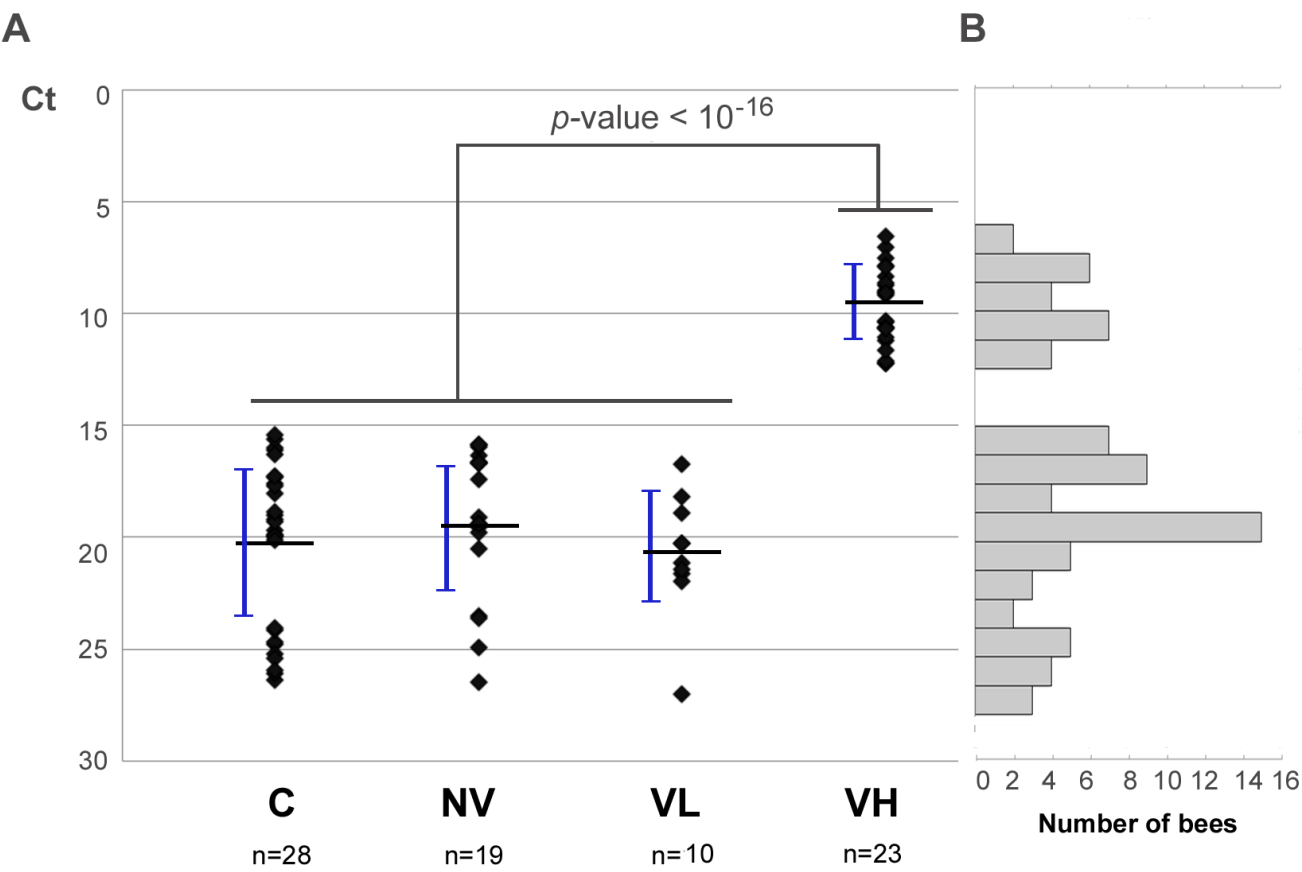

Supplement: Figure S1 — Bimodal distribution of DWV accumulation in the experimental honeybee pupae. (A) Dotplot of Ct values by experimental group, determined by qRT-PCR, showing means and 95% confidence intervals for the means. The means for C, NV and VL are not significantly different. The difference between the mean of VH and the pooled C, NV and VL is significant with p-value <10−16. (B) A histogram shows bimodality of Ct values. (PDF) [file ppat.1004230.s001.pdf]

Figure S4. Summary of differential expression of the immune-related genes.

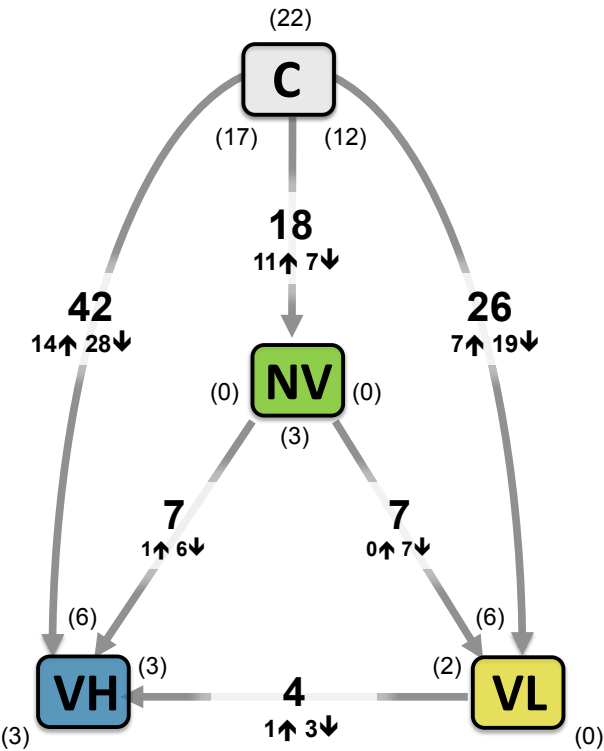

Supplement: Figure S4 — Summary of numbers of differentially expressed immune-related genes. The number of up- and down-regulated genes in each contrast are marked, respectively, as ↑ and ↓. An up-regulated gene level is higher at the head arrow showing the contrast; commonality is shown in brackets. (PDF) [file ppat.1004230.s004.pdf]

Figure S5. Quantification of DWV strains in bee pupae and corresponding mites.

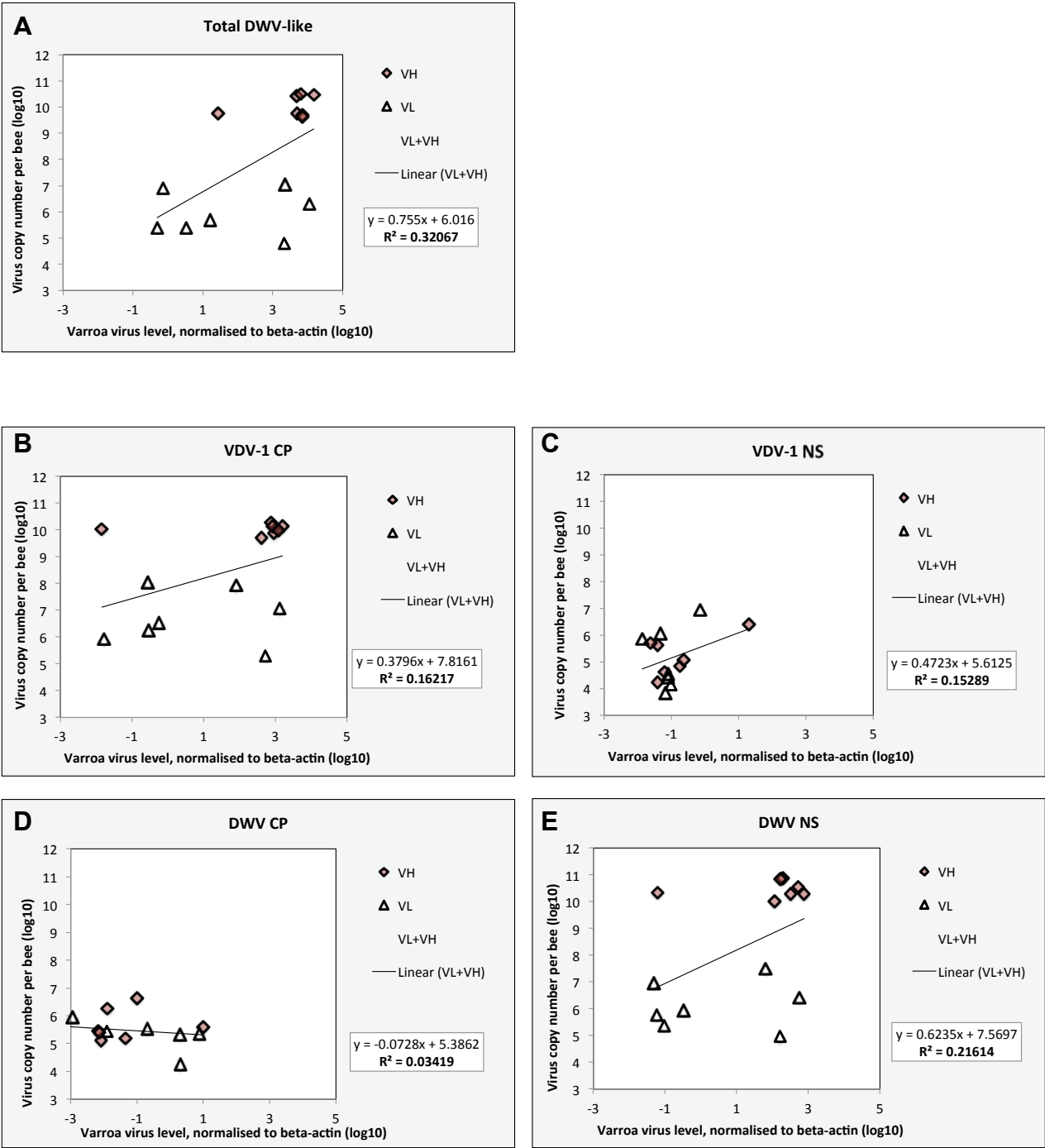

Supplement: Figure S5 — Correlation between the virus levels in honeybee pupae and the corresponding mites. Two-dimensional plots showing the results of the qRT-PCR quantification of viral RNA in the honeybee pupae (log10 transformed copy number of the viral RNA per honeybee) and the corresponding Varroa mites (log10 transformed viral RNA copy number normalised to Varroa β-actin copy number) from experiment groups VL and VH. Panels shows results of (A) total DWV-like virus quantified with the primers recognising the NS region of DWV, VDV-1 and KV, then specific quantification of (B) VDV-1 CP, (C) VDV-1 NS, (D) DWV CP and (E) DWV NS regions. (PDF) [file ppat.1004230.s005.pdf]

Figure S6. Quantification of negative strands of DWV RNA in the *Varroa* mites.

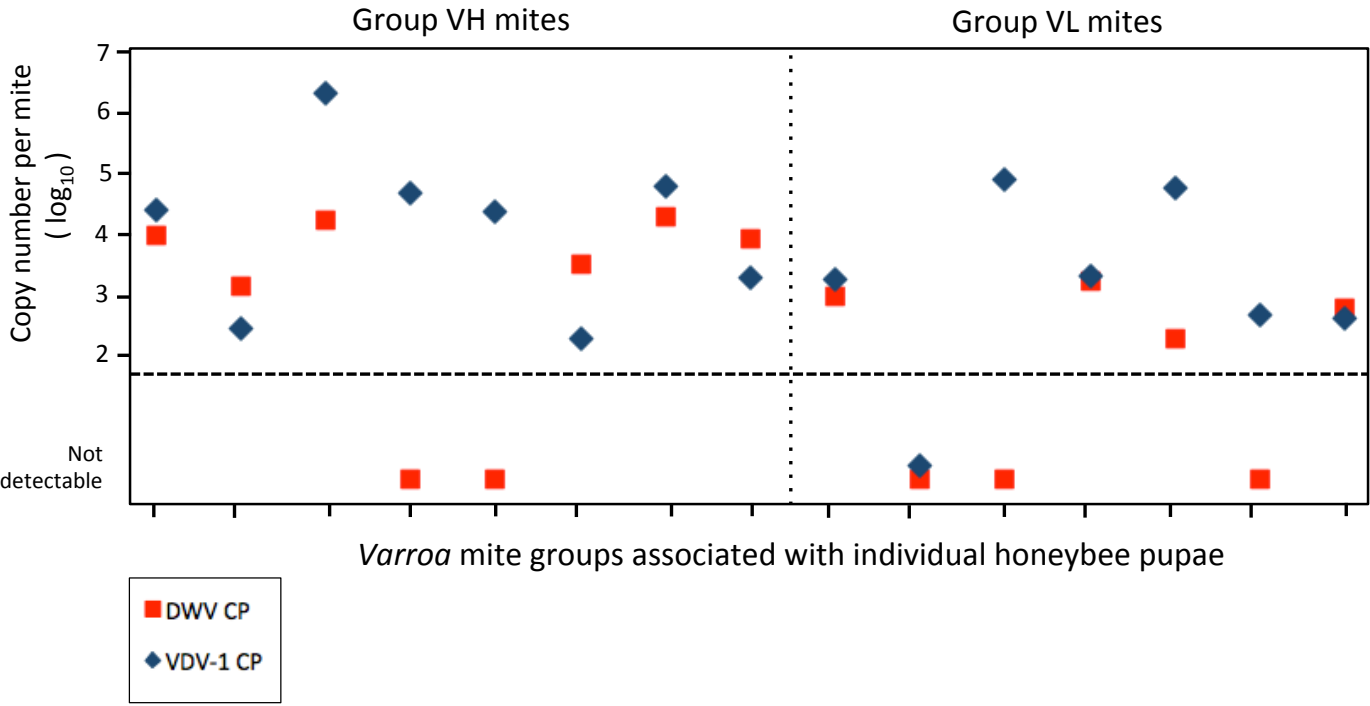

Supplement: Figure S6 — Quantification of negative strands of DWV RNA in the Varroa mites of the groups VH and VL. The graph shows average copy number per mite of DWV- and VDV-1-like CP-coding sequence as determined by negative-strand specific qRT-PCR using primers listed in Table S1. The dotted line indicates the detection threshold as determined by a water-only control plus two standard deviations. (PDF) [file ppat.1004230.s006.pdf]

Figure S7. Genetic diversity of DWV in the honeybee groups.

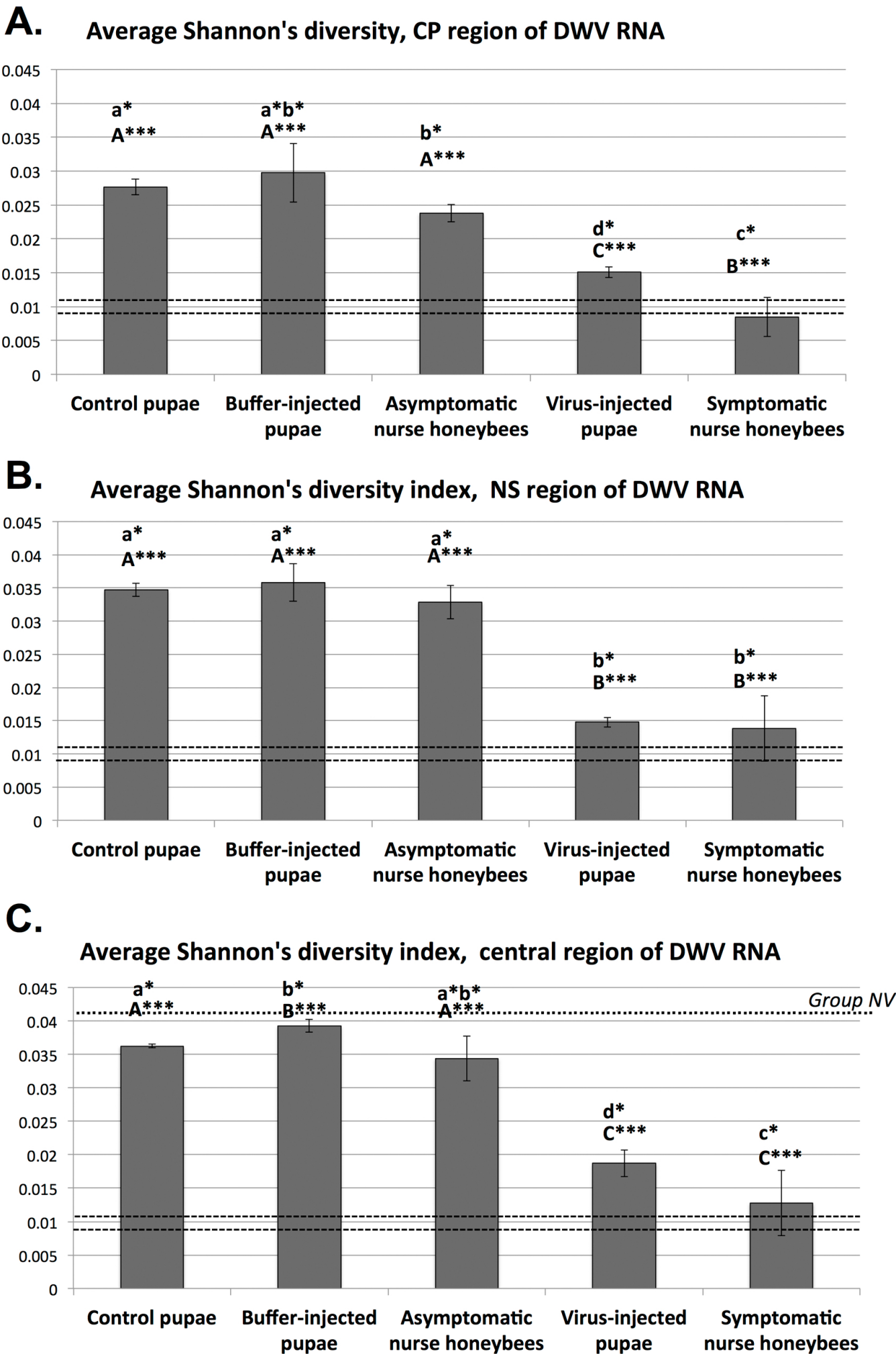

Supplement: Figure S7 — Genetic diversity of DWV in the honeybee groups. Average Shannon's diversity index values. (A) the CP region positions 1751 to 4595, (B) the NS region, positions 5008 to 9826, and the central region, positions 5250 to 6250. Positions are given for the reference DWV genome, GenBank Accession number AJ489744. Average Shannon's index was calculated for five random 3285-read samples from the viral reads for each NGS library of individual bees. Bars indicate SD. Letters above the bars represent statistically significant groupings according to Fisher's Least Significant Difference (LSD) test as 5% and 0.1% levels, marked with * and *** respectively. The dashed lines indicate the average Shannon's diversity index values for the NGS sequencing error, ± standard deviation (SD). In panel (C) the dotted line at 0.0417 marks the Shannon's diversity index for Group NV of the frame transfer experiment. (PDF) [file ppat.1004230.s007.pdf]
