# Supplementary material for: A Virulent Strain of Deformed Wing Virus (DWV) of Honeybees (Apis mellifera) Prevails after Varroa destructor-Mediated, or In Vitro, Transmission
Source: PLoS Pathog. 2014 Jun 26;10(6):e1004230. doi: 10.1371/journal.ppat.1004230 (PMC4072795; doi:10.1371/journal.ppat.1004230)

Figure S2. Differential expression of independent gene sets in response to oral DWV infection, exposure to *Varroa* mite feeding, and high DWV levels.

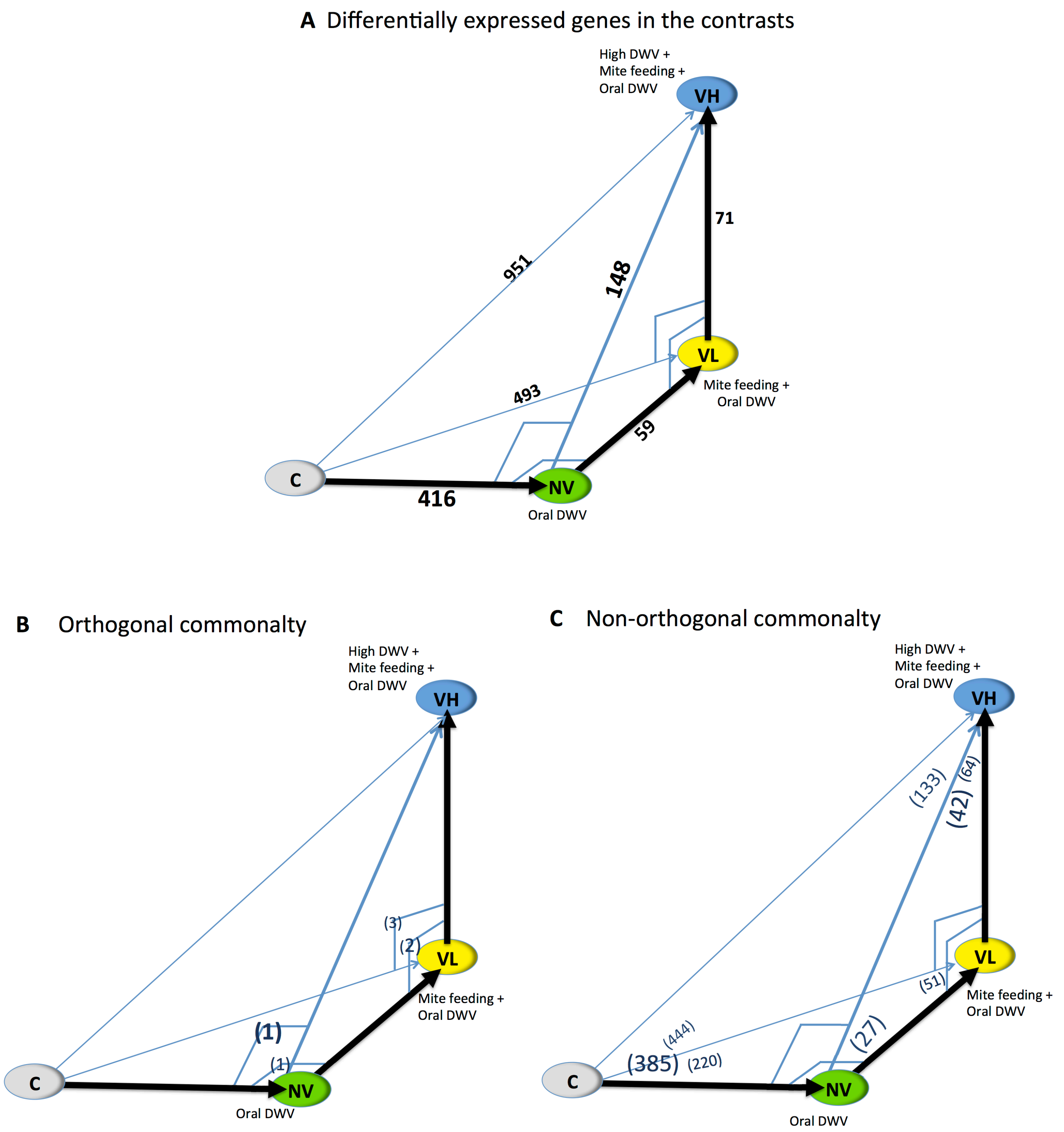

Supplement: Figure S2 — Orthogonality of the differential gene expression pattern. A geometrical visualization of the three-stage experimental process. The first stage is “frame transfer” which includes exposure to Varroa-selected viruses through feeding at larval stage (contrast C to NV), the second stage is exposure to the Varroa mite feeding on the pupae haemolyph (contrast NV to VL) and the third stage is development of high viral load (contrast VL to VH). (A) Numbers of significantly differentially expressed genes in each of the three stages are shown alongside the directional vectors, together with numbers of differentially expressed genes in the composite stages (contrasts C to VL, NV to VH, C to VH). (B) The three stages involve distinct sets of differentially expressed genes, depicted in the graphic as orthogonality of the associated vectors; the very small number of genes common to pairs of contrasts are shown. (C) The large number of differentially expressed genes common to the pairs of non-orthogonal contrasts are shown. (PDF) [file ppat.1004230.s002.pdf]
