# Supplementary material for: A Virulent Strain of Deformed Wing Virus (DWV) of Honeybees (Apis mellifera) Prevails after Varroa destructor-Mediated, or In Vitro, Transmission
Source: PLoS Pathog. 2014 Jun 26;10(6):e1004230. doi: 10.1371/journal.ppat.1004230 (PMC4072795; doi:10.1371/journal.ppat.1004230)

Figure S3. Principal component analysis (PCA) produced with 30 genes selected from the top genes from each contrast ranked by adjusted *p*-value.

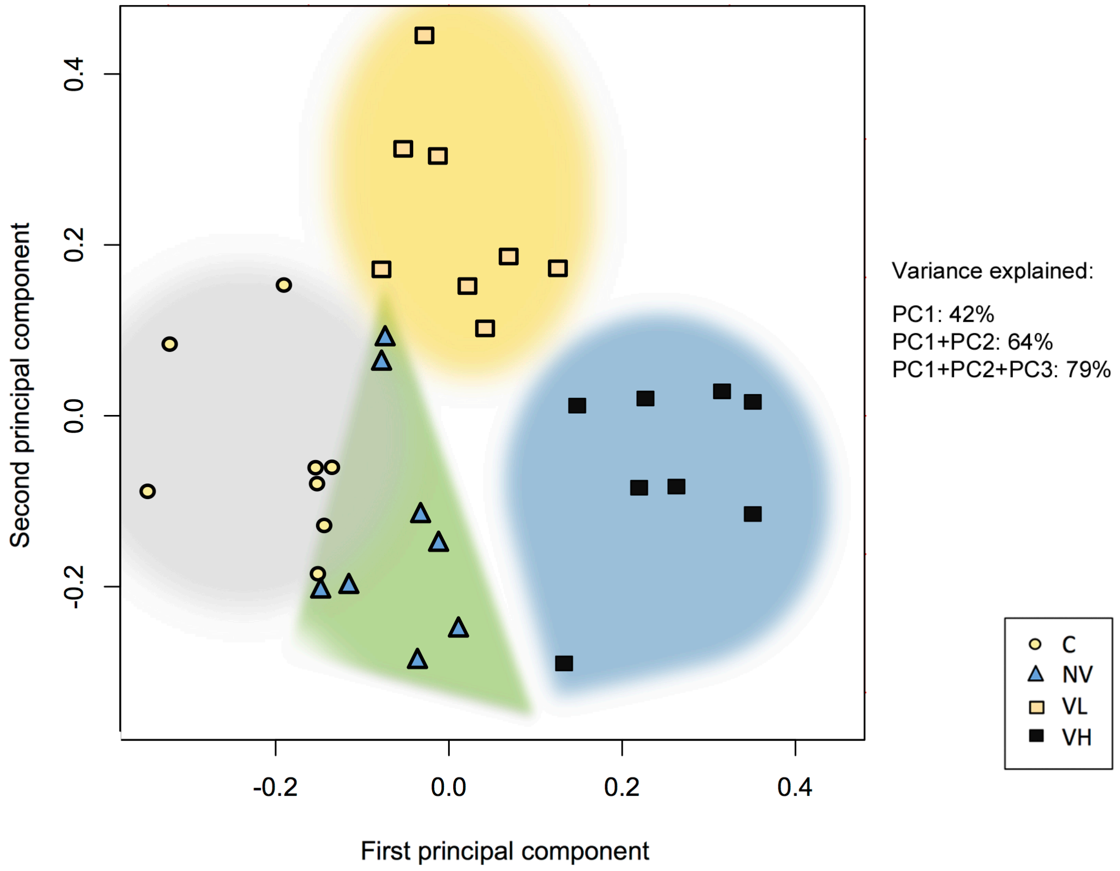

Supplement: Figure S3 — Principal component analysis (PCA) produced with 30 genes selected from the top genes from each contrast ranked by adjusted p -value. The genes were selected as follows: 7 top genes were selected from each of the 6 contrasts, and the 30 with the lowest adjusted p-values used in subsequent analysis. The scatterplot of the first two principal components for all honeybee samples (average for Cy3 and Cy5 replicates) is shown. (PDF) [file ppat.1004230.s003.pdf]
