# Supplementary material for: A Virulent Strain of Deformed Wing Virus (DWV) of Honeybees (Apis mellifera) Prevails after Varroa destructor-Mediated, or In Vitro, Transmission
Source: PLoS Pathog. 2014 Jun 26;10(6):e1004230. doi: 10.1371/journal.ppat.1004230 (PMC4072795; doi:10.1371/journal.ppat.1004230)
Supplement: Table S1 — Oligonucleotides used in this study. Primer descriptions are given as follows: target (position in DWV or VDV-1 nucleotide sequence), polarity (F, forward; R, reverse). GenBank accession numbers used to express primer positions are AJ489744 (DWV), NC_006494 (VDV-1), and AB242568 (Varroa destructor β-actin mRNA). (PDF) [file ppat.1004230.s008.pdf]

Table S1. Oligonucleotides used in this study

Primer ID	Sequence (5'–3)	Description	Appication
**59**	GTTTGTATGAGGTTATACTTCAAGGAG	DWV/VDV-1 (8004-8030), F	qRT-PCR, Array validation
**60**	GCCATGCAATCCTTCAGTACCAGC	DWV/VDV-1 (8143-8120), R	qRT-PCR, Array validation
**1381**	CTGTAGTTAAGCGGTTATTAGAA	VDV-1 CP (4890-4912), F	qRT-PCR, RT-PCR-central region
**1382**	GGTGCTTCTGGAACAGCGGAA	VDV-1 CP (4986-4966), R	qRT-PCR, Negative strand qRT-PCR
**1383**	CTGTAGTCAAGCGGTTACTTGAG	DWV CP (4917-4939), F	qRT-PCR, RT-PCR-central region
**1384**	GGAGCTTCTGGAACGGCAGGT	DWV CP (5013-4993), R	qRT-PCR, Negative strand qRT-PCR
**1425**	TTCATTAAAACCGCCAGGCTCT	VDV-1 NS (8623-8644), F	qRT-PCR
**1426**	CAAGTTCAGGTCTCATCCCTCT	VDV-1 NS (8723-8702), R	qRT-PCR
**1427**	TTCATTAAAGCCACCTGGAACA	DWV NS (8650-8671), F	qRT-PCR, Array validation
**1428**	CAAGTTCGGGACGCATTCCACG	DWV NS (8750-8729), R	qRT-PCR, Array validation
**1418**	TGAAGGTAGTCTCATGGATAC	Varroa β-actin, R	qRT-PCR
**1419**	GTCTCTGTTCCAGCCCTCGTTC	Varroa β-actin F	qRT-PCR
**1420**	AGGAATGGAAGCTTGCGGTA	Honeybee β-actin, F	qRT-PCR
**1421**	AATTTTCATGGTGGATGGTGC	Honeybee β-actin, R	qRT-PCR
**155**	CAGTAGCTTGGGCGATTGTTTCG	DWV/VDV-1 (4842-4864), F,	RT-PCR-central region
**156**	CGCGCTTAACACACGCAAATTATC	DWV/VDV-1 (6747-6728), R	RT-PCR-central region
**153**	CTTGGAGCTTGAGGCTCTACA	DWV (6546-6526), R	RT-PCR-central region
**154**	CTGAAGTACTAATCTCTGAG	VDV-1 (6308-6289), R	RT-PCR-central region
**211**	GCCTTCCATAGCGAATTACG	DWV/VDV-1 (9-28), F	RT-PCR, DWV cDNA for NGS
**213**	TTTTCAATTTAATTTTGATTTCGAAGG	DWV/VDV-1 (1092-1118), F	RT-PCR, DWV cDNA for NGS
**214**	CGCCGCCTAGCTTCATCA	DWV/VDV-1 (1245-1228), R	RT-PCR, DWV cDNA for NGS
**217**	GGATGATCCATTTGATAAGG	DWV/VDV-1 (1990-2009), F	RT-PCR, DWV cDNA for NGS
**218**	CATATAGCATCAGAATTAGCCTC	DWV/VDV-1 (2076-2054), R	RT-PCR, DWV cDNA for NGS
**221**	GGGTGCGTAAATATGGTGG	DWV/VDV-1 (3159-3177), F	RT-PCR, DWV cDNA for NGS
**222**	TAGTATCTGAAACAGCTTCC	DWV/VDV-1 (3269-3250), R	RT-PCR, DWV cDNA for NGS
**231**	TGCCTGAGGGCCCTATTGCGAAG	DWV/VDV-1 (4614-4636), F	RT-PCR, DWV cDNA for NGS
**224**	ACCATACCCATATCTTCACGCATC	DWV/VDV-1 (4716-4693), R	RT-PCR, DWV cDNA for NGS
**233**	CAGAGATTGAAGCGCATGAACAAG	DWV/VDV-1 (6474-6497), F	RT-PCR, DWV cDNA for NGS
**234**	GCACTTAACACACGCAAATTATC	DWV/VDV-1 (6750-6728), R	RT-PCR, DWV cDNA for NGS
**237**	GAGTATATACTTATCCATACCATG	DWV/VDV-1 (8028-8051), F	RT-PCR, DWV cDNA for NGS
**238**	CATGCAATCCTTCAGTACCAGC	DWV/VDV-1 (8141-8120), R	RT-PCR, DWV cDNA for NGS
**240**	GAGTAACACCTAACCTGAGTACC	DWV/VDV-1 (10081-10066), R	RT-PCR, DWV cDNA for NGS
**459**	CGGGAGACGCCAGGTTAG	AFB-*P. larvae*, F	qPCR, *Paenibacillus larvae* detection
**460**	TTCTTCCTTGGCAACAGAGC	AFB-*P. larvae*, R	qPCR, *Paenibacillus larvae* detection
**461**	TGTTGTTAGAGAAGAATAGGGGAA	EFB-*M. plutonius,* F	qPCR, *Melissococcus plutoniu*s detection
**462**	CGTGGCTTTCTGGTTAGA	EFB-*M. plutonius*, R	qPCR, *Melissococcus plutoniu*s detection
**147**	CAAAAAAACTCGTCATATGTTGCCAACTG	Honeybee Rp49 (GB10903), F	qRT-PCR, Array validation
**148**	GCATCATTAAAACTTCCAGTTCCTTG	Honeybee Rp49 (GB10903), R	qRT-PCR, Array validation
**274**	GGGTGTTTACACCGCGATTTATTCG	Honeybee persephone (GB14044), F	qRT-PCR, Array validation
**275**	GATATTTTTTTTCTCCACATTTTTATTCGTCC	honeybee persephone (GB14044), F	qRT-PCR, Array validation
**276**	CTCTATCTCAAGACCAACACCTACTTGC	Honeybee Tollo (GB10640), F	qRT-PCR, Array validation
**277**	CTGATTGTTGGGCACGTCCGGCAACGC	Honeybee Tollo (GB10640), R	qRT-PCR, Array validation
**321**	GTCATAGCGATCGTTTTCGCTG	Honeybee Vago (GB10896), F	qRT-PCR, Array validation
**322**	GCTATAATACGACTCACTATAGGGCAATTAGGG AATGCAGC	Honeybee Vago (GB10896), R	qRT-PCR, Array validation
**286**	CACAATCTCAAAAATGGATGTTGATACG	Honeybee transaminase (GB13140), F	qRT-PCR, Array validation
**287**	AATTAATAACTAATTAGCGAACGAGCAATGG	Honeybee transaminase (GB13140), R	qRT-PCR, Array validation
**290**	CTGCAAAATGGAGCCCTCTATGGATTGAG	Honeybee cysteine-rich (GB16716), F	qRT-PCR, Array validation
**291**	CAAATAATGCACCTTTGGATCTCTTTTAGCTG	Honeybee cysteine-rich (GB16716), R	qRT-PCR, Array validation
**388**	CTTGGTTAGCTGTGTTGCAGTTG	Adapter, F	Negative strand qRT-PCR
**389**	CTTGGTTAGCTGTGTTGCAGTTGCTGTAGTTA AGCGGTTATTAGAA	Adapter-VDV-1 CP (4890-4912), F	RT, Negative viral RNA strand quantification
**391**	CTTGGTTAGCTGTGTTGCAGTTGCTGTAGTCA AGCGGTTACTTGAG	Adapter-DWV CP (4917-4939), F	RT, Negative viral RNA strand quantification

Primer descriptions are given as follows: target (position in DWV or VDV-1 nucleotide sequence), polarity (F, forward; R, reverse). GenBank accession numbers used to expressprimer positions are AJ489744 (DWV), NC_006494 (VDV-1), NM_001185146 (Apis mellifera β-actin mRNA) and AB242568 (Varroa destructor β-actin mRNA).
