# Supplementary material for: A Virulent Strain of Deformed Wing Virus (DWV) of Honeybees (Apis mellifera) Prevails after Varroa destructor-Mediated, or In Vitro, Transmission
Source: PLoS Pathog. 2014 Jun 26;10(6):e1004230. doi: 10.1371/journal.ppat.1004230 (PMC4072795; doi:10.1371/journal.ppat.1004230)
Supplement: Table S2 — Honeybee genes differentially expressed in the experiment. Shown are, the honeybee OGS1 gene ID, adjusted P-value, average expression levels in the groups (C, NV, VL, VH), and the contrasts where the given gene is differentially expressed (NV-C (1), VL-C (2), VH-C (3), VL-NV (4), VH-NV (5), VH-VL (6)). (PDF) [file ppat.1004230.s009.pdf]

Table S2. Differentially expressed (DE) genes. Shown are adjusted *p*-values, mean log2 transformed expression levels in the experimental groups and the contrasts where the gene is DE ((NV-C (1), VL-C (2), VH-C (3), VL-NV (4), VH-NV (5), VH-VL (6)).

Apis mellifera OGS1	Adj. *p*-value	Mean expression level (log2 transformed)				DE in contrasts
		Group C	Group NV	Group VL	Group VH	
GB15279-PA	1.3E-21	5.723	5.755	5.650	6.917	356
GB19555-PA	4.6E-14	5.937	5.911	5.882	6.505	356
GB14206-PA	1.6E-09	9.079	9.073	9.117	9.542	356
GB15887-PA	9.4E-09	6.939	6.849	6.810	7.293	356
GB13621-PA	2.2E-08	9.010	9.038	8.967	8.636	356
GB19012-PA	3.3E-08	8.345	8.870	8.897	9.043	123
GB11756-PA	4.1E-08	6.593	6.842	6.637	7.064	356
GB17777-PA	1.3E-07	8.313	8.178	7.948	7.933	2345
GB12044-PA	1.5E-07	8.896	9.012	9.104	9.230	235
GB17879-PA	2.8E-07	9.622	9.437	9.452	9.090	356
GB15698-PA	4E-07	7.248	6.944	6.860	6.684	123
GB19623-PA	7.3E-07	8.420	8.404	8.063	8.076	2345
GB14706-PA	7.7E-07	9.643	9.435	9.319	9.105	235
GB11100-PA	1.2E-06	9.733	9.630	9.309	9.318	2345
GB18287-PA	1.4E-06	10.940	10.686	10.424	10.508	123
GB11771-PA	1.4E-06	11.163	10.942	11.052	10.654	356
GB17327-PA	1.6E-06	9.809	9.656	9.508	9.438	235
GB18663-PA	3E-06	9.728	9.370	8.898	9.149	23
GB10244-PA	3E-06	9.824	10.133	9.983	10.296	136
GB10198-PA	3E-06	9.454	9.573	9.782	9.878	235
GB15101-PA	3E-06	8.121	8.016	7.821	7.881	23
GB11751-PA	3E-06	7.968	7.879	7.708	7.654	235
GB16048-PA	3.5E-06	9.915	9.719	9.422	9.497	234
GB12520-PA	4.5E-06	7.873	7.971	8.082	8.119	23
GB18727-PA	1E-05	10.548	10.504	10.638	10.825	35
GB16387-PA	1E-05	6.380	6.376	6.382	6.655	356
GB14922-PA	1E-05	7.403	7.383	7.420	7.599	35
GB19460-PB	1E-05	10.670	10.522	10.157	10.138	2345
GB12818-PA	1E-05	8.692	8.076	8.089	7.989	123
GB19215-PA	1E-05	8.665	8.965	8.907	9.009	123
GB19452-PA	1.2E-05	10.382	11.175	11.337	11.869	123
GB11598-PA	1.2E-05	9.198	9.046	8.585	8.915	234
GB16405-PA	1.2E-05	7.931	7.518	7.415	7.342	123
GB15155-PA	1.2E-05	9.068	8.845	8.503	8.639	234
GB19917-PA	1.2E-05	8.141	8.281	8.522	8.408	234
GB18216-PA	1.2E-05	8.387	7.997	7.879	8.347	1256
GB14028-PA	1.2E-05	6.880	6.755	6.508	6.576	234
GB17262-PA	1.2E-05	8.533	8.352	8.237	8.218	23
GB14497-PA	1.2E-05	7.927	7.772	7.615	7.608	23
GB14620-PA	1.3E-05	11.670	10.876	10.948	10.740	123
GB17057-PA	1.5E-05	8.510	8.336	8.300	8.056	356
GB14921-PA	1.5E-05	8.292	8.297	7.976	8.011	2345
GB10061-PA	1.5E-05	6.762	6.584	6.375	6.450	23
GB12739-PA	1.6E-05	8.334	8.223	8.090	8.019	235
GB14010-PA	1.6E-05	6.932	7.220	7.118	7.852	356
GB10694-PA	1.6E-05	6.546	7.212	7.243	7.046	123
GB11307-PA	1.6E-05	9.691	9.488	9.295	9.231	235
GB19755-PA	1.6E-05	7.657	7.560	7.438	7.094	35
GB15820-PA	1.7E-05	6.432	6.315	6.182	6.151	23
GB10382-PA	1.7E-05	10.668	10.563	10.353	10.370	234
GB15480-PA	1.7E-05	8.444	8.435	8.264	8.230	35

Apis mellifera OGS1	Adj. *p*-value	Mean expression level (log2 transformed)				DE in contrasts
		Group C	Group NV	Group VL	Group VH	
GB15110-PA	1.7E-05	6.783	7.119	7.102	7.157	123
GB13036-PA	1.7E-05	7.613	7.302	7.458	7.084	13
GB13354-PA	1.9E-05	9.188	8.983	8.992	8.957	3
GB13703-PA	1.9E-05	10.274	10.137	10.022	9.965	23
GB12330-PA	1.9E-05	10.673	10.321	10.843	9.998	36
GB12810-PA	1.9E-05	7.516	7.621	7.170	7.321	245
GB19425-PA	1.9E-05	10.013	9.710	9.576	9.581	123
GB19017-PA	2E-05	7.857	8.226	7.988	8.713	356
GB11739-PA	2E-05	7.998	7.975	7.870	7.699	35
GB19190-PA	3E-05	8.072	8.049	7.971	7.816	35
GB14431-PA	3E-05	8.128	7.956	7.831	7.819	23
GB12991-PA	3E-05	9.730	9.326	9.238	9.171	123
GB12079-PA	3.1E-05	8.134	8.092	7.956	7.897	3
GB14400-PA	3.3E-05	7.340	7.243	7.023	7.055	234
GB14446-PA	3.4E-05	7.155	7.403	7.059	6.749	35
GB17474-PA	3.5E-05	9.496	9.914	10.015	10.166	123
GB16103-PA	3.5E-05	8.322	8.396	8.291	8.182	5
GB14098-PA	3.5E-05	8.968	8.803	8.740	8.718	23
GB17458-PA	3.5E-05	9.349	9.179	8.962	8.922	235
GB11446-PA	3.6E-05	6.187	6.376	6.491	6.431	23
GB13450-PA	3.6E-05	6.631	6.622	6.698	6.465	6
GB18742-PA	3.6E-05	9.210	9.076	8.981	8.910	23
GB13359-PA	3.6E-05	9.669	9.534	9.419	9.349	23
GB11539-PA	3.6E-05	9.492	9.214	9.020	8.890	23
GB13601-PA	3.6E-05	8.798	7.890	7.785	8.186	123
GB15795-PA	3.9E-05	7.276	7.026	7.123	6.991	13
GB15534-PA	4.1E-05	8.966	8.778	8.670	8.618	23
GB10155-PA	4.4E-05	10.767	10.722	10.725	10.975	356
GB14653-PA	4.7E-05	6.798	6.526	6.508	6.353	123
GB17524-PA	5.3E-05	8.339	8.522	8.592	8.560	23
GB14467-PA	5.3E-05	11.186	11.144	10.981	10.715	235
GB10102-PA	5.4E-05	12.770	12.406	11.958	11.883	235
GB17489-PA	5.7E-05	8.998	9.162	9.315	9.692	356
GB17182-PA	5.7E-05	7.293	7.998	7.866	7.939	123
GB10156-PA	5.7E-05	8.679	8.829	8.847	8.976	3
GB12475-PA	5.7E-05	9.047	8.994	8.707	8.767	2345
GB18806-PA	5.7E-05	12.430	11.725	11.762	11.551	123
GB10499-PA	5.7E-05	9.012	8.480	8.571	7.976	3
GB11686-PA	6.5E-05	9.420	9.291	9.185	9.083	235
GB17653-PA	7.4E-05	8.612	8.493	8.284	8.351	23
GB17001-PA	7.8E-05	6.304	6.465	6.430	6.854	356
GB17945-PB	7.8E-05	6.380	6.307	6.123	6.193	2
GB18348-PB	7.9E-05	6.724	6.730	6.445	6.475	2345
gb16002-PA	8E-05	6.421	6.097	5.972	5.937	123
GB14165-PA	8.4E-05	8.882	9.078	8.886	9.235	36
GB19274-PA	9E-05	9.636	9.466	9.384	9.217	23
GB14022-PA	9.1E-05	6.309	6.384	6.399	6.772	356
GB19945-PA	9.2E-05	6.469	6.520	6.485	6.858	356
GB10283-PA	9.3E-05	11.076	10.826	10.749	10.642	123
GB16686-PA	9.6E-05	13.753	14.238	14.407	14.195	123
GB11223-PA	9.9E-05	10.205	9.784	9.797	9.715	123

GB19201-PA	0.0001	9.001	8.483	8.509	8.834	12
GB14614-PA	0.0001	6.639	6.808	6.877	7.074	23
GB10983-PA	0.0001	7.825	7.794	7.518	7.598	234
GB12698-PA	0.00011	8.877	9.013	9.172	9.177	23
GB17021-PA	0.00011	10.210	9.861	9.873	9.527	356
GB13670-PA	0.00011	8.498	7.787	7.632	7.255	123
GB18662-PA	0.00011	7.660	7.864	7.803	9.253	356
GB19902-PA	0.00011	8.886	8.674	8.625	8.561	23
GB13173-PA	0.00012	9.697	9.509	8.967	8.962	2345
GB16752-PA	0.00012	7.632	7.807	7.726	8.141	356
GB17052-PA	0.00012	7.576	7.376	7.359	7.308	23
GB14746-PA	0.00013	8.006	7.835	7.676	7.655	23
GB15331-PA	0.00013	9.352	8.854	8.919	8.619	123
GB11384-PA	0.00013	7.321	8.153	8.320	7.554	12
GB18886-PA	0.00013	9.298	9.171	9.145	9.045	3
GB10124-PA	0.00013	10.275	10.531	10.053	9.980	2345
GB19168-PA	0.00013	10.690	10.459	10.476	10.342	123
GB17498-PA	0.00013	10.833	10.625	10.736	10.468	36
GB18744-PA	0.00013	9.272	8.961	8.875	8.835	123
GB10341-PA	0.00013	7.519	7.301	7.101	6.951	235
GB18340-PA	0.00013	6.603	6.875	6.859	6.862	123
GB13881-PA	0.00014	9.073	9.117	8.938	8.856	35
GB10720-PA	0.00014	9.649	9.621	9.477	9.419	3
GB13747-PB	0.00014	10.612	10.408	10.115	10.005	235
GB12503-PA	0.00014	6.869	6.770	6.477	6.616	234
GB15719-PA	0.00014	7.741	7.744	7.488	7.568	24
GB17672-PA	0.00014	8.412	8.176	8.173	8.032	123
GB10771-PA	0.00014	10.667	10.382	10.425	10.081	3
GB14500-PA	0.00015	6.707	6.800	6.755	6.970	3
GB19929-PA	0.00016	7.526	7.625	7.194	7.322	245
GB11411-PA	0.00016	6.506	6.384	6.267	6.278	23
GB19306-PA	0.00017	7.115	7.164	7.110	6.838	356
GB12555-PA	0.00017	7.919	7.766	7.715	7.495	35
GB17870-PA	0.00017	9.901	9.551	9.543	9.473	123
GB11262-PA	0.00017	8.020	8.367	8.150	8.403	13
GB15728-PA	0.00017	7.740	7.941	7.902	8.051	13
GB11987-PA	0.00017	6.737	6.771	6.756	6.955	3
GB12248-PA	0.00018	7.760	8.053	7.950	8.074	13
GB19582-PA	0.00018	10.082	10.561	10.582	10.689	123
GB18075-PA	0.00018	8.543	8.395	8.391	8.318	3
GB10601-PA	0.00018	6.547	6.345	6.416	6.289	13
GB12274-PA	0.00018	8.536	8.292	8.359	8.261	13
GB16624-PA	0.00018	9.205	8.976	8.881	8.776	123
GB19016-PB	0.00018	8.258	8.540	8.393	8.586	13
GB12838-PA	0.00018	9.057	8.835	8.970	8.763	13
GB11131-PA	0.00018	7.379	7.241	7.202	7.065	3
GB14812-PA	0.00018	7.327	7.139	7.038	6.891	23
GB16022-PA	0.00018	6.790	6.696	6.657	6.581	3
GB15133-PA	0.00018	9.210	9.110	8.897	8.840	235
GB14435-PA	0.00018	9.188	9.342	9.314	9.965	356
GB11194-PA	0.00019	8.584	8.479	8.342	8.319	23
GB14026-PA	0.00019	9.183	8.859	8.788	8.709	123
GB10377-PA	0.00019	8.599	8.814	8.752	8.944	13
GB15157-PA	0.00019	9.486	9.346	9.320	9.175	3
GB12899-PA	0.0002	8.650	9.363	9.153	9.229	123
GB11892-PA	0.0002	9.354	9.085	8.906	8.979	123
GB13245-PA	0.00021	6.883	7.233	7.154	7.208	123
GB15263-PA	0.00021	9.822	9.665	9.586	9.505	23
GB18517-PA	0.00021	9.616	9.365	9.113	9.168	23
GB17125-PA	0.00021	10.471	10.276	10.310	9.915	356
GB19508-PA	0.00021	7.007	6.811	6.854	6.709	3
GB18434-PA	0.00022	9.979	9.928	9.753	9.678	235
GB11921-PA	0.00022	10.343	10.200	10.076	10.068	23
GB17212-PA	0.00022	7.588	7.782	7.838	7.764	23
GB12586-PA	0.00023	10.938	10.872	10.715	10.557	235
GB15452-PA	0.00023	9.360	9.901	9.754	9.991	123
GB17415-PA	0.00024	7.077	7.002	7.007	6.866	3
GB17184-PA	0.00024	7.925	7.774	7.742	7.637	23
GB12824-PA	0.00025	9.173	9.410	9.499	9.573	123
GB19970-PA	0.00025	9.185	9.077	8.843	8.719	235
GB14958-PA	0.00025	9.688	9.269	9.286	9.093	123
GB14279-PA	0.00025	8.529	8.570	8.669	8.761	3
GB17511-PA	0.00026	10.522	10.689	10.824	10.935	23
GB18403-PA	0.00026	6.179	6.248	6.219	6.431	3
GB12842-PA	0.00026	6.948	7.108	7.121	7.246	3
GB16496-PA	0.00026	6.966	6.924	6.732	6.745	23
GB18488-PA	0.00027	7.669	7.530	7.314	7.241	235
GB15837-PA	0.00028	11.361	11.234	10.923	10.874	235
GB19791-PA	0.00028	8.870	8.772	8.647	8.566	235
GB15154-PA	0.00028	9.679	9.787	9.909	9.820	2
GB18332-PA	0.00029	9.786	10.497	10.544	10.208	123
GB10910-PA	0.0003	15.086	15.692	15.740	15.835	123
GB11674-PA	0.0003	9.472	9.580	9.636	9.761	35
GB12444-PA	0.0003	7.209	6.905	6.869	6.795	123
GB12464-PA	0.0003	6.690	6.573	6.456	6.475	23
GB19988-PA	0.0003	11.547	11.233	11.180	11.054	123
AmelCPF1-PA	0.0003	8.646	7.802	7.612	8.254	123
GB10426-PA	0.00031	10.295	10.004	9.900	9.848	123
GB16871-PA	0.00031	9.720	9.461	9.287	9.234	23
GB16304-PA	0.00033	9.174	9.603	9.296	9.632	136
GB19967-PA	0.00033	10.160	10.578	10.254	10.714	136
GB13592-PA	0.00033	9.166	9.219	9.099	8.959	5
GB18036-PA	0.00033	7.581	7.505	7.443	7.341	3
GB15428-PA	0.00033	10.986	11.370	11.196	11.591	13
GB14744-PA	0.00035	9.753	9.602	9.465	9.488	23
GB19386-PA	0.00035	10.667	11.212	11.106	11.262	123
GB19626-PA	0.00035	7.568	8.642	8.736	9.548	123
GB15246-PA	0.00035	6.932	6.942	6.870	7.501	356
GB16951-PA	0.00035	9.457	9.613	9.708	9.893	23
GB18634-PA	0.00035	8.310	8.485	8.551	8.714	23
gb16773-PA	0.00036	8.309	8.182	7.889	7.773	235
GB19961-PA	0.00037	9.398	9.227	9.118	9.006	23
GB17993-PA	0.00037	6.915	6.700	6.802	6.710	13
GB11348-PA	0.00038	10.596	10.379	10.410	10.306	13
GB19847-PA	0.00038	8.190	7.963	7.633	7.870	23
GB15381-PA	0.00039	7.538	7.462	7.414	7.267	3
GB10553-PA	0.00039	6.686	6.987	7.000	6.925	123
GB18649-PA	0.0004	8.961	8.764	8.716	8.736	23
GB12212-PA	0.0004	9.264	9.633	9.443	9.636	13
GB18083-PA	0.0004	7.037	6.918	6.852	6.699	23
GB13838-PA	0.0004	8.922	8.784	8.755	8.555	3
GB19122-PA	0.0004	7.349	8.152	8.219	7.920	123
GB11315-PA	0.00042	9.071	9.198	8.920	9.071	4
GB18012-PA	0.00044	10.042	9.999	9.943	9.825	3
GB15688-PA	0.00045	7.949	7.803	7.916	7.584	356
GB10404-PA	0.00045	7.563	7.372	7.226	7.165	23
GB10442-PA	0.00045	8.947	8.750	8.743	8.657	23
GB10128-PA	0.00045	9.628	9.491	9.284	9.140	235
GB10775-PA	0.00046	8.008	7.905	7.713	7.761	23
GB11992-PA	0.00048	7.991	8.073	8.086	8.291	356
GB17978-PA	0.00049	7.708	7.483	7.542	7.469	13
GB19886-PA	0.00049	9.684	9.541	9.251	9.295	23

TAMU:temp11 :Chromosome 3_Group3	0.00049	10.815	11.666	11.402	11.645	123
GB17650-PA	0.00049	9.744	9.686	9.491	9.406	235
GB14649-PA	0.00049	7.084	6.912	6.732	6.657	23
GB20063-PA	0.00051	10.637	10.615	10.323	10.387	2345
GB10522-PA	0.00051	7.867	7.882	7.509	7.531	2345
GB13646-PA	0.00052	8.772	8.716	8.527	8.489	235
GB14489-PA	0.00052	9.525	9.179	9.087	9.051	123
GB15846-PA	0.00052	8.332	8.436	8.452	8.587	3
GB10950-PA	0.00053	7.163	7.063	6.969	6.968	3
GB17684-PA	0.00053	7.157	7.446	7.448	7.506	123
GB18237-PA	0.00053	6.924	7.650	7.856	7.527	123
GB15993-PA	0.00054	8.267	8.679	8.711	8.784	123
GB15955-PA	0.00054	8.228	8.145	7.970	8.066	2
GB17882-PA	0.00054	9.709	9.670	9.668	9.362	35
gb11399-PA	0.00054	9.512	9.555	9.562	9.320	356
GB12635-PA	0.00054	8.277	7.728	7.781	7.781	123
GB12800-PA	0.00054	8.856	9.371	9.210	9.456	123
GB10219-PA	0.00054	9.246	9.407	9.528	9.568	23
GB14929-PA	0.00054	8.937	9.484	9.167	9.219	13
GB13815-PA	0.00054	9.859	9.957	9.947	10.110	3
GB14864-PA	0.00055	8.750	8.670	8.526	8.518	23
GB16307-PA	0.00055	8.755	8.513	8.600	8.435	13
GB19994-PA	0.00055	10.036	10.387	10.165	10.474	136
GB13102-PA	0.00055	7.681	7.784	7.791	7.873	3
GB14134-PA	0.00055	8.369	8.149	8.196	7.875	3
GB11024-PA	0.00055	8.748	8.679	8.562	8.497	3
GB13413-PA	0.00055	9.522	9.560	9.742	9.705	2
GB13545-PA	0.00056	8.487	8.347	8.384	8.294	3
GB17112-PA	0.00057	8.463	8.576	8.534	8.706	3
GB14523-PA	0.00057	7.733	7.771	7.909	7.974	35
GB12257-PA	0.00057	7.730	7.689	7.621	7.514	3
GB13165-PA	0.00057	10.180	10.154	10.048	9.882	35
GB11685-PA	0.00057	7.949	7.566	7.501	7.349	123
GB17382-PA	0.00057	9.694	8.321	8.424	8.417	123
GB18149-PA	0.00057	9.427	9.259	9.179	9.239	2
GB19734-PA	0.00058	9.393	9.339	9.127	9.140	2345
GB18987-PA	0.00059	10.188	10.294	9.832	10.133	246
GB14600-PA	0.00061	7.698	7.745	7.471	7.430	2345
GB15691-PA	0.00061	8.802	9.875	9.514	9.769	123
GB17851-PA	0.00061	7.821	7.886	7.958	8.238	35
GB18427-PA	0.00062	8.077	7.917	7.884	7.751	3
GB13644-PA	0.00062	7.094	6.726	6.790	6.766	123
GB11093-PA	0.00063	7.581	7.612	7.261	7.335	234
GB13935-PA	0.00063	7.637	7.630	7.317	7.303	2345
GB15953-PA	0.00065	9.945	9.816	9.647	9.635	23
GB11703-PA	0.00065	9.968	10.516	10.633	10.737	123
GB13792-PA	0.00065	6.706	7.002	6.993	7.258	23
GB17941-PA	0.00065	6.986	6.875	6.690	6.755	23
GB18113-PA	0.00065	8.438	8.463	8.389	8.132	35
GB19670-PA	0.00065	10.431	10.363	10.049	9.814	35
GB12602-PA	0.00066	7.454	7.709	7.674	7.840	123
GB19414-PA	0.00066	6.790	6.658	6.701	6.574	3
GB12743-PA	0.00066	7.645	7.632	7.402	7.366	2345
GB19842-PA	0.00067	7.637	7.485	7.340	7.321	23
GB10815-PA	0.00067	10.564	10.715	10.303	10.351	245
GB13758-PA	0.00067	7.180	7.434	7.535	7.639	23
GB11595-PA	0.00067	8.367	8.727	7.944	8.343	4
GB18598-PA	0.00067	7.153	7.165	7.244	7.361	3
GB12316-PA	0.00067	9.323	9.145	9.136	9.045	3
GB11562-PA	0.00067	9.435	9.583	9.617	9.700	23
GB18956-PA	0.00096	8.713	8.801	8.904	8.929	3
GB18239-PA	0.00097	8.763	8.635	8.525	8.489	23
GB14531-PA	0.00067	6.346	6.534	6.571	6.564	23
GB15388-PA	0.00069	7.787	7.421	7.416	7.425	123
GB14859-PA	0.0007	9.730	9.579	9.424	9.454	23
GB18026-PA	0.0007	7.826	7.778	7.565	7.529	235
GB18997-PA	0.00071	7.080	7.229	7.419	7.334	23
GB17028-PA	0.00071	6.790	6.279	6.314	6.416	123
GB20148-PA	0.00073	11.034	10.617	10.414	9.989	23
GB18463-PA	0.00073	10.387	10.565	10.643	10.669	23
GB11240-PA	0.00073	8.091	8.307	8.354	8.311	123
GB20127-PA	0.00074	8.913	8.751	8.622	8.705	23
GB14091-PA	0.00074	10.763	10.536	10.416	10.342	23
GB18312-PA	0.00075	9.338	10.895	10.944	11.916	123
GB13087-PA	0.00075	8.707	8.852	8.825	8.996	3
GB11143-PA	0.00075	8.151	8.110	8.258	8.402	35
GB16256-PA	0.00075	9.772	9.738	9.427	9.461	234
GB10914-PA	0.00075	10.104	9.930	9.966	9.889	3
GB18952-PA	0.00075	10.963	10.754	10.916	10.630	36
GB13959-PA	0.00075	8.229	8.130	7.935	8.041	2
GB17866-PA	0.00075	9.282	9.080	9.098	9.018	3
GB19955-PA	0.00076	8.545	8.463	8.244	8.247	23
GB14857-PA	0.00076	7.743	7.530	7.474	7.359	23
GB14252-PA	0.00077	7.197	6.971	6.959	6.827	123
GB11404-PA	0.00077	9.450	9.448	8.994	8.950	2345
GB17278-PA	0.00078	6.646	7.341	7.427	7.485	123
GB19268-PA	0.00078	8.552	7.796	7.736	7.945	123
GB19704-PA	0.00078	8.024	7.508	7.367	7.150	23
GB15167-PA	0.0008	9.190	9.332	9.388	9.544	23
GB14715-PA	0.0008	8.583	8.711	8.264	8.458	24
GB14291-PA	0.00081	9.475	9.818	9.641	9.810	13
GB18724-PA	0.00082	7.671	7.611	7.542	7.829	56
GB15038-PA	0.00082	8.283	9.316	9.151	8.928	123
GB17007-PA	0.00083	8.258	8.011	8.048	7.989	123
GB10873-PA	0.00083	9.407	9.332	9.238	9.077	35
GB16451-PA	0.00083	7.926	8.080	8.226	8.239	23
GB11929-PA	0.00083	7.617	7.307	7.203	6.977	23
GB18694-PA	0.00083	8.907	8.681	8.761	8.581	13
GB17788-PA	0.00084	8.222	8.358	7.850	7.999	245
GB19598-PA	0.00084	8.913	8.802	8.583	8.574	23
GB15675-PB	0.00085	9.190	9.346	9.348	9.385	3
GB13680-PA	0.00087	12.713	12.447	12.519	12.358	13
GB14300-PA	0.00087	8.717	8.532	8.260	8.211	23
GB19147-PA	0.00087	8.755	8.479	8.331	8.240	23
GB12313-PA	0.00087	10.430	10.201	9.705	9.694	23
GB13909-PA	0.00089	7.971	10.307	10.551	10.892	123
GB16419-PA	0.00089	9.085	9.080	9.072	9.329	356
GB15780-PA	0.00089	8.550	8.730	8.491	8.306	35
GB19211-PA	0.00089	8.025	7.883	7.604	7.722	23
GB16913-PA	0.00091	9.235	9.304	9.402	9.570	35
GB16074-PA	0.00091	6.944	7.151	7.013	7.128	1
GB17959-PA	0.00091	10.333	9.975	9.960	9.636	23
GB16439-PA	0.00091	8.542	8.619	8.678	8.735	3
GB14117-PA	0.00092	9.720	9.412	9.489	9.277	123
GB15057-PA	0.00092	7.168	6.981	7.057	6.928	3
GB18717-PA	0.00094	6.737	6.735	6.808	6.972	356
GB10606-PA	0.00094	7.010	6.709	6.747	6.605	123
GB11252-PA	0.00095	10.020	9.852	9.849	9.650	23
GB19782-PA	0.00096	7.718	7.787	7.785	7.967	3
gb11487-PA	0.00096	7.667	8.013	7.823	8.236	36
GB20055-PA	0.00096	9.930	10.386	10.158	10.401	13
GB10460-PA	0.00096	8.874	10.012	9.892	9.597	123

GB10250-PA	0.00097	7.837	7.960	8.068	8.057	23
GB16613-PA	0.00097	7.361	7.087	7.064	7.058	123
GB13135-PA	0.00098	12.806	12.653	12.500	12.431	23
GB14418-PA	0.00098	7.524	7.074	7.101	7.129	123
GB17285-PA	0.001	7.387	7.194	7.436	7.273	4
GB20055-PD	0.00101	9.985	10.429	10.197	10.444	13
GB15280-PA	0.00101	6.912	7.214	7.203	7.461	3
GB10565-PA	0.00102	9.085	9.248	9.228	9.385	3
GB18277-PA	0.00103	8.762	8.575	8.527	8.540	23
GB19460-PC	0.00106	12.090	11.955	11.629	11.614	235
GB10657-PA	0.00107	10.127	10.119	10.047	9.762	356
GB15874-PA	0.00109	9.106	9.228	9.250	9.452	35
GB16115-PA	0.00109	7.611	7.448	7.399	7.415	2
GB15204-PA	0.0011	8.800	8.797	8.801	9.077	356
GB18592-PA	0.0011	9.933	10.024	9.969	9.661	35
GB10511-PA	0.00111	9.418	9.135	8.953	8.470	35
GB16442-PA	0.00112	9.116	9.064	8.967	8.856	3
GB13012-PA	0.00113	9.674	9.210	9.087	9.188	123
GB14560-PA	0.00114	7.381	6.789	6.808	6.783	123
GB12265-PA	0.00115	6.408	6.136	6.175	6.165	123
GB15558-PA	0.00118	9.622	9.558	9.382	9.339	235
GB10016-PA	0.00119	9.047	10.537	10.557	11.081	123
GB12829-PA	0.00119	9.534	9.385	9.377	9.215	3
GB14178-PA	0.00123	9.874	9.496	9.593	9.253	13
GB19280-PA	0.00123	10.090	9.753	9.764	9.599	123
GB13992-PA	0.00123	9.494	9.293	9.272	9.317	3
GB10251-PA	0.00125	8.400	8.636	8.756	8.620	123
GB14547-PA	0.00125	6.933	7.469	7.365	7.305	123
GB19881-PA	0.00126	9.380	9.380	9.183	9.171	24
GB14388-PA	0.00126	9.321	9.541	9.328	9.505	1
AmOr130-PA	0.00126	6.117	5.972	6.034	5.893	3
GB19253-PA	0.00127	8.977	8.607	8.546	8.601	123
GB10261-PA	0.00128	6.730	6.513	6.494	6.523	123
TAMU:temp8: Chromosome3 _Group3.1	0.00128	10.694	10.807	10.393	10.355	2345
GB14970-PA	0.0013	9.048	9.722	9.395	9.771	13
GB18024-PA	0.0013	8.431	8.744	8.687	9.327	356
GB18471-PA	0.0013	9.013	9.299	9.201	9.310	13
GB14492-PA	0.00131	6.640	7.423	7.242	7.237	123
GB19336-PA	0.00131	6.535	6.669	6.579	7.041	356
GB15009-PA	0.00131	9.745	9.729	9.848	9.982	35
GB17789-PA	0.00131	10.591	10.144	10.007	9.894	123
GB13135-PC	0.00131	12.803	12.651	12.494	12.427	23
GB20055-PB	0.00133	10.006	10.448	10.225	10.461	13
GB17589-PA	0.00133	8.097	8.020	7.966	7.782	35
GB12373-PA	0.00133	8.137	7.883	7.912	7.730	13
GB10140-PA	0.00133	7.564	7.029	7.099	6.901	123
GB16196-PA	0.00136	9.615	10.386	10.517	10.512	123
GB11642-PA	0.00136	9.260	9.315	9.621	9.531	23
GB11018-PA	0.00136	7.742	7.622	7.595	7.509	3
GB17328-PA	0.00137	8.029	8.029	7.673	7.862	24
GB17121-PA	0.00137	7.025	6.950	6.823	6.792	3
GB15848-PA	0.00137	7.053	6.846	6.899	6.772	13
GB20055-PE	0.00138	10.053	10.480	10.270	10.508	13
GB10063-PA	0.00146	8.472	8.529	8.729	8.769	235
GB17231-PA	0.00149	6.516	6.480	6.407	6.154	356
GB16710-PA	0.0015	6.728	6.982	7.236	6.988	23
GB11621-PA	0.0015	9.298	9.045	9.058	9.017	123
GB11679-PA	0.0015	9.924	8.202	8.252	7.778	123
GB10965-PA	0.00151	7.070	7.858	7.838	7.908	123
GB16185-PA	0.00151	10.993	11.235	11.308	11.259	123
GB18132-PA	0.00151	10.125	10.134	9.931	9.839	35
GB10798-PA	0.00151	6.121	6.400	6.346	6.350	123

GB15055-PA	0.00151	13.193	12.398	12.219	11.436	3
GB18059-PA	0.00152	9.622	9.231	9.306	9.309	123
GB16239-PA	0.00152	7.222	7.339	7.406	7.453	3
GB20055-PC	0.00155	9.930	10.364	10.132	10.371	13
GB10287-PA	0.00155	6.263	6.321	6.363	6.084	356
GB10541-PA	0.00156	9.370	9.076	9.135	9.024	123
GB13995-PA	0.00158	9.377	9.053	8.968	8.890	123
GB18655-PA	0.00162	7.713	7.256	7.218	7.246	123
GB10549-PA	0.00165	7.186	7.068	6.803	6.710	235
GB10896-PA	0.00166	10.343	12.205	12.254	11.740	123
GB13976-PA	0.00166	8.195	7.831	7.809	7.783	123
GB20104-PA	0.00167	11.311	10.998	10.928	10.898	123
GB11050-PA	0.00169	6.583	6.484	6.362	6.392	2
GB11407-PA	0.0017	11.986	12.366	12.100	12.361	136
GB10772-PA	0.00171	6.803	6.877	6.506	6.653	24
GB18480-PA	0.00171	6.894	6.655	6.655	6.527	123
GB11400-PA	0.00171	9.459	9.473	9.335	9.063	35
GB18192-PA	0.00171	9.193	9.065	8.932	8.739	23
GB15669-PA	0.00173	9.498	9.540	9.216	9.239	2345
GB15323-PA	0.00175	10.777	11.220	11.329	11.387	123
GB19921-PA	0.00175	9.738	9.652	9.503	9.454	23
GB12214-PA	0.00175	10.734	10.861	10.441	10.419	2345
GB12543-PA	0.00176	9.883	10.529	10.342	10.503	13
GB16811-PA	0.00176	9.695	9.597	9.215	9.507	24
GB13459-PA	0.00177	8.812	9.615	9.401	9.346	123
GB12339-PA	0.00177	8.407	8.484	8.239	8.229	45
GB18489-PA	0.0018	9.586	10.303	9.979	11.596	36
GB17369-PA	0.0018	6.657	6.711	6.743	6.931	35
GB19264-PA	0.00181	7.136	8.033	7.852	7.666	123
GB18308-PA	0.00181	7.301	7.052	7.144	7.066	13
GB12323-PA	0.00181	10.198	9.987	10.128	10.027	1
GB13759-PA	0.00181	6.909	6.809	6.769	6.569	3
GB14329-PA	0.00181	7.446	7.172	7.221	7.174	13
GB13937-PA	0.00181	7.661	7.522	7.448	7.353	23
GB13389-PA	0.00185	9.056	8.947	8.557	8.966	246
GB12280-PA	0.00185	9.405	9.265	9.366	9.099	36
GB18099-PA	0.00185	9.422	9.142	9.169	9.029	123
GB10397-PA	0.00185	9.143	9.382	9.422	9.639	3
GB13129-PA	0.00188	8.462	8.606	8.518	8.676	3
GB14872-PA	0.00188	9.299	9.194	8.751	8.902	234
GB13800-PA	0.00188	11.453	11.052	11.196	10.827	3
GB13494-PA	0.00189	6.095	6.340	6.373	6.257	12
gb11585-PA	0.00189	8.829	8.777	8.559	8.586	234
GB11423-PA	0.0019	10.779	10.670	10.557	10.532	23
GB20034-PA	0.00193	11.079	10.973	10.760	10.703	23
GB16005-PA	0.00196	7.338	7.445	7.088	7.171	245
GB14686-PB	0.00198	8.458	8.449	8.492	8.660	35
GB11864-PA	0.00198	10.615	10.243	10.285	10.191	123
GB12808-PA	0.002	8.407	8.489	8.524	8.631	3
GB14059-PA	0.00202	12.319	11.962	11.907	11.837	123
GB18911-PA	0.00203	7.549	7.350	7.278	7.384	2
GB13312-PA	0.00204	8.168	7.636	7.696	7.751	123
GB18065-PA	0.00204	8.516	8.905	8.627	8.935	13
GB15035-PA	0.00204	7.380	7.247	7.249	7.174	3
GB13655-PA	0.00207	6.752	6.396	6.439	6.538	123
GB18692-PA	0.00208	7.282	7.334	7.471	7.544	35
GB18793-PA	0.00208	7.974	8.236	8.365	8.208	123
GB10211-PA	0.00209	8.164	8.409	8.343	8.458	13
GB19218-PA	0.00209	10.953	10.836	10.758	10.712	23
GB19489-PA	0.00212	7.477	7.228	7.215	7.224	123
GB14317-PA	0.00212	7.272	7.130	7.086	7.054	3

GB10966-PA	0.00212	8.532	8.413	8.257	8.197	23
GB15408-PA	0.00214	9.846	10.014	10.055	10.232	3
GB15934-PA	0.00215	6.593	6.352	6.446	6.384	13
GB19533-PA	0.00216	8.601	8.355	8.392	8.243	13
GB20077-PA	0.00217	7.290	7.404	7.485	7.522	3
GB15147-PA	0.00224	10.216	10.043	9.997	9.905	23
GB11724-PA	0.00224	6.600	6.117	6.187	6.068	123
GB19843-PA	0.00224	6.074	6.130	6.161	6.280	3
GB14868-PA	0.00224	9.788	9.491	9.433	9.395	123
GB19379-PA	0.00224	10.655	10.312	10.283	10.261	123
GB18159-PA	0.00225	6.257	6.051	6.007	5.955	23
GB12303-PA	0.00226	10.316	11.029	10.724	10.738	13
GB13771-PA	0.0023	9.588	9.835	9.655	10.008	36
GB18376-PA	0.0023	9.765	9.821	9.909	10.082	35
GB19290-PA	0.00231	8.645	8.804	8.920	8.930	23
GI:63041825-PA	0.00232	10.419	10.213	10.268	10.193	13
GB15967-PA	0.00232	7.124	7.087	7.123	6.940	36
GB19805-PA	0.00235	9.148	9.237	9.268	9.402	3
GB10400-PA	0.00235	9.067	9.118	8.896	8.911	45
GB18821-PA	0.00235	6.215	6.052	6.055	6.016	3
GB11945-PA	0.00235	6.894	6.741	6.649	6.635	23
GB10314-PA	0.00238	7.749	7.835	7.965	7.953	23
GB19474-PA	0.00239	9.559	10.317	10.513	10.023	123
GB19716-PA	0.00239	8.144	8.398	8.238	8.588	3
GB16902-PA	0.00239	8.020	7.927	7.902	7.766	3
GB14610-PA	0.00241	7.568	7.351	7.454	7.332	13
GB13140-PA	0.00242	8.533	9.985	9.379	10.433	13
GB11944-PA	0.00248	7.726	7.885	7.818	8.047	3
GB14286-PA	0.0025	9.070	9.421	9.294	9.447	13
GB18615-PA	0.0025	8.729	8.536	8.534	8.530	3
GB10315-PA	0.00251	10.989	10.833	10.804	10.751	3
GB15555-PA	0.00252	9.080	9.010	8.833	8.837	23
GB19115-PA	0.00252	6.663	6.615	6.416	6.440	23
GB13804-PA	0.00255	8.215	7.889	7.972	7.865	13
GB15287-PA	0.00255	9.037	9.275	9.136	9.290	13
GB11349-PA	0.00255	8.241	8.398	8.242	8.437	36
GB11933-PA	0.00255	9.193	9.076	8.998	8.829	3
GB19868-PA	0.00255	10.209	9.882	9.949	9.825	13
GB19094-PA	0.00256	6.870	7.245	7.083	7.233	13
GB17250-PA	0.00257	8.873	9.126	9.007	9.410	36
GB15839-PA	0.00259	9.866	9.502	9.584	9.564	123
GB17097-PA	0.00259	6.474	6.208	6.065	6.115	123
GB14906-PA	0.0026	8.801	8.608	8.663	8.600	3
GB12149-PA	0.0026	8.785	8.514	8.674	8.573	13
GB12706-PA	0.00263	9.855	9.764	9.632	9.595	23
GB16214-PA	0.00264	7.724	7.779	7.552	7.523	345
GB19443-PA	0.00267	6.557	6.872	6.825	6.694	12
GB10329-PA	0.00267	11.059	10.745	10.819	10.700	13
GB15416-PA	0.0027	9.945	10.132	10.188	10.327	23
Robert14-PA	0.00272	7.299	7.494	7.480	7.593	23
TAMU:temp26:GroupUn_GroupUn.279...	0.00276	11.908	11.749	11.541	11.496	23
GB16772-PA	0.00277	7.196	7.432	7.290	7.550	3
GB14104-PA	0.00278	9.428	9.599	9.467	9.716	36
GB15078-PA	0.00278	7.950	7.870	7.718	8.150	356
GB11634-PA	0.00279	9.469	9.378	9.119	9.019	23
GB16070-PA	0.00281	9.158	8.621	8.539	8.671	123
GB11226-PA	0.00284	6.528	6.388	6.378	6.288	3
GB13956-PA	0.00284	8.735	8.706	8.562	8.495	23
GB15946-PA	0.00284	7.165	7.040	6.928	6.807	23
GB16507-PA	0.00285	9.117	9.659	9.527	9.843	13
GB13580-PA	0.00285	6.237	6.056	6.018	5.966	23
GB17842-PA	0.00285	7.908	7.718	7.660	7.603	23
GB12465-PA	0.00286	7.296	7.066	6.995	6.831	23
GB19901-PA	0.00287	7.031	7.624	7.176	7.422	13
GB10506-PA	0.00292	7.477	7.126	7.142	7.116	123
GB20029-PA	0.00293	8.054	7.915	7.938	7.851	3
GB16168-PA	0.00293	9.538	9.219	8.922	8.857	23
GB17781-PA	0.00295	6.356	6.286	6.187	6.136	3
GB16939-PA	0.00296	7.179	6.935	7.203	6.597	36
GB20037-PA	0.003	7.035	6.657	6.815	6.668	13
GB12697-PA	0.003	6.980	6.760	6.746	6.608	23
GB18512-PA	0.003	9.507	9.101	8.778	8.440	23
GB14650-PA	0.003	8.394	8.431	8.153	8.245	24
GB19713-PA	0.00302	9.684	9.803	9.962	10.057	23
GB10288-PA	0.00302	8.337	8.301	8.484	8.498	5
GB15552-PA	0.00303	9.972	9.775	9.796	9.651	3
GB16424-PA	0.00304	6.958	7.388	7.307	7.717	3
GB17313-PA	0.00306	8.279	8.366	8.412	8.567	3
GB10071-PA	0.00307	8.250	8.376	8.391	8.549	3
GB15382-PA	0.00307	7.824	8.069	7.713	8.015	14
GB17520-PA	0.00307	8.782	8.821	8.983	8.935	2
GB17118-PA	0.00307	8.344	8.303	8.231	8.089	3
GB17816-PA	0.00307	8.002	8.068	8.131	8.226	3
GB19752-PA	0.00307	8.826	9.024	8.899	9.171	36
GB13836-PA	0.0031	7.968	6.197	6.284	6.026	123
GB15577-PA	0.00313	7.476	7.321	7.313	7.197	3
GB12245-PA	0.00318	8.920	9.169	8.991	9.298	36
GB15861-PA	0.00318	8.475	8.634	8.731	8.758	23
GB11165-PA	0.00319	7.736	7.545	7.558	7.546	3
GB16255-PA	0.00321	7.363	7.254	7.180	7.144	3
gb16134-PA	0.00321	9.365	10.141	10.064	10.610	3
GB18282-PA	0.00324	7.466	7.594	7.913	8.003	235
GB13076-PA	0.00324	7.454	7.335	7.242	7.253	2
GB16702-PA	0.00325	10.152	10.459	10.558	10.409	123
GB17487-PA	0.00325	9.183	8.807	8.847	8.800	123
GB12498-PA	0.00328	10.539	10.326	10.177	10.310	23
GB13238-PA	0.00329	9.298	10.333	10.373	9.883	12
GB19529-PA	0.00332	6.739	6.808	6.589	6.659	4
GB14966-PA	0.00336	8.817	8.648	8.660	8.605	3
GB18867-PA	0.00336	10.652	10.671	10.343	10.382	234
GB17761-PA	0.00338	7.495	7.872	7.657	7.917	13
GB15736-PA	0.0034	10.273	9.967	9.902	9.821	123
GB14290-PA	0.0034	9.279	9.494	9.424	9.407	1
GB19895-PA	0.00341	8.527	8.129	7.955	7.785	23
GB16920-PA	0.00346	7.912	7.997	8.027	8.246	35
GB17229-PA	0.00352	8.002	8.294	8.356	8.339	123
GB12336-PA	0.00355	7.085	7.838	7.683	7.765	13
GB17111-PA	0.00363	8.081	7.780	7.814	7.749	123
GB16986-PA	0.00363	10.593	10.083	10.083	9.886	13
GB16927-PA	0.00363	8.947	8.298	8.148	8.171	123
GB19550-PA	0.00364	7.684	8.570	8.401	9.325	3
GB15468-PA	0.00365	7.766	7.311	7.423	7.143	13
GB12346-PA	0.00373	8.866	8.853	8.582	8.613	23
GB16645-PA	0.00375	9.159	9.065	8.967	8.940	3
GB16757-PA	0.00375	10.595	10.218	10.148	10.211	123
GB11670-PA	0.00379	7.397	7.412	7.499	7.603	3
GB15981-PA	0.00379	7.322	7.336	7.165	7.123	5
GB16799-PA	0.00382	7.815	8.159	7.876	8.123	13
GB17733-PA	0.00389	10.688	10.023	9.881	10.152	123
GB11511-PA	0.00391	6.358	7.974	7.852	7.159	12

GB10618-PA	0.00392	7.472	7.243	7.180	7.240	123
GB18782-PA	0.00394	7.962	7.496	7.624	7.317	13
GB11387-PA	0.00395	7.822	7.483	7.488	7.529	123
GB12470-PA	0.00395	9.160	8.990	8.933	8.949	23
GB15070-PA	0.00395	8.513	7.244	6.928	7.263	123
GB15904-PA	0.00396	7.239	7.124	7.081	7.011	3
GB19936-PA	0.004	9.315	9.083	9.170	9.126	13
GB19066-PA	0.00404	6.913	7.069	7.066	7.104	3
GB14820-PA	0.00404	7.075	6.812	6.770	6.567	3
GB11184-PA	0.00406	6.797	6.529	6.639	6.577	13
GB18857-PA	0.00407	8.787	8.610	8.554	8.627	2
GB17418-PA	0.00417	6.426	6.676	6.595	6.617	13
GB15107-PA	0.00418	8.620	8.752	8.779	8.896	3
GB13470-PA	0.00421	11.628	12.536	12.398	12.857	123
GB12951-PE	0.00421	8.012	8.225	8.124	8.273	136
GB18429-PA	0.00424	7.805	7.893	7.982	8.018	3
GB12937-PA	0.00425	9.163	10.275	9.792	10.398	13
GB12662-PA	0.00428	8.468	9.151	9.116	9.317	123
GB18219-PA	0.0043	8.519	9.602	9.490	9.787	123
GB19207-PA	0.00435	9.958	10.407	10.205	11.135	36
GB18284-PA	0.0044	9.939	9.951	9.599	9.622	235
GB15968-PA	0.00445	6.492	6.761	6.681	6.888	13
GB15060-PA	0.00445	9.081	8.807	9.039	8.434	36
GB19429-PA	0.00445	9.405	9.272	9.064	9.112	23
GB19184-PA	0.00445	7.717	7.512	7.553	7.627	1
GB16040-PA	0.00446	6.281	6.489	6.485	6.615	3
GB19059-PA	0.00446	6.978	6.668	6.704	6.718	123
GB14655-PA	0.00446	6.433	6.177	6.115	6.142	123
GB11650-PA	0.0045	6.077	6.554	6.274	6.323	13
GB18585-PB	0.00454	6.722	6.418	6.393	6.269	123
TAMU:temp5:GroupUn_GroupUn.48:1...	0.00458	10.831	10.692	10.635	10.358	35
GB11198-PA	0.00459	8.216	7.964	7.834	7.956	23
GB12367-PA	0.0046	7.405	7.136	7.216	7.029	13
GB17603-PA	0.00471	8.675	8.538	8.616	8.474	3
GB11848-PA	0.00471	7.765	7.447	7.366	7.271	23
GB14683-PA	0.00478	7.317	6.835	6.867	6.806	123
GB19957-PA	0.00491	7.950	7.964	7.679	7.677	235
maleszka3-PA	0.00499	8.482	7.280	6.938	7.256	123
GB15364-PA	0.00501	8.379	8.122	7.840	7.778	23
GB11391-PA	0.00509	9.027	9.697	9.478	9.913	13
GB17031-PA	0.00512	8.644	8.262	8.405	8.213	13
GB14572-PA	0.00514	9.032	9.218	9.367	9.383	23
GB19341-PA	0.00515	10.706	10.454	10.559	10.418	13
GB14266-PA	0.00518	8.751	8.349	8.488	8.509	13
GB11271-PA	0.00521	6.877	6.841	6.910	6.600	36
GB12564-PA	0.00522	7.188	7.607	7.459	7.674	13
GB13568-PA	0.00523	7.064	6.759	6.734	6.658	123
GB12479-PA	0.00529	9.167	8.895	9.001	8.982	1
GB17013-PA	0.00534	6.778	7.389	7.221	7.418	13
GB16422-PA	0.00534	8.646	8.480	8.472	8.434	3
GB15145-PA	0.00534	8.357	8.165	8.321	8.114	3
GB15799-PA	0.00536	8.888	8.943	9.084	9.173	35
GB17305-PA	0.00536	9.835	9.660	9.550	9.582	23
GB13563-PA	0.00539	8.352	8.142	8.228	8.121	13
GB11375-PA	0.00541	6.722	7.391	7.340	7.234	123
GB11881-PA	0.00548	7.503	7.297	7.392	7.374	1
GB14989-PA	0.00549	9.939	9.535	9.591	9.312	3
AmOr128-PA	0.00556	8.058	7.749	7.860	7.519	3
GB14037-PA	0.00556	12.263	12.821	12.736	12.775	123
GB11282-PA	0.00557	9.082	9.344	9.336	9.230	12

GB16085-PA	0.0056	6.769	6.696	6.469	6.579	2
GB12590-PA	0.00565	9.338	9.028	9.123	9.097	123
GB15398-PA	0.00571	7.710	8.295	8.107	8.361	13
GB10273-PA	0.00574	8.974	8.755	8.809	8.690	13
GB17032-PA	0.00579	10.525	11.537	11.688	11.664	123
GB18918-PA	0.0058	8.649	8.907	8.778	9.014	3
GB19286-PA	0.0058	8.464	8.410	8.238	8.240	23
GB20070-PA	0.00581	11.479	10.756	10.603	9.968	3
GB12087-PA	0.00586	9.120	8.915	8.927	8.802	3
AmOr71-PA	0.00586	6.481	6.245	6.222	5.913	3
GB14663-PA	0.00586	9.029	10.378	10.609	10.488	123
GB17965-PA	0.00586	8.017	8.137	8.230	8.348	3
GB14940-PA	0.00586	7.307	8.325	7.878	8.660	13
GB10855-PA	0.00586	6.640	6.307	6.261	6.204	123
GB18648-PA	0.00586	9.575	9.380	9.306	9.356	23
GB17449-PA	0.00593	8.120	7.916	7.961	7.887	13
GB17712-PA	0.00602	10.628	10.948	10.987	11.097	23
GB10017-PA	0.00606	9.021	9.472	9.245	9.451	13
GB16366-PA	0.00606	7.070	6.898	6.861	6.786	23
GB11309-PA	0.00607	6.567	6.885	6.861	6.956	123
GB16958-PA	0.00607	8.882	9.292	9.102	9.202	13
GB15901-PA	0.00614	8.205	7.971	7.987	8.016	12
GB16622-PA	0.00617	8.623	8.435	8.400	8.352	23
GB15230-PA	0.00621	11.382	12.176	11.875	12.174	13
GB12419-PA	0.00621	10.415	10.105	10.037	9.895	23
GB10234-PA	0.00623	10.172	9.958	10.030	9.949	13
GB16183-PA	0.00625	10.545	10.741	10.866	10.843	23
GB19871-PA	0.00633	7.614	7.268	7.366	7.275	13
GB18626-PA	0.00636	7.682	6.427	6.478	6.591	123
GB15862-PA	0.00642	7.607	7.709	7.814	7.913	3
GB16709-PA	0.00658	8.965	9.264	9.257	9.476	3
GB11816-PA	0.00658	6.514	6.894	6.764	6.774	13
GB12385-PA	0.00658	8.814	8.969	9.029	9.017	3
GB19212-PA	0.00658	7.349	7.019	7.130	7.038	13
GB18100-PA	0.0066	7.223	7.434	7.395	7.572	3
GB16381-PA	0.00668	9.620	10.950	10.891	10.966	123
GB20154-PA	0.0067	9.062	9.062	8.856	8.861	24
GB15367-PA	0.00672	8.086	7.877	7.775	7.853	23
GB11737-PA	0.00673	7.043	7.165	7.046	7.290	36
GB19314-PA	0.00685	10.085	9.953	9.760	9.814	23
AmelCPR17-PA	0.00696	9.676	11.024	10.944	11.036	123
GB11786-PA	0.00697	10.728	10.488	10.480	10.450	123
GB10686-PA	0.007	8.518	8.328	8.259	8.357	2
GB10718-PA	0.007	6.752	6.746	6.533	6.528	23
GB11304-PA	0.007	9.419	10.093	9.982	10.456	3
GB11479-PA	0.007	8.941	8.752	8.555	8.584	23
GB11335-PA	0.00701	8.365	8.825	8.699	8.931	13
GB18157-PA	0.00705	9.564	10.489	10.282	11.082	3
GB19238-PA	0.00705	9.742	9.936	10.011	9.946	123
GB10869-PA	0.00713	10.259	10.956	10.862	10.851	123
GB12158-PA	0.00713	6.698	6.648	6.581	6.471	35
GB17797-PA	0.00714	7.025	6.668	6.795	6.742	13
GB19461-PA	0.00716	8.424	8.166	8.207	8.071	13
GB16735-PA	0.00726	5.992	5.913	5.999	6.344	35
GB11840-PA	0.00731	9.154	9.621	9.558	9.877	3
GB10037-PA	0.00731	7.104	6.633	6.713	6.734	123
GB11167-PA	0.00731	9.090	8.905	8.785	8.764	23
GB12201-PA	0.00733	8.156	8.663	8.849	8.709	123
GB13256-PA	0.00742	9.000	8.807	8.807	8.763	3
GB10141-PA	0.00745	8.296	8.369	8.505	8.477	2

GB16960-PA	0.00747	7.831	6.753	6.880	6.637	123
GB19204-PA	0.00754	7.097	7.084	7.276	7.318	35
GB14057-PA	0.00754	7.272	7.127	7.119	7.019	3
GB10317-PA	0.00754	7.003	6.675	6.771	6.727	13
GB10427-PA	0.00754	8.003	7.847	7.768	7.772	23
GB12428-PA	0.00774	6.161	5.990	6.043	5.941	3
GB19471-PA	0.00774	10.049	9.879	9.849	9.785	3
GB17929-PA	0.00775	9.718	9.684	9.691	9.489	356
GB19055-PA	0.00778	5.882	6.276	6.106	6.518	3
GB17105-PA	0.00778	7.101	7.281	7.299	7.419	3
GB12920-PA	0.00783	10.239	9.954	9.981	9.933	123
GB16243-PA	0.00786	6.390	6.164	6.123	6.214	12
GB10571-PA	0.00788	7.176	6.972	7.007	6.945	13
GB19698-PA	0.00788	6.374	6.678	6.594	6.731	13
GB12673-PA	0.00794	7.217	6.877	7.018	6.863	13
GB17545-PA	0.00796	7.038	7.014	6.764	6.884	24
GB19517-PA	0.00798	7.447	7.258	7.309	7.207	3
GB11478-PA	0.00801	10.195	10.192	9.990	9.917	35
GB12968-PA	0.00807	8.295	8.265	7.834	8.031	24
GB11426-PA	0.00811	8.650	9.601	9.593	9.425	123
GB14990-PA	0.00811	7.829	7.085	7.150	7.114	123
GB11058-PA	0.00812	7.673	8.042	8.013	8.380	3
GB11333-PA	0.00818	6.565	6.286	6.399	6.295	13
GB14377-PA	0.0083	6.694	6.545	6.491	6.483	3
GB13423-PA	0.0083	6.962	6.702	6.802	6.690	13
GB15214-PA	0.00835	10.218	11.768	11.726	11.833	123
GB16287-PA	0.00837	6.512	7.480	7.313	7.625	13
GB12249-PA	0.00837	8.186	8.556	8.346	8.648	3
GB16839-PA	0.00837	7.256	7.519	7.571	7.648	23
GB11785-PA	0.00837	7.322	6.940	7.141	6.970	13
GB13605-PA	0.00838	9.432	9.868	9.756	9.928	13
GB11091-PA	0.00844	8.279	8.120	8.049	7.996	23
GB15084-PA	0.00845	9.588	10.881	10.851	11.221	123
GB15906-PA	0.00845	10.625	11.924	11.613	12.183	13
gb12004-PA	0.00846	10.670	11.138	10.824	11.097	13
GB16786-PA	0.00847	7.759	7.974	7.886	8.013	13
GB11607-PA	0.00849	7.157	7.487	7.625	7.422	123
GB13202-PA	0.00849	7.465	7.360	7.163	7.117	23
GB13311-PA	0.00849	9.252	9.580	9.743	9.585	23
GB17685-PA	0.00849	8.264	8.328	8.457	8.493	23
GB19139-PA	0.00849	6.271	6.064	6.084	6.070	13
GB18173-PA	0.00849	8.479	8.266	8.264	8.214	123
GB15493-PA	0.00853	6.234	6.504	6.431	6.461	123
GB13495-PA	0.00855	9.474	9.324	9.232	9.308	2
GB17628-PA	0.00859	7.413	7.589	7.568	7.677	3
GB18230-PA	0.00859	8.494	8.237	8.240	8.293	123
GB12314-PA	0.00862	8.513	8.919	8.769	8.849	13
GB16822-PA	0.00867	10.073	10.135	9.848	9.755	35
GB12453-PA	0.00868	7.732	6.338	6.496	6.096	123
GB11565-PA	0.00869	7.852	8.076	8.169	8.080	123
GB14040-PA	0.00879	9.355	9.460	9.443	9.544	3
GB15965-PA	0.00881	8.567	8.862	8.752	8.746	1
GB13283-PA	0.00888	9.505	9.208	9.296	9.176	13
GB12718-PA	0.00896	9.240	8.615	8.431	8.514	123
GB12005-PA	0.00904	6.495	6.428	6.424	6.667	56
GB11972-PA	0.00909	9.681	9.990	9.901	10.004	13
GB14770-PA	0.00913	7.889	7.949	7.644	7.622	35
GB12624-PA	0.00913	7.004	6.749	6.680	6.644	23
GB13701-PA	0.00913	8.134	7.719	7.860	7.674	13
GB19561-PA	0.00914	8.338	9.561	9.501	9.200	123
GB11615-PA	0.00917	8.962	9.135	9.176	9.245	23
GB10722-PA	0.00924	7.960	8.251	8.313	8.338	23

GB13977-PA	0.00927	7.259	7.002	7.095	7.010	13
GB15706-PA	0.00928	7.620	7.296	7.300	7.237	123
GB18888-PA	0.00928	6.402	6.198	6.266	6.248	1
GB18518-PA	0.00929	7.205	7.487	7.326	7.514	3
GB17822-PA	0.00935	6.559	6.143	6.189	6.148	123
GB16117-PA	0.00939	8.133	8.439	8.487	8.479	123
GB17468-PA	0.0094	7.569	7.333	7.423	7.383	1
GB12199-PA	0.00944	6.361	6.068	6.122	5.959	13
GB16013-PA	0.00944	8.561	8.578	8.235	8.093	35
GB12809-PA	0.00944	7.936	6.470	6.659	6.181	13
GB16350-PA	0.00948	9.058	8.886	8.888	8.807	3
GB16457-PA	0.00955	8.461	8.533	8.159	8.260	24
GB14689-PA	0.00962	7.519	8.091	8.120	8.120	123
GB15747-PA	0.00983	9.492	9.209	9.265	9.210	13
GB18254-PA	0.00988	8.287	8.687	8.534	8.493	13
GB14780-PA	0.00988	7.548	7.237	7.151	7.349	12
GB13003-PA	0.00988	11.340	11.137	11.041	11.048	23
GB13698-PA	0.01003	8.737	8.331	8.467	8.359	13
GB19347-PA	0.01008	9.591	10.911	10.694	11.649	3
GB12592-PA	0.01011	8.550	8.195	8.205	8.218	123
GB11633-PA	0.01011	8.818	8.290	8.206	8.170	123
GB13929-PA	0.01019	9.535	9.281	9.319	9.245	123
GB17447-PA	0.01019	7.141	7.127	6.922	6.772	3
GB19197-PA	0.01024	7.264	6.929	6.962	6.895	123
GB10705-PA	0.01025	8.519	9.015	8.894	9.097	13
GB11843-PA	0.01025	7.274	7.374	7.654	7.696	23
GB19342-PA	0.01025	8.023	7.808	7.742	7.842	12
GB17611-PA	0.01025	7.389	7.090	7.167	7.179	13
GB10995-PC	0.01031	6.680	7.265	7.031	7.406	13
GB13714-PA	0.01031	7.783	7.499	7.432	7.506	123
GB11596-PA	0.01041	8.084	8.791	8.797	8.555	123
GB14457-PA	0.01044	8.834	8.655	8.596	8.564	23
GB11228-PA	0.01051	10.313	10.004	10.086	10.076	13
GB11300-PA	0.01066	9.050	9.504	9.239	9.436	3
GB16236-PA	0.01066	8.604	8.614	8.718	8.976	35
GB15835-PA	0.01066	6.742	6.986	6.847	6.991	13
GB15330-PA	0.01073	6.409	6.200	6.100	6.244	2
GB11409-PA	0.01079	7.894	7.837	7.690	7.507	3
GB17574-PA	0.01081	7.114	6.734	6.793	6.801	123
GB13388-PA	0.01082	10.538	10.932	10.800	10.948	13
GB13700-PA	0.0109	8.668	9.324	8.897	9.152	13
GB19701-PA	0.01092	8.712	8.954	9.032	9.218	3
GB18817-PA	0.01093	8.206	7.731	7.772	7.747	123
GB15509-PA	0.01096	8.220	8.883	8.886	8.683	123
GB13902-PA	0.01096	9.189	8.717	8.769	8.489	3
GB16716-PA	0.01103	10.348	8.971	8.992	8.383	3
GB17017-PA	0.01114	6.242	6.511	6.462	6.619	3
GB14412-PA	0.01116	8.531	8.277	8.377	8.209	13
GB19596-PA	0.01117	7.701	8.250	7.807	8.035	13
GB19407-PA	0.01117	8.856	8.601	8.639	8.662	13
maleszka1-PA	0.0112	6.975	7.429	7.387	7.482	13
GB15994-PA	0.01121	8.999	9.519	9.619	9.426	123
GB18839-PA	0.01126	8.382	8.100	8.132	8.155	123
GB19606-PA	0.01128	7.779	8.684	8.432	9.084	3
GB11661-PA	0.01128	6.312	6.129	6.041	5.963	23
GB17542-PA	0.01131	7.263	7.084	6.951	6.929	23
GB15928-PA	0.01134	6.721	6.900	7.014	6.971	23
GB14309-PA	0.0114	7.053	8.182	8.390	8.108	123
GB19142-PA	0.0114	7.485	8.012	7.765	8.188	3
GB17857-PA	0.0114	8.312	8.228	8.132	8.077	3
GB19667-PA	0.0114	7.894	8.573	8.488	8.226	12
GB11815-PA	0.01141	11.709	11.368	11.429	11.310	13

GB20008-PA	0.01142	8.688	8.409	8.563	8.482	13
GB13707-PA	0.01142	6.972	6.593	6.596	6.460	23
GB12657-PA	0.01142	6.543	5.794	5.868	5.829	123
GB19662-PA	0.01148	8.133	9.066	9.111	9.147	123
GB18022-PA	0.01155	9.869	10.922	10.378	11.014	13
GB15345-PA	0.01166	10.320	11.115	11.113	10.898	123
GB11022-PA	0.01166	7.379	7.150	7.160	7.051	3
GB14368-PA	0.0117	7.974	8.361	8.384	8.339	123
GB14740-PA	0.01171	8.174	9.247	8.853	9.634	3
GB16269-PA	0.01179	7.275	6.967	7.067	6.965	13
GB11421-PA	0.01188	8.851	8.517	8.072	8.160	23
GB16667-PB	0.01199	7.645	7.900	7.846	8.053	3
GB11743-PA	0.01202	6.681	7.492	7.297	7.142	13
GB10636-PA	0.01205	8.486	8.656	8.739	8.810	23
GB16248-PA	0.01206	9.067	8.802	8.877	8.889	1
GB17107-PA	0.01216	7.135	7.295	7.386	7.393	23
GB16994-PA	0.0122	7.760	8.162	7.954	8.131	13
GB13768-PA	0.01223	7.410	7.181	7.246	7.141	13
GB10995-PB	0.01227	8.391	9.121	8.802	9.464	3
GB12567-PA	0.01227	10.545	10.879	10.933	10.898	123
GB10215-PA	0.01232	9.734	9.347	9.369	9.246	13
GB13501-PA	0.01246	6.712	6.425	6.444	6.240	3
GB13130-PA	0.01254	8.673	8.509	8.504	8.443	3
GB19865-PA	0.01258	7.683	7.557	7.491	7.429	3
GB10753-PA	0.01263	5.782	6.037	6.089	6.102	123
GB14660-PA	0.01267	7.439	7.252	7.143	7.071	23
GB17253-PA	0.01287	6.266	6.500	6.458	6.467	13
GB15921-PA	0.01298	7.734	8.294	8.319	8.910	3
GB11910-PA	0.01309	8.341	7.698	7.740	7.638	13
GB11354-PA	0.01343	7.205	7.533	7.438	7.593	13
GB12402-PA	0.01343	9.027	9.428	9.418	9.391	123
GB13671-PA	0.01343	6.972	7.346	7.258	7.228	13
GB14067-PA	0.01344	8.222	8.600	8.505	8.728	3
GB18459-PA	0.01344	8.941	8.925	8.611	8.673	23
GB15199-PA	0.01345	7.294	7.407	7.567	7.528	23
GB15056-PA	0.01345	6.967	6.637	6.737	6.638	13
GB15012-PA	0.01368	7.138	7.429	7.328	7.327	13
GB13823-PA	0.01368	8.479	8.252	8.050	8.054	23
GB12866-PA	0.01378	7.910	8.089	8.058	8.133	3
GB16667-PC	0.01379	8.172	8.427	8.355	8.556	3
GB10880-PA	0.01383	6.643	7.161	6.985	7.219	13
GB17968-PA	0.01395	7.664	7.438	7.453	7.377	13
GB15664-PA	0.01412	11.045	11.400	11.473	11.357	123
GB14384-PA	0.0142	8.831	10.072	10.114	9.770	123
GB11088-PA	0.01428	7.851	7.573	7.562	7.410	3
GB15529-PA	0.01443	7.298	7.008	7.032	6.923	3
GB10695-PA	0.01461	7.361	6.935	7.002	6.916	123
GB16810-PA	0.01488	10.220	9.915	9.969	9.983	13
GB10927-PA	0.01502	8.002	7.697	7.792	7.705	13
GB10188-PA	0.01518	5.946	6.126	6.265	6.169	23
maleszka5-PA	0.01522	7.515	7.382	7.209	7.180	23
GB16369-PA	0.01523	7.527	8.608	8.086	8.167	1
GB15269-PA	0.01532	9.867	9.474	9.478	9.418	123
GB10640-PA	0.01543	7.064	6.733	6.698	6.628	3
GB16723-PA	0.0155	6.627	6.880	6.809	6.887	13
GB16666-PA	0.01555	8.245	8.647	8.417	8.708	13

GB10871-PA	0.01564	9.843	9.477	9.495	9.428	13
GB13350-PA	0.01571	7.204	7.425	7.495	7.765	3
GB14288-PA	0.01578	9.690	9.280	9.382	9.309	13
GB11159-PA	0.01596	7.564	7.256	7.302	7.243	13
GB12745-PA	0.01598	6.890	6.090	6.291	6.032	13
GB10459-PA	0.01603	7.822	8.282	8.241	8.509	3
GB16522-PA	0.01606	7.684	7.458	7.544	7.446	13
GB15583-PA	0.01613	7.215	7.554	7.508	7.567	13
GB13643-PA	0.01622	7.132	7.629	7.674	7.881	3
GB14044-PA	0.01641	8.566	8.381	8.141	7.889	3
GB16667-PA	0.01647	8.070	8.354	8.265	8.449	3
GB17692-PA	0.01661	8.699	9.435	9.501	9.679	23
GB16349-PA	0.01661	6.535	6.360	6.303	6.281	23
GB16520-PA	0.01665	10.199	9.999	10.003	9.957	3
AmelCPR16-PA	0.01665	8.254	6.837	7.040	6.844	123
GB12667-PA	0.01695	7.193	6.898	6.939	6.819	3
GB16163-PA	0.01711	7.736	7.553	7.578	7.542	3
GB14954-PA	0.01721	7.299	7.691	7.517	7.689	13
GB15903-PA	0.01725	8.713	8.478	8.506	8.525	13
GB14847-PA	0.01735	7.527	7.199	7.254	7.284	13
GB19110-PA	0.01739	8.359	9.051	8.993	9.505	3
GB20121-PA	0.01768	9.751	8.993	8.906	9.003	123
GB16009-PA	0.01772	6.806	7.114	7.050	7.096	13
GB10584-PA	0.01783	8.063	8.789	8.605	8.729	13
Pigment-Dispersing_Hormone-PA	0.01794	7.140	6.645	6.661	6.563	13
GB17163-PA	0.01806	6.575	7.051	6.997	7.071	123
GB12989-PA	0.01816	9.694	9.495	9.508	9.489	3
GB20132-PA	0.01823	6.165	6.505	6.452	6.459	13
GB11664-PA	0.01841	10.170	10.805	10.815	10.840	123
GB19543-PA	0.01848	8.298	7.992	7.963	7.881	3
GB10905-PA	0.01848	7.985	7.733	7.540	7.255	3
GB18453-PA	0.01856	6.847	7.431	7.422	7.371	123
GB11907-PA	0.01864	7.920	8.316	8.098	8.261	13
GB19149-PA	0.01866	6.334	6.311	6.295	6.543	356
GB15360-PA	0.01869	8.151	7.856	7.865	7.868	13
GB12114-PA	0.01901	8.882	8.424	8.385	8.424	123
GB11753-PA	0.01973	7.800	8.347	8.084	8.579	3
GB16774-PA	0.01978	7.105	7.837	7.709	8.269	3
TAMU:temp16:GroupUn_GroupUn.69:...	0.01993	10.129	10.679	10.509	10.619	13
GB13865-PA	0.01994	8.085	8.519	8.277	8.559	3
GB10517-PA	0.01994	6.888	6.979	6.485	6.542	3
GB16534-PA	0.01999	9.963	9.644	9.550	9.674	23
GB19319-PA	0.02007	9.088	8.874	8.916	8.920	1
GB13446-PA	0.02013	7.567	8.277	8.440	8.351	23
GB12889-PA	0.02019	8.372	8.080	8.095	7.904	3
GB19587-PA	0.02025	9.388	10.202	10.085	10.196	123
GB19630-PA	0.02033	8.623	8.390	8.450	8.464	1
GB17910-PA	0.02035	7.440	7.279	7.163	7.122	23
GB14208-PA	0.02035	8.791	8.484	8.245	8.156	23
GB18334-PA	0.02043	9.020	10.508	10.438	11.386	3
GB19372-PA	0.02043	7.824	8.204	8.187	8.462	3
GB10441-PA	0.02053	7.568	8.507	8.290	9.224	3
GB15988-PA	0.02068	7.602	8.564	8.360	9.446	3
GB18896-PA	0.02092	7.921	8.756	8.682	9.731	3
GB19303-PA	0.02095	9.404	9.011	9.118	8.957	13

GB11716-PA	0.02104	7.296	7.820	8.025	7.733	2
GB19800-PA	0.02106	8.808	9.168	9.022	9.582	3
GB13093-PA	0.02116	8.349	9.712	9.531	9.471	13
GB13882-PA	0.02161	6.883	7.105	7.263	7.454	3
GB17575-PA	0.02237	6.785	6.576	6.489	6.391	3
GB10708-PA	0.02241	7.576	6.463	6.710	6.134	3
GB12607-PA	0.02242	8.483	7.793	7.522	7.457	3
GB11053-PA	0.02251	8.363	8.078	8.102	8.025	3
GB16900-PA	0.02258	6.071	6.350	6.435	7.233	35
GB10223-PA	0.02263	6.751	7.124	7.078	7.358	3
GB15749-PA	0.02263	9.074	8.732	8.614	8.707	23
GB17401-PA	0.02268	8.267	8.200	8.198	8.065	3
GB12354-PA	0.02296	8.016	7.838	7.858	7.826	3
GB15069-PA	0.02313	9.739	9.384	9.271	9.207	23
GB15941-PA	0.02321	8.566	8.959	8.885	8.953	13
GB16876-PA	0.02327	8.124	8.650	8.425	8.801	3
GB17258-PA	0.02338	8.391	8.651	8.585	8.692	13
GB13933-PA	0.02346	8.182	7.900	7.771	7.713	23
GB12438-PA	0.02373	6.488	6.177	6.199	6.193	123
GB17015-PA	0.02385	7.988	7.725	7.864	8.189	56
GB15587-PA	0.0239	7.116	6.817	6.817	6.898	123
GB19454-PA	0.02399	7.423	8.155	8.102	8.538	3
GB12399-PA	0.02408	7.005	6.750	6.800	6.700	3
GB16461-PA	0.0241	7.115	7.871	7.537	8.039	3
GB17436-PA	0.02422	6.679	6.889	6.859	6.920	13
GB13660-PA	0.02429	7.127	6.859	6.884	6.893	13
GB18027-PA	0.02434	7.631	7.451	7.494	7.412	3
GB11350-PA	0.02501	8.041	7.833	7.827	7.749	3
GB12536-PA	0.02508	6.908	7.469	7.219	7.594	3
GB19237-PA	0.02514	6.685	6.965	6.936	6.974	13
GB19888-PA	0.02515	10.813	10.824	10.872	10.584	36
GB19203-PA	0.02516	7.179	6.724	6.663	6.402	3
GB13090-PA	0.02517	6.817	6.620	6.656	6.611	3
GB13484-PA	0.02572	9.221	10.155	9.904	10.350	3
GB19434-PA	0.02572	7.375	7.041	7.095	7.162	1
GB10704-PA	0.02579	10.160	10.454	10.549	10.594	3
GB16377-PA	0.02582	7.584	8.046	7.971	8.313	3
GB14391-PA	0.02588	7.001	7.365	7.279	7.466	3
GB14074-PA	0.02669	8.017	8.000	7.969	7.837	3
GB15001-PA	0.02699	8.512	8.271	8.327	8.314	13
GB17257-PA	0.02702	7.679	8.183	8.089	8.384	3
GB19642-PA	0.02712	8.170	8.917	8.848	9.430	3
GB16100-PA	0.02715	8.552	8.846	8.896	8.880	23
GB13602-PA	0.02759	7.820	8.288	8.419	8.227	23
GB11494-PA	0.02861	6.881	7.178	7.297	7.233	23
GB15712-PA	0.02866	6.697	6.467	6.537	6.474	13
GB10167-PA	0.02892	7.692	7.542	7.511	7.493	3
GB18208-PA	0.02939	8.381	8.046	8.108	8.097	13
GB16782-PA	0.02942	7.147	6.914	6.932	6.959	13
GB17908-PA	0.03154	8.722	8.478	8.472	8.493	123
GB17018-PA	0.03155	6.964	7.466	7.466	7.435	123
GB18585-PA	0.03209	6.937	6.447	6.607	6.338	3
GB12100-PA	0.03212	8.002	7.808	7.855	7.811	3
GB15657-PA	0.03244	8.778	9.645	9.626	10.157	3
Hh-PA	0.03258	8.059	7.478	7.529	7.163	3
GB10048-PA	0.03283	6.857	6.650	6.650	6.587	3
GB12097-PA	0.03427	7.774	7.420	7.507	7.448	13
GB14843-PA	0.03433	7.918	7.672	7.725	7.673	13
GB14741-PA	0.03462	7.245	7.892	7.893	7.895	13
GB17823-PA	0.0348	7.259	6.952	6.813	6.955	3

GB10076-PA	0.03482	7.755	7.536	7.561	7.506	3
GB19816-PA	0.03488	9.826	9.408	9.367	9.352	3
GB13795-PA	0.03517	6.850	6.545	6.642	6.632	13
GB16675-PA	0.03529	6.259	6.806	6.715	6.620	1
GB19627-PA	0.0353	7.404	7.089	7.187	7.147	13
GB18389-PA	0.03616	7.088	6.755	6.813	6.821	13
GB15794-PA	0.03623	7.614	6.991	7.050	6.778	3
GB15467-PA	0.03632	8.921	8.686	8.685	8.626	3
GB20014-PA	0.03751	8.004	8.741	8.717	8.821	3
GB14574-PA	0.03836	8.090	7.537	7.569	7.211	3
GB16881-PA	0.0398	7.802	8.316	8.273	8.455	3
GB19250-PA	0.04082	9.222	10.833	11.193	10.606	3
GB17654-PA	0.04171	8.078	7.571	7.446	7.517	23
GB19798-PA	0.04209	8.311	8.016	8.015	7.973	3
GB12454-PA	0.0424	9.233	10.269	10.057	10.617	3
GB18709-PA	0.04323	7.833	8.448	8.443	8.575	3
GB16684-PA	0.04444	8.279	8.720	8.690	8.765	3
GB14511-PA	0.04487	5.832	6.821	6.970	7.670	3
GB11371-PA	0.04567	7.216	7.631	7.690	7.747	3
GB13199-PA	0.04862	8.271	8.840	8.987	8.848	23
GB10563-PA	0.05582	7.593	8.527	8.257	8.651	3
GB16883-PA	0.05807	8.597	7.163	7.311	6.857	3
GB18300-PA	0.05903	8.105	8.862	8.790	9.305	3
GB13559-PA	0.06227	7.642	8.388	8.396	8.631	3
Sylvain4-PA	0.06239	8.687	11.472	11.256	10.683	1
GB11280-PA	0.07247	5.723	6.617	6.858	6.760	3
GB16475-PA	0.0751	8.407	7.265	7.558	7.253	3
GB15865-PA	0.10814	6.264	7.187	7.309	8.099	3
