# Supplementary material for: A Virulent Strain of Deformed Wing Virus (DWV) of Honeybees (Apis mellifera) Prevails after Varroa destructor-Mediated, or In Vitro, Transmission
Source: PLoS Pathog. 2014 Jun 26;10(6):e1004230. doi: 10.1371/journal.ppat.1004230 (PMC4072795; doi:10.1371/journal.ppat.1004230)
Supplement: Table S3 — Summary of gene expression commonality between the contrasts. Number of differentially expressed genes common to pairs of contrasts. In each cell (corresponding to the pair of contrasts) the three figures show the commonality for all differentially expressed genes in the pair, the up-regulated genes in the pair, and the down-regulated genes in the pair respectively. (PDF) [file ppat.1004230.s010.pdf]

Table S3. Summary of gene expression commonality between the contrasts.

All Genes (n=1054)

All, ↑, ↓	NVvC	VLvC	VHvC	VLvNV	VHvNV	VHvVL
NVvC	416,192,224	220,96,124	385,177,208	1,0,0	1,0,0	7,6,0
VLvC		493,153,340	444,141,303	51,1,50	57,4,52	3,0,0
VHvC			951,393,558	34,1,33	133,48,85	64,42,22
VLvNV				59,2,57	27,0,27	2,0,0
VHvNV					148,53,95	42,29,13
VHvVL						71,48,23

Immune-related genes

All, ↑, ↓	NVvC	VLvC	VHvC	VLvNV	VHvNV	VHvVL
NVvC	18,11,7	12,7,5	17,10,7	0,0,0	0,0,0	0,0,0
VLvC		26,7,19	22,6,16	6,0,6	2,0,2	0,0,0
VHvC			42,14,28	4,0,4	6,0,6	3,0,3
VLvNV				7,0,7	3,0,3	0,0,0
VHvNV					7,1,6	3,1,2
VHvVL						4,1,3

Number of differentially expressed genes common to pairs of contrasts. In each cell (corresponding to the pair of contrasts) the three figures show the commonality for all differentially expressed genes in the pair, the up-regulated genes in the pair, and the down-regulated genes in the pair respectively.
