# Supplementary material for: A Virulent Strain of Deformed Wing Virus (DWV) of Honeybees (Apis mellifera) Prevails after Varroa destructor-Mediated, or In Vitro, Transmission
Source: PLoS Pathog. 2014 Jun 26;10(6):e1004230. doi: 10.1371/journal.ppat.1004230 (PMC4072795; doi:10.1371/journal.ppat.1004230)
Supplement: Table S4 — Significantly overrepresented GO terms (Biological Process, BP, terms) in the contrasts (corrected p -value <0.05). (PDF) [file ppat.1004230.s011.pdf]

Table S4. Significantly overrepresented GO terms (Biological Process, BP, terms) in the contrasts.
(7344 Apis mellifera genes used in GO analysis, corrected p-value < 0.05)

GO Term	GO ID	Gene count	Number of genes associated with GO term	Corrected P-value
**C to NV contrast (all DE genes, 269 genes used in GO analysis)**				
DNA conformation change	GO:0071103	21	127	1.60E-05
organelle fission	GO:0048285	26	209	4.49E-05
nuclear division	GO:0000280	25	200	4.49E-05
DNA packaging	GO:0006323	17	98	4.49E-05
chromosome organization	GO:0051276	47	570	4.49E-05
M phase of mitotic cell cycle	GO:0000087	26	218	4.49E-05
mitosis	GO:0007067	24	196	6.84E-05
protein-DNA complex assembly	GO:0065004	14	70	6.84E-05
metaphase plate congression	GO:0051310	7	15	1.27E-04
M phase	GO:0000279	35	384	1.27E-04
cell cycle phase	GO:0022403	47	604	1.27E-04
mitotic cell cycle	GO:0000278	43	540	2.11E-04
amino acid transmembrane transport	GO:0003333	11	48	2.16E-04
spindle assembly involved in mitosis	GO:0090307	5	7	2.87E-04
mitotic sister chromatid segregation	GO:0000070	14	86	4.83E-04
nucleosome assembly	GO:0006334	10	43	4.83E-04
establishment of chromosome localization	GO:0051303	7	19	5.02E-04
chromosome localization	GO:0050000	7	19	5.02E-04
response to stimulus	GO:0050896	154	3204	5.59E-04
sister chromatid segregation	GO:0000819	14	91	7.58E-04
mitotic metaphase plate congression	GO:0007080	6	14	8.15E-04
spindle assembly	GO:0051225	7	23	1.76E-03
cell cycle process	GO:0022402	50	754	2.64E-03
cellular component assembly	GO:0022607	65	1082	2.88E-03
nucleoside triphosphate metabolic process	GO:0009141	26	296	3.55E-03
organelle organization	GO:0006996	84	1537	4.34E-03
chromatin assembly	GO:0031497	10	58	4.79E-03
purine nucleoside triphosphate metabolic process	GO:0009144	25	286	4.83E-03
chromosome segregation	GO:0007059	17	154	4.83E-03
heterocycle metabolic process	GO:0046483	37	518	6.20E-03
coagulation	GO:0050817	23	257	6.34E-03
mitotic spindle organization	GO:0007052	9	50	6.94E-03
hemostasis	GO:0007599	23	264	9.01E-03
response to wounding	GO:0009611	35	494	1.04E-02
chromosome condensation	GO:0030261	7	32	1.13E-02
wound healing	GO:0042060	26	324	1.13E-02
response to stress	GO:0006950	95	1863	1.18E-02
spindle organization	GO:0007051	11	80	1.18E-02
blood coagulation	GO:0007596	22	254	1.18E-02
microtubule depolymerization	GO:0007019	3	4	1.51E-02
cell cycle	GO:0007049	52	871	1.59E-02
nucleosome organization	GO:0034728	10	70	1.59E-02
transmembrane transport	GO:0055085	28	373	1.62E-02
amino acid transport	GO:0006865	13	114	1.81E-02
ATP metabolic process	GO:0046034	16	161	1.81E-02
chromatin assembly or disassembly	GO:0006333	11	86	1.88E-02
purine ribonucleoside triphosphate metabolic process	GO:0009205	23	284	1.88E-02
cytokinesis	GO:0000910	14	132	2.11E-02
telomere maintenance	GO:0000723	9	61	2.21E-02
ribonucleoside triphosphate metabolic process	GO:0009199	23	289	2.22E-02

regulation of body fluid levels	GO:0050878	23	289	2.22E-02
macromolecular complex subunit organization	GO:0043933	53	913	2.22E-02
telomere organization	GO:0032200	9	63	2.61E-02
macromolecular complex assembly	GO:0065003	45	745	2.74E-02
monocarboxylic acid metabolic process	GO:0032787	21	258	2.77E-02
cellular component biogenesis	GO:0044085	69	1294	2.81E-02
ribonucleoside triphosphate catabolic process	GO:0009203	21	261	2.96E-02
purine ribonucleoside triphosphate catabolic process	GO:0009207	21	261	2.96E-02
purine nucleoside triphosphate catabolic process	GO:0009146	21	261	2.96E-02
nucleoside triphosphate catabolic process	GO:0009143	21	261	2.96E-02
ATP catabolic process	GO:0006200	14	141	3.26E-02
carnitine transport	GO:0015879	4	12	3.50E-02
betaine transport	GO:0015838	4	12	3.50E-02
purine ribonucleotide metabolic process	GO:0009150	23	304	3.62E-02
purine ribonucleotide catabolic process	GO:0009154	21	267	3.62E-02
nucleobase, nucleoside and nucleotide catabolic process	GO:0034656	23	305	3.62E-02
nucleobase, nucleoside, nucleotide and nucleic acid catabolic process	GO:0034655	23	305	3.62E-02
ribonucleotide catabolic process	GO:0009261	21	268	3.70E-02
purine nucleotide catabolic process	GO:0006195	22	288	3.86E-02
sister chromatid biorientation	GO:0031134	3	6	4.09E-02
cellular nitrogen compound catabolic process	GO:0044270	23	310	4.27E-02
attachment of spindle microtubules to chromosome	GO:0051313	4	13	4.30E-02
purine nucleotide metabolic process	GO:0006163	25	351	4.52E-02
nucleotide catabolic process	GO:0009166	22	293	4.52E-02
chromatin organization	GO:0006325	28	412	4.58E-02
G1/S transition of mitotic cell cycle	GO:0000082	10	86	4.69E-02
**C to VH contrast (all genes, 630 genes used in GO analysis)**				
amino acid transmembrane transport	GO:0003333	17	48	9.25E-04
protein-DNA complex assembly	GO:0065004	19	70	9.26E-03
monocarboxylic acid metabolic process	GO:0032787	44	258	9.52E-03
nuclear division	GO:0000280	36	200	1.34E-02
organelle fission	GO:0048285	37	209	1.34E-02
response to stimulus	GO:0050896	324	3204	1.79E-02
amino acid transport	GO:0006865	24	114	1.79E-02
nucleosome assembly	GO:0006334	13	43	1.84E-02
response to organic substance	GO:0010033	133	1133	1.84E-02
organic acid metabolic process	GO:0006082	72	531	1.84E-02
M phase	GO:0000279	56	384	1.84E-02
mitosis	GO:0007067	34	196	1.90E-02
microtubule depolymerization	GO:0007019	4	4	1.90E-02
DNA conformation change	GO:0071103	25	127	1.98E-02
cell cycle phase	GO:0022403	79	604	1.98E-02
chromosome organization	GO:0051276	75	570	2.23E-02
spindle assembly involved in mitosis	GO:0090307	5	7	2.23E-02
M phase of mitotic cell cycle	GO:0000087	36	218	2.23E-02
oxoacid metabolic process	GO:0043436	69	516	2.27E-02
carboxylic acid metabolic process	GO:0019752	69	516	2.27E-02
carboxylic acid transport	GO:0046942	32	187	2.37E-02
cellular ketone metabolic process	GO:0042180	70	530	2.65E-02
mitotic cell cycle	GO:0000278	71	540	2.65E-02
organic acid transport	GO:0015849	32	190	2.82E-02
positive regulation of cellular catabolic process	GO:0031331	14	56	3.48E-02
DNA packaging	GO:0006323	20	98	3.48E-02
mitotic spindle organization	GO:0007052	13	50	3.69E-02
triglyceride biosynthetic process	GO:0019432	12	44	3.81E-02
carbohydrate catabolic process	GO:0016052	19	93	4.19E-02
coagulation	GO:0050817	39	257	4.19E-02
acylglycerol biosynthetic process	GO:0046463	12	45	4.19E-02
neutral lipid biosynthetic process	GO:0046460	12	45	4.19E-02

**C to VH contrast (excluding genes common with C to NV contrast, 385 genes used in GO analysis)**				
none				
**C to VL contrast (all DE genes, 339 genes used in GO analysis)**				
protein-DNA complex assembly	GO:0065004	14	70	1.05E-02
**C to VL contrast (excluding genes common with C to NV contrast, 197 genes used in GO analysis)**				
regulation of gene expression	GO:0010468	72	1608	2.61E-03
regulation of RNA metabolic process	GO:0051252	61	1287	2.61E-03
regulation of transcription	GO:0045449	61	1301	2.61E-03
regulation of primary metabolic process	GO:0080090	88	2164	2.61E-03
regulation of transcription, DNA-dependent	GO:0006355	58	1230	3.21E-03
regulation of macromolecule metabolic process	GO:0060255	83	2029	3.35E-03
regulation of cellular metabolic process	GO:0031323	90	2300	5.84E-03
regulation of cellular biosynthetic process	GO:0031326	69	1618	6.34E-03
regulation of macromolecule biosynthetic process	GO:0010556	65	1502	7.09E-03
regulation of biosynthetic process	GO:0009889	69	1639	7.96E-03
regulation of nitrogen compound metabolic process	GO:0051171	72	1741	8.10E-03
regulation of nucleobase, nucleoside, nucleotide and nucleic acid metabolic process	GO:0019219	70	1688	9.41E-03
regulation of metabolic process	GO:0019222	93	2501	1.77E-02
regulation of developmental process	GO:0050793	44	923	1.77E-02
Wnt receptor signaling pathway	GO:0016055	10	87	2.14E-02
cell surface receptor linked signaling pathway	GO:0007166	42	898	3.61E-02
protein amino acid autophosphorylation	GO:0046777	11	114	3.83E-02
regulation of anatomical structure morphogenesis	GO:0022603	23	384	3.83E-02
organ morphogenesis	GO:0009887	38	791	3.83E-02
pattern specification process	GO:0007389	23	389	4.39E-02
embryonic morphogenesis	GO:0048598	27	500	4.92E-02
regulation of fungal-type cell wall organization	GO:0060237	3	6	4.92E-02
response to cortisol stimulus	GO:0051414	3	6	4.92E-02
**NV to VH contrast (all genes, 104 genes used in GO analysis)**				
none				
**NV to VL contrast (all DE genes, 39 genes used in GO analysis)**				
regulation of cellular metabolic process	GO:0031323	25	2300	1.91E-02
protein amino acid autophosphorylation	GO:0046777	6	114	1.91E-02
regulation of gene expression	GO:0010468	20	1608	2.40E-02
regulation of transcription, DNA-dependent	GO:0006355	17	1230	2.40E-02
regulation of primary metabolic process	GO:0080090	23	2164	2.40E-02
regulation of metabolic process	GO:0019222	25	2501	2.40E-02
regulation of RNA metabolic process	GO:0051252	17	1287	2.40E-02
regulation of macromolecule metabolic process	GO:0060255	22	2029	2.40E-02
regulation of transcription	GO:0045449	17	1301	2.40E-02
cell fate commitment	GO:0045165	7	238	2.97E-02
cell fate commitment involved in the formation of primary germ layers	GO:0060795	3	24	2.97E-02
regulation of macromolecule biosynthetic process	GO:0010556	18	1502	2.97E-02
regulation of cell morphogenesis	GO:0022604	7	246	2.97E-02
positive regulation of glucose metabolic process	GO:0010907	3	25	3.04E-02
neural crest cell fate commitment	GO:0014034	3	26	3.19E-02
positive regulation of carbohydrate metabolic process	GO:0045913	3	29	3.91E-02
positive regulation of cellular carbohydrate metabolic process	GO:0010676	3	29	3.91E-02
**VL to VH contrast (all DE genes, 45 genes used in GO analysis)**				
none				
