# Supplementary material for: A Virulent Strain of Deformed Wing Virus (DWV) of Honeybees (Apis mellifera) Prevails after Varroa destructor-Mediated, or In Vitro, Transmission
Source: PLoS Pathog. 2014 Jun 26;10(6):e1004230. doi: 10.1371/journal.ppat.1004230 (PMC4072795; doi:10.1371/journal.ppat.1004230)
Supplement: Table S5 — Summary of the small RNA sequencing in the experimental groups. The single read libraries were aligned using Bowtie [37] to Apis mellifera miRNA [36], and to the reference full-length DWV and VDV-1 sequences, GenBank Accession numbers GU109335 and AY251269 respectively. (PDF) [file ppat.1004230.s012.pdf]

Table S5. Summary of the small RNA sequencing in the experimental groups.

Experimental group	C	NV	NV	VL	VL	VH	VH
Small RNA library ID (as in ArrayExpress E-MTAB-1671)	Y1	YN2	YN6	YL4	YL8	YH3	YH7
Total reads	11087883	16459758	13102754	28406811	32232127	26047266	35064538
Total DWV and VDV-1 reads	540	1001	828	9844	11513	900332	1198197
miRNA reads	1585149	2655959	2084357	5110815	5572315	3160162	4179594
Proportion of miRNA reads	14.3%	16.1%	15.9%	18.0%	17.3%	12.1%	11.9%
DWV and VDV-1 reads per 1000 miRNAs reads	0.341	0.377	0.397	1.926	2.066	284.901	286.678
Sense DWV and VDV-1 reads of total viral	75%	81%	74%	73%	73%	79%	79%
Antisense DWV and VDV-1 reads of total viral	25%	18%	26%	27%	27%	21%	21%
18 nt sense reads	12	30	32	141	134	10553	14064
19 nt sense reads	10	42	29	200	220	17273	22713
20 nt sense reads	38	62	47	488	561	46599	61970
21 nt sense reads	78	135	94	1467	1697	160157	216985
22 nt sense reads	171	275	230	3941	4649	401273	532019
23 nt sense reads	26	55	50	416	501	32333	43047
24 nt sense reads	17	28	21	106	132	7292	9616
25 nt sense reads	7	43	24	99	97	5656	7592
26 nt sense reads	9	29	19	85	76	5804	7938
27 nt sense reads	9	25	15	81	85	5933	7952
28 nt sense reads	10	29	23	68	85	5653	7350
29 nt sense reads	7	27	14	61	56	5839	7659
30 nt sense reads	13	26	17	61	92	6444	8563
18 nt antisense reads	2	2	3	42	47	2184	3049
19 nt antisense reads	4	9	5	80	127	5705	7818
20 nt antisense reads	9	15	31	246	302	16295	21826
21 nt antisense reads	33	55	31	607	702	45594	60388
22 nt antisense reads	78	102	122	1484	1774	111186	146427
23 nt antisense reads	7	3	12	155	145	7581	9989
24 nt antisense reads	0	3	4	6	19	553	707
25 nt antisense reads	0	1	0	2	9	131	174
26 nt antisense reads	0	0	1	3	1	64	90
27 nt antisense reads	0	1	1	1	0	46	56
28 nt antisense reads	0	2	2	2	1	51	70
29 nt antisense reads	0	0	1	1	0	54	64
30 nt antisense reads	0	2	0	1	1	79	71
DWV coverage (nt)	9434	12934	10327	114715	138700	9906028	13212790
VDV-1 coverage (nt)	6715	11507	9123	123321	142988	10850253	14609501
Coverage ratio, DWV/VDV-1	1.405	1.124	1.132	0.930	0.970	0.913	0.904

The single read libraries were aligned using Bowtie to the *Apis mellifera* miRNA and to the reference full-length DWV and VDV-1 sequences, GeneBank Accession numbers GU109335 and AY251269 respectively.
