# Supplementary material for: A Virulent Strain of Deformed Wing Virus (DWV) of Honeybees (Apis mellifera) Prevails after Varroa destructor-Mediated, or In Vitro, Transmission
Source: PLoS Pathog. 2014 Jun 26;10(6):e1004230. doi: 10.1371/journal.ppat.1004230 (PMC4072795; doi:10.1371/journal.ppat.1004230)
Supplement: Table S6 — Correlation of virus-specific siRNA coverage between experimental groups. Pearson correlations, P<0.001 are shown. The small RNA libraries determined by high-throughput sequencing were aligned to the DWV or VDV-1 sequences (GenBank Accession numbers GU109335 and AY251269 respectively) using bowtie [37]. All reads VDV-1 or DWV pileup values numbers of the DWV- and VDV-1- specific small RNA reads, up to 3 mismatches were allowed for the 18 nt seed region. (PDF) [file ppat.1004230.s013.pdf]

Table S6. Virus-specific siRNA coverage, Pearson correlation, P < 0.001.

DWV-specific siRNAs

	C-1	NV-1	NV-2	VL-1	VL-2	VH-1
**NV-1**	0.666					
**NV-2**	0.593	0.706				
**VL-1**	0.786	0.768	0.737			
**VL-2**	0.775	0.770	0.748	0.958		
**VH-1**	0.768	0.764	0.732	0.957	0.962	
**VH-2**	0.765	0.751	0.726	0.955	0.963	0.997

VDV-1-specific siRNAs

	C-1	NV-1	NV-2	VL-1	VL-2	VH-1
**NV-1**	0.526					
**NV-2**	0.399	0.558				
**VL-1**	0.651	0.706	0.662			
**VL-2**	0.627	0.713	0.646	0.938		
**VH-1**	0.639	0.724	0.683	0.960	0.945	
**VH-2**	0.635	0.726	0.688	0.962	0.950	0.996
