# Supplementary material for: A Virulent Strain of Deformed Wing Virus (DWV) of Honeybees (Apis mellifera) Prevails after Varroa destructor-Mediated, or In Vitro, Transmission
Source: PLoS Pathog. 2014 Jun 26;10(6):e1004230. doi: 10.1371/journal.ppat.1004230 (PMC4072795; doi:10.1371/journal.ppat.1004230)
Supplement: Table S7 — Summary of the NGS libraries and consensus viral sequences from individual honeybees from Varroa -infested colony. RNA-seq libraries were produced using poly(A) RNA extracts. The reads were aligned to the reference full-length DWV and VDV-1 sequences, GenBank Accession numbers GU109335 and AY251269 respectively, using the “ —very-sensitive-local” option which allowed the highest number of mismatches. The aligned reads were used to generate consensus nucleotide sequences. The assembled viral sequences showed highest identity with the DWV-VDV-1 recombinant clone identified in the sampled colony (GenBank Accession number KJ437447) and the group VH sequences (e.g. GenBank Accession number JX661656). (PDF) [file ppat.1004230.s014.pdf]

Table S7. Summary of the NGS libraries and consensus viral sequences from individual honeybees from Varroa-infested colony.

Sample	Total NGS reads	Total DWV and VDV-1 reads	Proportion of DWV and VDV-1 reads	Corrected Average Shannons's diversity index		Nucleotide identity with KJ437447	Amino acid identity with KJ437447 ORF (positions 1145-9826)	Nucleotide identity with VH group sequence JX661656 (positions 4926-5255)	Amino acid identity with VH group sequence JX661656
				CP region	NS region				
Control pupa 1	9768526	8066	0.083%	0.04535	0.05480	98.74%	99.76%	98.57%	100%
Control pupa 2	11494319	6257	0.054%	0.04195	0.04690	98.38%	99.24%	98.42%	100%
Buffer-injected pupa 1	10284715	5775	0.056%	0.04870	0.05645	98.71%	99.76%	98.65%	100%
Buffer-injected pupa 2	9080182	3285	0.036%	0.05015	0.05290	98.01%	99.27%	98.27%	100%
Asymptomatic nurse honeybee 1	7896051	9080	0.115%	0.04200	0.04150	98.92%	99.79%	98.35%	100%
Asymptomatic nurse honeybee 2	9405711	5664	0.060%	0.04185	0.04690	98.30%	99.65%	98.35%	100%
Virus-injected pupa 1	10199537	755669	7.409%	0.01710	0.01470	99.16%	99.83%	98.50%	100%
Virus-injected pupa 2	11277902	1367858	12.129%	0.01575	0.01305	99.16%	99.83%	98.50%	100%
Virus-injected pupa 3	10253990	922841	9.000%	0.01610	0.01375	99.16%	99.83%	98.50%	100%
Symptomatic nurse honeybee 1	8919720	6382279	71.552%	0.01000	0.00980	99.16%	99.83%	98.50%	100%
Symptomatic nurse honeybee 2	9998633	6767911	67.688%	0.01155	0.01330	98.59%	99.59%	99.47%	100%
Symptomatic nurse honeybee 3	8900999	5145729	57.811%	0.01450	0.01290	99.67%	99.76%	99.85%	100%
Symptomatic nurse honeybee 4	9556537	8015413	83.874%	0.01765	0.02120	99.17%	99.79%	98.57%	100%

RNA-seq libraries were produced using poly(A) RNA extracts. The reads were aligned to the reference full-length DWV and VDV-1 sequences, GenBank Accession numbers GU109335 and AY251269 respectively, using the “ --very-sensitive-local “ option which allowed the highest number of mismatches. The aligned reads were used to generate consensus nucleotide sequences. The assembled viral sequences showed highest identity with the DWV-VDV-1 recombinant clone identified in the sampled colony (GenBank Accession number KJ437447) and the group VH sequences (e.g. GenBank Accession number JX661656).
