# Supplementary material for: A Virulent Strain of Deformed Wing Virus (DWV) of Honeybees (Apis mellifera) Prevails after Varroa destructor-Mediated, or In Vitro, Transmission
Source: PLoS Pathog. 2014 Jun 26;10(6):e1004230. doi: 10.1371/journal.ppat.1004230 (PMC4072795; doi:10.1371/journal.ppat.1004230)
Supplement: Table S8 — Differential expression of putative homeobox genes in the contrasts. (PDF) [file ppat.1004230.s015.pdf]

Table S8. Differential expression of putative homeobox genes.

BeeBase ID	Drosophila homolog ID	Gene	Fold change in contrast (Log2 value)					
			C to NV	C to VH	C to VL	NV to VH	NV to VL	VL to VH
GB18585	FBgn0000099	apterous	.	-0.5735201	.	.	.	.
GB15698	FBgn0000625	eyegone	-0.2951224	-0.5625597	.	.	.	.
GB10341	FBgn0000015	Abdominal B	.	-0.5591549	-0.3959795	-0.3581762	.	.
GB15837	FBgn0000611	extradenticle	.	-0.4891224	-0.4226791	-0.3704909	.	.
GB14165	FBgn0011701	reversed polarity		0.3483972	.	.	.	0.3480016
