# Supplementary material for: A Virulent Strain of Deformed Wing Virus (DWV) of Honeybees (Apis mellifera) Prevails after Varroa destructor-Mediated, or In Vitro, Transmission
Source: PLoS Pathog. 2014 Jun 26;10(6):e1004230. doi: 10.1371/journal.ppat.1004230 (PMC4072795; doi:10.1371/journal.ppat.1004230)
Supplement: Text S1 — Next generation sequencing: error correction and diversity testing outline. (PDF) [file ppat.1004230.s016.pdf]

Text S1. Next generation sequencing: error correction and diversity testing outline.

i) Correction for next-generation sequencing error

An error model is assumed for the mutation process, calibrated using the next-generation sequencing of a known clonal sample and then used for correction.

ii) Confidence interval for average nucleotide diversity

a) The variance of a single diversity is found using the Δ-method.

b) The variance of mean diversity is found, accommodating the correlation between adjacent diversities.

c) Hence a 95% confidence interval can be calculated.

iii) Threshold for clonal diversity

a) The error rate after next-generation sequencing correction is estimated using the clonal sample.

b) Simulation is used to generate individual, and then mean, diversity values, based on error at the corrected rate.

c) The 95th percentile of the distribution of mean diversities is the required threshold.
